# Anaesthesia Management for Awake Craniotomy: Systematic Review and Meta-Analysis

**DOI:** 10.1371/journal.pone.0156448

**Published:** 2016-05-26

**Authors:** Ana Stevanovic, Rolf Rossaint, Michael Veldeman, Federico Bilotta, Mark Coburn

**Affiliations:** 1 Department of Anaesthesiology, University Hospital RWTH Aachen, Aachen, Germany; 2 Department of Neurosurgery, University Hospital RWTH Aachen, Aachen, Germany; 3 Department of Anaesthesiology, Critical Care and Pain Medicine, University of Rome “La Sapienza”, Rome, Italy; Scientific Inst. S. Raffaele Hosp., ITALY

## Abstract

**Background:**

Awake craniotomy (AC) renders an expanded role in functional neurosurgery. Yet, evidence for optimal anaesthesia management remains limited. We aimed to summarise the latest clinical evidence of AC anaesthesia management and explore the relationship of AC failures on the used anaesthesia techniques.

**Methods:**

Two authors performed independently a systematic search of English articles in PubMed and EMBASE database 1/2007-12/2015. Search included randomised controlled trials (RCTs), observational trials, and case reports (n>4 cases), which reported anaesthetic approach for AC and at least one of our pre-specified outcomes: intraoperative seizures, hypoxia, arterial hypertension, nausea and vomiting, neurological dysfunction, conversion into general anaesthesia and failure of AC. Random effects meta-analysis was used to estimate event rates for four outcomes. Relationship with anaesthesia technique was explored using logistic meta-regression, calculating the odds ratios (OR) and 95% confidence intervals [95%CI].

**Results:**

We have included forty-seven studies. Eighteen reported asleep-awake-asleep technique (SAS), twenty-seven monitored anaesthesia care (MAC), one reported both and one used the awake-awake-awake technique (AAA). Proportions of AC failures, intraoperative seizures, new neurological dysfunction and conversion into general anaesthesia (GA) were 2% [95%CI:1–3], 8% [95%CI:6–11], 17% [95%CI:12–23] and 2% [95%CI:2–3], respectively. Meta-regression of SAS and MAC technique did not reveal any relevant differences between outcomes explained by the technique, except for conversion into GA. Estimated OR comparing SAS to MAC for AC failures was 0.98 [95%CI:0.36–2.69], 1.01 [95%CI:0.52–1.88] for seizures, 1.66 [95%CI:1.35–3.70] for new neurological dysfunction and 2.17 [95%CI:1.22–3.85] for conversion into GA. The latter result has to be interpreted cautiously. It is based on one retrospective high-risk of bias study and significance was abolished in a sensitivity analysis of only prospectively conducted studies.

**Conclusion:**

SAS and MAC techniques were feasible and safe, whereas data for AAA technique are limited. Large RCTs are required to prove superiority of one anaesthetic regime for AC.

## Introduction

### Rationale

Awake craniotomy (AC) was initially used for removal of epileptic foci with simultaneous application of brain mapping and electrical current. Since the 1980s further developments brought this technique into use for resection of tumours involving functional cortex [1]. AC with live intraoperative brain mapping and monitoring of neurological function and neurocognitive performance, allows maximal resection of malignant gliomas with a favourable survival prognosis and without language deficits [2]. Tumour resection is adapted to the individual anatomy of the patient, which generally shows huge inter-individual variability [2,3]. The primary aim is to preserve or even improve the complex human brain function, while achieving maximal removal of tumours or epileptic foci [4]. Given the effectiveness of AC for resection of eloquent tumours, data suggest an expanded role for AC in brain tumour surgery regardless of tumour location [5]. In addition, ACs are established functional neurosurgical approaches for deep-brain stimulation within treatment of Parkinson´s disease and obsessive-compulsive disorders [6,7]. Recent data suggest that postoperative deficits are less frequent compared to general anaesthesia (GA) [5]. Yet, there is an array of tasks, which have to be accomplished by the anaesthesiologist to avoid complications during ACs. Although anaesthesia for AC is usually well tolerated it requires an extensive knowledge of the principles underlying neuroanaesthesia and the special technical strategies including local anaesthesia for scalp blockade, advanced airway management, dedicated sedation protocols, and skilful management of haemodynamics [7]. One systematic review performed in 2013, focused on the anaesthesia technique for craniotomy [5]. They included only eight studies, published until 2012, which compared GA to AC, but the anaesthetic approach used for AC was not analysed in detail [5]. Nowadays the mainly used anaesthetic techniques for AC include the asleep-awake-asleep (SAS) technique, monitored anaesthesia care (MAC), and the recent introduced awake-awake-awake (AAA) method. SAS is the oldest technique, using GA before and after brain mapping. MAC, also called "conscious sedation" is a mild form of sedation, where the patients`anxiety and pain are controlled, while the patients are able to follow orders and to protect their airways without invasive airway devices [8]. AAA technique only consists of local or regional anaesthesia supplemented with intravenous analgesia but avoiding any sedative anaesthetic. Still, no consensus exists on the optimal anaesthesiological management for AC. In consequence, we decided to analyse the recent evidence of benefits and harms resulting from the different anaesthesia techniques for AC.

### Objectives

We aimed to add to existing knowledge about the process of anaesthesia care for AC, the benefits and harms of the three anaesthesia techniques (MAC, SAS and AAA) for adult patients, from clinical studies published between January 2007 and December 2015. The primary outcome of interest was the incidence of AC failures, related to the used anaesthesia technique. We reviewed the study-, patient-, anaesthesia- and intraoperative-characteristics, including adverse events and postoperative outcomes.

## Materials and Methods

### Protocol

A protocol with the inclusion and exclusion criteria for suitable studies and the method of analysis were established with all authors. The protocol was not published. This systematic review was prepared in accordance with the PRISMA guidelines [9] (see S1 Checklist).

### Registration

This systematic review (SR) was registered in the International Prospective Register of Systematic Reviews (PROSPERO; http://www.crd.york.ac.uk/PROSPERO, CRD42015025376).

### Eligibility criteria

Types of studies: Publication types suitable for inclusion were randomised controlled clinical trials (RCTs), prospective and retrospective observational clinical trials, and case reports with more than four clinical cases. We excluded animal studies, reviews, paediatric studies, studies on pregnant women, other topics, abstracts, letters, and Non-English publications.

Types of participants: The included studies had to report on patients undergoing AC for resection of epileptic foci and tumours that involve eloquent (motor, sensory and language) brain cortices. The studies should be performed in an operating room and describe the anaesthetic approach used for AC. Additionally, they had to report data for at least one of the following outcome variables: intraoperative seizures, hypoxia and arterial hypertension, intra-/ postoperative nausea and vomiting (PONV), new postoperative neurological dysfunction, conversion to GA and failure of AC. Of note, only studies reporting on adult patients (≥18 years of age) were initially considered. It became apparent, that some studies, even with the applied children excluding filter in our search strategy, also reported on AC procedures in several children. Despite this, the mean age in these studies corresponded to adults and after discussion with all authors we decided not to exclude these studies, as they were not real solely paediatric studies.

Types of intervention: We included studies, which reported on one of the following three anaesthetic approaches: Asleep-awake-asleep (SAS) technique, monitored anaesthesia care (MAC), and the awake-awake-awake (AAA) technique.

### Information sources

A PubMed and EMBASE database search was carried out for the time frame from 01.01.2007 until 31.12.2015. The search and screening process were independently carried out by MC and AS. Additionally the reference lists of the included articles were scanned for further eligible studies. One author was contacted, to provide additional study information [10].

### Systematic search

EMBASE and PubMed search strategy are shown in S1 File. Records identified through PubMed and EMBASE were hand-searched on basis of the title and abstract. Resulting records were then hand-searched on basis of the full text and records not matching the topic of this SR were excluded. Of note, articles reporting studies conducted outside the operating room, like in a MRI suite, or with the use of an intraoperative MRI guidance were excluded.

#### Study selection and data collection

MC and AS screened the titles independently and removed articles that did not meet the pre-specified screening criteria, or were duplicate articles. The remaining articles were screened on the basis of their abstract. All apparently eligible articles were analysed in detail, according to a pre-piloted form, by their full text. Any uncertainties were discussed between the two primary review authors. In the event of persistent disagreement, all other authors were integrated in the discussion until consensus was achieved. Articles were also double checked for not apparently study-duplicates, in regard to juxtaposed author names, treatment comparisons, sample sizes, and outcomes. It was planned to contact the authors, if important outcome parameter are missing, and the study met our inclusion criteria.

#### Data items

AS and MC extracted the following data from each included study: 1.) Study characteristics (study design, recruitment period, sample size, comparative group, endpoint/ aim of the study, study conclusion). 2.) Anaesthesia characteristics (kind of technique, drugs and dosages, patient airway). 3.) Patient characteristics (gender, age and kind of tumour). 4.) Intraoperative characteristics and adverse events (surgery durations, AC failures, conversion to GA, hypoxia, arterial hypertension and seizures). An AC failure was not only considered if conversion to GA was required, but also if adequate awake brain mapping/ monitoring could not have been achieved due to the patient condition e.g. seizures, dysphasia, somnolence, agitation or physical complications. 5.) Patient outcomes (including neurological dysfunctions, mortality, postoperative intracranial haematoma, amount of total tumour resection and the length of hospital stay). Our initial protocol sought to precise the postoperative neurological outcomes into subtypes like hemiplegia, hemiparesis, verbal dysfunctions etc., but the systematic search yielded a high diversity in the reported subtypes. Therefore, we decided with all authors to make a simplification into "new neurological dysfunction". This term included all kinds of neurological dysfunctions, but excluded deterioration of pre-existing neurological dysfunctions. RR, FB and MV checked independently the extracted data.

#### Risk of bias in individual studies

For randomised controlled trials we used the Cochrane Collaboration’s risk of bias tool [11]. For observational trials and case reports we used the Agency for Healthcare Research and Quality (AHRQ) tool [12]. Risk of bias was assessed by MC and AS independently during the data extraction process and revealed an adequate reliability.

#### Summary measures and synthesis of results

Our aim was to analyse multiple outcomes of AC patients, depending on the used anaesthesia technique. Our primary outcome of interest was the incidence of AC failure associated with the used anaesthesia techniques. The secondary outcomes included the complication rates, probably related to the used anaesthesia technique.

Pooled estimates of outcome measures with subgroup analyses depending on the anaesthetic approach were calculated if enough studies reported an outcome variable for the respective anaesthesia technique. This referred to the outcome variables AC failure, intraoperative seizure, conversion into GA and new neurological dysfunction. The DerSimonian-Laird random effects model using logit-transformed event proportions was applied, as we assumed a high within study and inter-study variation. The inter-study variation attributed to other reasons than chance was quantified by I^2^. The relationship of anaesthesia technique (MAC/ SAS) as one potential source of heterogeneity and the four above-described outcome measures (AC failure, intraoperative seizure, conversion to GA and new neurological dysfunction) was explored using logistic meta-regression with fixed effect for anaesthesia technique [13]. Odds ratio (OR) and 95% confidence intervals [95%CIs] were determined and considered statistically significant when the 95%CI excluded 1. If studies included a high proportion of the same study-population, we considered only the largest study for the meta-analysis [14,15]. Analyses were performed using "R" version 3.0.2 [16]; for meta-analysis the *meta* package was used.

#### Risk of bias across studies

Publication bias was not assessed in this systematic review. Selective reporting bias was assessed with the above-mentioned risk of bias tools.

#### Additional analyses

Additional analyses were not pre-specified, but performed according to the request of the reviewers. Meta-analysis and meta-regression were performed for one composite outcome, comprising the life-threatening events AC failure, mortality and intraoperative seizures. Furthermore, a sensitivity analysis, by looking only at prospective studies, was conducted for the five outcomes, which were included in the meta-analyses. The relationship of anaesthesia technique (MAC/ SAS) as one potential source of heterogeneity and the five above-described outcomes (AC failure, intraoperative seizure, conversion to GA, new neurological dysfunction and the composite outcome) of prospective studies was explored using logistic meta-regression.

## Results

### Study selection

**Fig 1:**
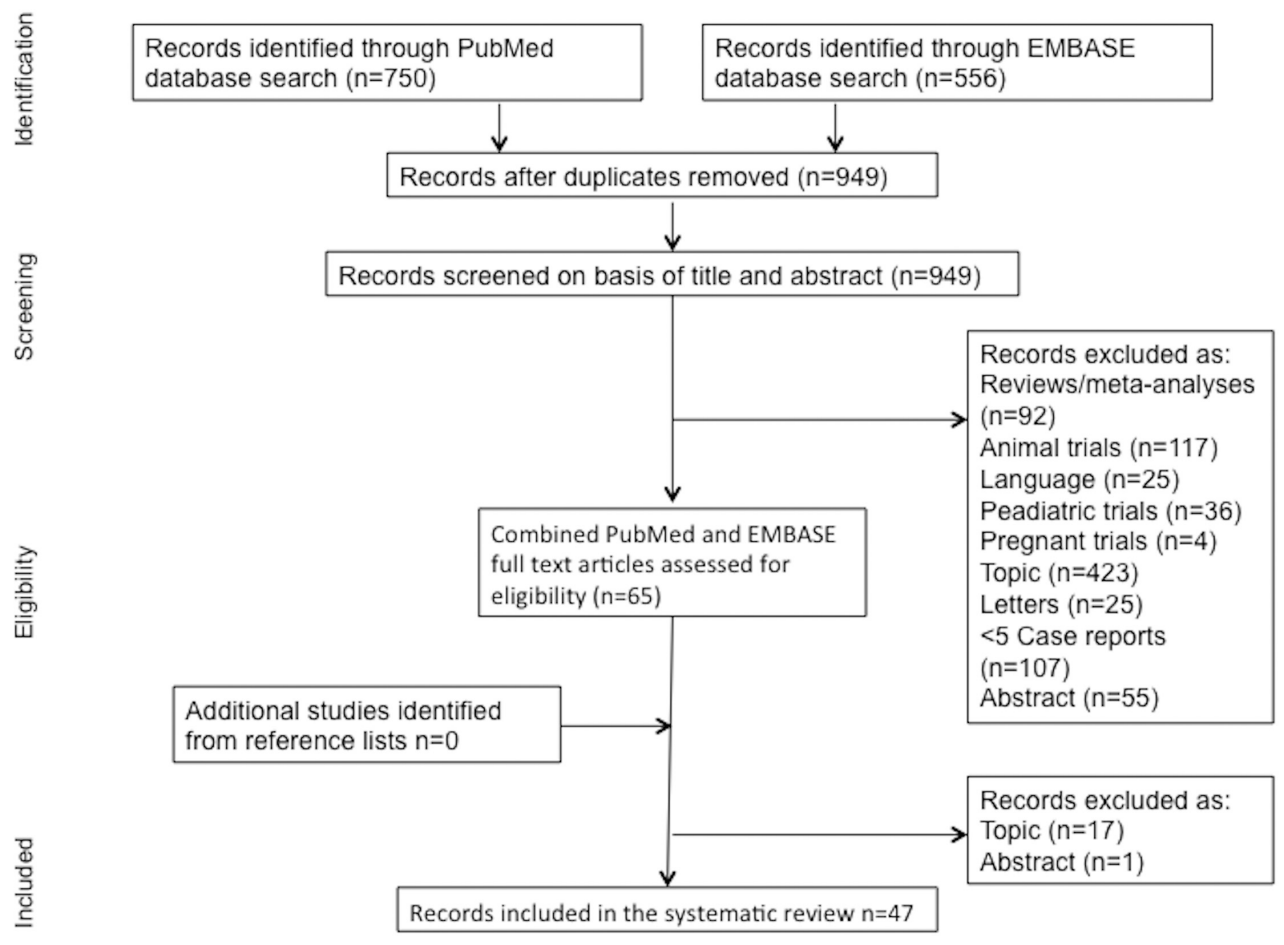
Study flow diagram.

Our search strategy in EMBASE and PubMed initially revealed 1303 publications. We did not identify any additional studies by screening the reference lists. The detailed screening, eligibility assessment and inclusion process is shown in Fig 1. We included a total of forty-seven studies [10,17–62] in our SR. One author was personally contacted, and provided us more information about their used anaesthesia technique [10].

### Study characteristics

Data of the study characteristics are shown in Table 1. A total of fourteen case series [10,17,19,20,23,28,39,41,44,47,51,53,54,60] thirteen prospective studies [18,21,22,25–27,30,33,35,38,52,55,61], seventeen retrospective studies [24,29,31,34,37,40,42,43,45,46,48–50,57–59,62], two RCTs [32,56], and one pseudo-RCT [36] comprising 5945 AC procedures in 5931 patients were analysed (Table 1). Of note, during the data extraction process it appeared that nine studies [20,22,27,31,42–46] partially reported on the same patient population. This refers to the study of Grossman et al. [31] and both studies of Nossek et al. [42,43], two publications of Ouyang et al. [45,46] the publications of Boetto and Deras et al. [22,27] and at least the studies of Andersen and Olsen et al. [20,44]. After complete data extraction we discussed with all authors how to deal with these partial duplicates. Consensus was found to retain all publications for the study descriptions, as they have all reported some different outcomes in these patients, which could provide additional useful information and the patient population was not absolutely the same [63]. In contrast, for a reasonable meta-analysis only the largest study of the duplicate studies was chosen, as the complete elimination of duplicate studies would bias the meta-analysis in its entirety [14,15].

**Table 1:** Study characteristics.

Study	Study design	Recruitment period	Sample Size of AC patients	Different AC groups?	Aim /endpoint	Main findings
Abdou 2010 [17]	CS (prospective, 1 centre)	NK	28	No	To evaluate the clinical efficiency of a mixture of ketamine and propofol called "ketofol"-based sedation procedure for AC.	Conscious sedation during AC using "ketofol" infusion mixture in 1:1 ratio was safe and efficient with minor haemodynamic and respiratory events and rapid smooth recovery profile.
Ali 2009 [18]	PS (1 centre)	1/2007-11/2008	20	No	To compare AC technique with GA for excision of low-grade glioma involving eloquent cortex.	AC is a relatively safe procedure with minimal morbidity and does not require a sophisticated technology. Compared to GA, tumour excision in eloquent areas is safer with AC.
Amorim 2008 [19]	CS (1 centre)	2001–2004	12	No	To assess the safety and effectiveness of AC in regard to the resection size and postoperative neurological outcome.	Gross total resection was achieved in 66% and only one patient experienced permanent neurological dysfunction postoperatively.
Andersen 2010 [20]	CS (1 centre)	5/2004–3/2009	44	No	To retrospectively evaluate the safety of AC in the first AC cases of one institution.	AC was well tolerated and implied several advantages.
Beez 2013 [21]	PS (5 centres)	2010–2011	105	Yes (multi-centre trial)	To evaluate pain and discomfort during the awake phase of AC.	AC was well tolerated with low pain and anxiety levels. Female and younger patients experience higher anxiety levels. Discomfort resulted from head fixation or positioning on the operating table.
Bilotta 2014 [10]	CS (prospective, 1 centre)	2013	20	No	To describe the experience using a language testing work-up for patients with or at risk for language disturbances undergoing AC.	Broca´s area was identified in 15 patients, in all cases by counting arrest and in 12 cases by naming arrest. This approach allows a systematic evaluation of language function status during AC, even when a neuropsychologist or speech therapist is not involved in the operation crew.
Boetto 2015 [22]	PS (1 centre)	1/2009-1/2014	374	No	To analyse the incidence, risk factors and consequences of intraoperative seizures during AC without ECoG.	AC was performed safely and reliable without ECoG. There was a low rate of intraoperative seizures, even in patients with intractable seizure history.
Cai 2013 [23]	CS (1 centre)	11/2008-08/2011	17	No	To describe the experience with an oesophageal naso-pharyngeal tube in asleep-awake-asleep anaesthesia.	In all 17 patients the naso-pharyngeal tube was easy to place and well tolerated. During the awake period no excess sedation, lack of cooperation, or hypoxia was recorded.
Chacko 2013 [24]	RS (1 centre)	2002–2010	67	No	To describe the experience in 67 consecutive ACs for the excision of tumours located in or around eloquent areas, regarding intraoperative and postoperative deficits.	AC with electro cortical stimulation for eloquent area tumours enables removal of a large tumour volume with good functional outcome. There were no anaesthesiological complications and intraoperative seizures were successfully ceased with cold saline irrigation and anticonvulsants.
Chaki 2014 [25]	PS (1 centre)	01/2011-06/2013	53	No	To elucidate the efficacy and safety of a mixture of lidocaine and ropivacaine for scalp nerve block.	Mixture of lidocaine and ropivacaine for scalp nerve blocks in AC is safe and effective. Despite large amounts of the two administered local anaesthetics, the blood level remained under half of the known toxic level for both of them.
Conte 2013 [26]	PS (1 centre)	04/2009-05/2010	27	No	To assess if BIS monitoring shortens patient´s awakening and predicts recovery of consciousness in order to establish reliable brain mapping.	Higher BIS values are associated with shorter awakening times during asleep-awake craniotomies. The return of BIS values to pre-induction values was associated with patient´s capability to perform intraoperative language testing.
Deras 2012 [27]	PS (1 centre)	01/2008-11/2010	140	No	To assess the efficacy (feasibility and timing of the awake phase) and safety (occurrence of adverse events) of an SAS protocol with controlled ventilation during the asleep phase.	The SAS protocol was feasible and relatively safe, despite one case of pulmonary aspiration (without sequel) and 31.8% of difficult oral intubation respectively 14.8% for laryngeal mask insertion.
Garavaglia 2014 [28]	CS (prospective, 1 centre)	03-12/2012	10	No	To assess the anaesthetic technique based on SNB and Dexmedetomidine without airway manipulation in high risk patients.	All patients underwent successful AC, intraoperative mapping, and tumour resection with adequate sedation. Dexmedetomidine in combination with RSNB enables an effective and safe anaesthetic technique for AC.
Gonen 2014 [29]	RS (1 centre)	01/2010-05/2012	137	4 groups, retrospectively built depending on tumour location	To evaluate the association between tumour localization (particularly SMA) and IDH1 mutation status, and the occurrence of intraoperative seizures during AC.	Intraoperative seizures were significantly more frequent in patients with tumours located in the SMA region and a history of seizure.
Grossman 2007 [30]	PS (1 centre)	NK	40	No	To evaluate the effect of wound infiltration and a single dose of metamizole against postoperative pain after AC.	RSNB and local infiltrations in combination with metamizole may provide an effective pain control in AC patients.
Grossman 2013 [31]	RS (1 centre)	2003–2010	424	2 groups retrospectively built (334 young and 90 elderly >65years)	To compare surgical outcome between younger and elderly patients undergoing AC.	There was no difference between the groups regarding rate of mortality, or complications. However age was associated with increased length of stay. Maximal extent of HGG tumour resection was associated with prolonged survival rate.
Gupta 2007 [32]	RCT (1 centre)	1/2001-5/2003	26	1 AC group	To compare the efficacy of AC versus GA for patients with tumours in eloquent, in regard to new neurological dysfunctions and the extent of tumour resection.	Except for the surgery time, they did not find any significant statistical difference between the groups.
Hansen 2013 [33]	PS (1 centre)	05/2006-03/2012	50 procedures in 47 patients	No	To report a novel approach of AC based on cranial nerve block, permanent presence of a contact person, psychological guidance and therapeutic communication.	No patient required sedation, only two-thirds of the patients requested remifentanil with a mean of 96 µg before the end of tumour resection. Hemodynamic reactions were mainly seen during nerve blockades and neurological testing. This approach was considered as “awake-awake-awake-technique”
Hervey-Jumper 2015 [34]	RS (1 centre)	1997–2014	611	No	To analyse a single surgeon’s experience and the evolving methodology of awake language and sensorimotor mapping for glioma surgery.	AC can be safely performed with few complications and a low failure rate, regardless of ASA, Mallampati score, BMI, smoking, psychiatric history, seizure history, or tumour mass effect. Incidence of seizures was associated with preoperative seizure history and tumour location. There was no statistical difference between the used sedation technique and intraoperative seizures, LMA use, kind of tumour, BMI or AC failures.
Ilmberger 2008 [35]	PS (1 centre)	1991–2005	153 procedures in 149 patients	No	To evaluate pre- and postoperative language function using a standardised neurolinguistic test battery, after AC for tumours in eloquent areas.	AC is a safe and economic, well tolerated procedure. Every attempt should be undertaken to preserve language-relevant areas intraoperatively. New postoperative deficits were resolved in the majority of patients. Patients with suboptimal preoperative naming capacities are at higher risk for early postoperative language impairment.
Jadavji-Mithani 2015 [36]	Pseudo-RCT (1 centre)	05-08/2012 and 05-08/2013	29	2 groups (major key music and minor key music)	To assess if music is beneficial for AC patients.	Overall, listening to music selections was beneficial for the patients. Adverse events were independent of the kind of music.
Kim 2009 [37]	RS (1 centre)	1/1993-372006	309 procedures in 289 patients	No	To analyse the correlation of intraoperative cortical mapping and postoperative neurological outcomes.	Negative mapping of eloquent areas enables surgical resection with a low rate of neurological deficits. Tumour proximity to functional cortex bears an increased risk for postoperative neurological deficits.
Li 2015 [38]	PS (1 centre)	01/2003-01/2012	91	No	To investigate the method and significance of direct electrical stimulation (DES) to the brain mapping of language functions during glioma surgery in Chinese patients	DES was found to be a reliable non-invasive method for cerebral functional area positioning and maximal safe resection of gliomas in Chinese patients.
Lobo 2007 [39]	CS (1 centre)	NK	8	No	To describe an SAS technique with propofol and remifentanil infusion, pharmacokinetic simulation to predict the effect-site concentrations and to modulate the infusion rates of both drugs, and bispectral index (BIS) monitoring.	A significant correlation was found between BIS and predicted effect-site concentrations of propofol (r^2^ = 0.547, P<0.001) and remifentanil (r^2^ = 0.533, P<0.001). Intraoperative awakening was very fast (3 minutes).
Low 2007 [40]	RS (1 centre)	7/2003-8/2006	20	No	To examine the safety and effectiveness of AC under local anaesthesia and MAC sedation for resection of tumours involving eloquent cortex.	MAC sedation in combination with frameless computer stereotactic guidance is a safe technique, which enables maximal resection of lesions in close relationship to eloquent cortex and has a low risk of neurological deficit.
McNicholas 2014 [41]	CS (2 centres)	1 year (not further specified)	42 (Rome n = 28, Chicago n = 14)	No	To describe transient postoperative facial nerve palsy as a complication of auriculotemporal nerve blockade in AC.	Seven out of 42 patients developed transient postoperative facial nerve palsy. The technique may need to be refined to avoid such complications.
Nossek 2013 [42]	RS (1 centre)	2003–2010	424	2 groups were retrospectively built. (AC failure n = 397 patients vs. n = 27 not failure patients)	To assess the prevalence of AC failure (general anaesthesia required or adequate mapping failed).	27 (6.4%) AC failures occurred with multiple reasons: lack of communication (4.2%) and intraoperative seizures (2.1%). Preoperative dysphasia and treatment with phenytoin were related to failure. The majority of AC failures were preventable by adequate patient selection and by avoiding side effects of drugs administered during intervention.
Nossek 2013 [43]	RS (1 centre)	2003–2011	477	2 groups were retrospectively built. (seizure n = 60 + non-seizure n = 417)	To analyse the incidence, risk factors, and consequences of seizures during asleep-awake-asleep AC.	60 patients (12.6%) of 477 patients with complete records experienced intraoperative seizures in which 2.3% failed AC procedure. Seizures are more frequent in younger patients, in patients with frontal lobe involvement, and in patients with a history of seizures. Seizures are associated with a short-term motor deterioration and a longer hospitalisation. The perioperative team should be prepared to treat intraoperative seizures.
Olsen 2008 [44]	CS (1 centre)	5/2004-2/2006	25	No	To present a new ‘asleep–awake’ technique for tumour resection.	The presented method was well tolerated by the patients and allowed modification of the surgery according to the live intraoperative mapping results. Omitting a second asleep phase at the end of surgery seems to be more advantageous compared to the SAS technique.
Ouyang 2013 [45]	RS (1 centre)	01/2005-12/2010	386	2 groups retrospectively built. (midline-shift n = 103 + no midline-shift n = 283)	To identify if patients with midline shift, and more cerebral oedema would suffer from a higher incidence of PONV.	There was no correlation between midline shift and postoperative nausea or pain in AC.
Ouyang 2013 [46]	RS (1 centre)	01/2005-12/2010	415	2 groups were retrospectively built. (benign tumour n = 115 + malignant tumour n = 300)	To compare the incidence of postoperative nausea between benign and malignant brain tumours.	There was no difference in the incidence of nausea between benign and malignant brain tumours, but patients with benign tumours showed a higher pain score postoperatively.
Pereira 2008 [47]	CS (prospective, 1 centre)	1998–2007	79	2 groups (Group A without multidisciplinary team 1998-7/2004 n = 33, group B with multidisciplinary team 8/2004-2008 n = 46)	To evaluate the safety and efficacy of fully AC for the resection of primary supratentorial brain tumours near or in eloquent brain areas. Furthermore, to assess the impact of previous surgery and treatment modalities on the outcome.	AC is a safe and effective procedure and in a multidisciplinary context is associated with greater clinical and physiological monitoring. The outcome was not influenced by surgical history of AC.
Peruzzi 2011 [48]	RS (2 centres)	1/2006-12/2008	22 procedures in 20 patients	1 AC group	To compare the hospital length of stay, hospital cost, perioperative morbidity, and postoperative outcome between patients undergoing awake glioma surgery vs. surgery under GA.	There were no significant differences in the patient outcomes, but the hospital length of stay and hospital costs were significantly reduced in the AC group.
Pinsker 2007 [49]	RS (1 centre)	1/1998-12/2002	55 procedures in 52 patients	No	To analyse the safety and maximal extension of tumour resection with AC in the eloquent brain area.	AC enables a more radical resection of tumours in eloquent brain areas, otherwise considered as inoperable.
Rajan 2013 [50]	RS (1 centre)	2007–2010	101	No	To assess if AC (asleep-awake-asleep) with dexmedetomidine/ propofol/ fentanyl has acceptable perioperative outcomes compared to general anaesthesia.	Temporary episodes of desaturation and hypercapnia occurred more often in the AC group. Blood pressure was lower in the AC group during application of head clamp pins and emergence and the AC group required less vasopressors intraoperatively. AC provides adequate sedation, analgesia and a smooth wake-up during the period of neurological monitoring with stable haemodynamic and acceptable respiratory parameters compared to general anaesthesia. AC group showed less PONV and pain postoperatively.
Rughani 2011 [51]	CS (1 centre)	01/2007-07/2009	25	No	Description of a new anaesthesiological protocol and patient outcomes for the first patients undergoing AC surgery in this institution.	Implementation of the new anaesthesiological approach was successful, with a low operative morbidity and rate of anaesthesia complications, short surgery time, and well tolerance by the patients.
Sacko 2010 [52]	PS (1 centre)	01/2002-12/2007	214	No	To assess the safety and effectiveness of AC in comparison to GA for lesions close to the eloquent cortex.	AC patients showed a significantly better neurological outcome, faster discharge times and an uneventful surgery.
Sanus 2015 [53]	CS (1 centre)	2010–2013	25	No	The safety and effectiveness of AC in 25 patients should be described.	AC in selected patients is an effective, safe and practical procedure, which is accompanied with a short hospital and ICU length of stay.
See 2007 [54]	CS (1 centre)	7/2004-6/2006	17	No	To analyse the individual anaesthetic management, intraoperative complications and postoperative outcome of patients undergoing AC.	AC was well tolerated and showed a low rate of complications, with the benefit of maximal tumour excision and a potentially better patient outcome.
Serletis 2007 [55]	PS (1 centre)	1/1991-7/2006	511	2 groups (eloquent cortex AC n = 511, non-eloquent cortex AC n = 99)	To elucidate the outcomes and potential advantages associated with AC for supratentorial tumour resection, treated by one neurosurgeon.	AC is safe, practical, and effective during resection of supratentorial lesions of diverse pathological range and location.
Shen 2013 [56]	RCT (1 centre)	01-11/2012	30	2 groups (propofol vs. dexmedetomidine)	To compare the efficacy and safety of dexmedetomidine versus propofol for conscious sedation in AC and to determine the arousal time until awake phase after asleep phase.	Arousal time was longer in the propofol group. Surgeon´s satisfaction was higher in the dexmedetomidine group. There was no difference in patients´ satisfaction, adverse outcomes and quality of revival. Both anaesthetics can be effectively and safely used for conscious sedation in AC.
Shinoura 2013 [57]	RS (1 centre)	2003–2013	102	No	To analyse associated factors for worsened paresis after AC for brain lesions located within or near the primary motor area.	Preoperative motor deficits, closeness to the motor area, partial resection, AC failure and intraoperative complications are associated risk factors for postoperative worsening of paresis.
Sinha 2007 [58]	RS (1 centre)	until 2005	42	2 groups (BIS n = 16, no BIS n = 26)	To evaluate the AC procedure in regard to complications during surgery.	With the use of advanced monitoring (BIS) and newer anaesthetics, AC was a relatively safe procedure with an acceptable rate of complications.
Sokhal 2015 [59]	RS (1 centre)	2001–2010	54	No	To analyse the anaesthetic management and perioperative complications in patients undergoing AC.	Appropriate patient selection and careful anaesthesia management are the keys to the success of AC. ‘Conscious sedation’ was performed successfully with fentanyl, propofol and dexmedetomidine. Patients treated with propofol showed fewer incidences of intraoperative seizures.
Souter 2007 [60]	CS (1 centre)	NK	6	No	To describe the experience with dexmedetomidine as the principle sedative agent on functional cortical mapping and ECoG recording during AC for excision of epileptogenic foci.	Dexmedetomidine as a single sedative was successfully used for AC including motor mapping, when coupled with RSNB and relatively small doses of fentanyl.
Wrede 2011 [61]	PS (1 centre)	4 years	48 procedures in 46 patients	1 AC group	To objectively assess the patients`experience with AC compared to GA for brain tumours by using a formal questionnaire.	A good patient acceptance of AC procedures for tumours in eloquent regions could be verified in this study.
Zhang 2008 [62]	RS (1 centre)	3/2005-5/2006	30	3 groups (1. positive mapping, 2. negative mapping, 3. aborted mapping)	To evaluate surgical resection of gliomas in eloquent brain regions with intraoperative cortical stimulation mapping under AC.	AC with cortical mapping is an accurate and safe approach to identify language cortex and enables extensive tumour excision while preserving normal language function and minimizing the risk of postoperative language deficits.

AC, awake craniotomy; BIS, bispectral index; CS, case study; ECoG, electrocorticography GA, general anaesthesia; IDH1, isocitrate dehydrogenase 1; n =, specified number of patients; PONV, postoperative nausea and vomiting; PS, prospective observational study; RA, regional anaesthesia; RCT, randomised controlled trial; RS, retrospective study; RSNB, regional scalp nerve block; SMA, supplementary motor area; TIVA, total intravenous anaesthesia.

Anaesthesia characteristics, including the kind of anaesthesia technique, used drugs and dosages and the description of the patient’s airway are presented in Tables 2 and 3. The patient characteristics are summarised in the S1 Table. Intraoperative characteristics and adverse events are shown in Table 4 and the patient outcomes in Table 5.

**Table 2:** Anaesthesia characteristics part 1.

Study	Anaesthesia technique	Premedication/ additional medication	Local anaesthesia (Pins and dura)	RSNB	Drugs used for RSNB
Abdou 2010 [17]	MAC	Midazolam 15 μg kg^-1^, ondansetron 8 mg, dexamethasone 8 mg, phenytoin 250 mg and mannitol 0.5g kg^-1^ i.v.	Yes	NK	NK
Ali 2009 [18]	MAC	Clonidine 4 μg kg^-1^, ranitidine 50 mg and metoclopramide 10 mg, dexamethasone 8 mg, phenytoin 5 mg kg^-1^, diclofenac, and acetaminophen 1 g i.v. half an hour before surgery.	Yes	Yes	Mixture of bupivacaine 0.25% and lidocaine 1% with 1:200,000 epinephrine (2–3 ml at each infiltration site).
Amorim 2008 [19]	MAC	NK	Yes	Yes	Lidocaine 0.5%, bupivacaine 0.25% with epinephrine 1:200,000
Andersen 2010 [20]	SA	Midazolam (n = 2 patients), mannitol (n = 37)	Yes	Yes	NK
Beez 2013 [21]	97 patients SAS, 8 patients SA	NK	Yes	Yes	NK
Bilotta 2014 [10]	MAC	1–1.5 mg midazolam	Yes	Yes	Ropivacaine 0.75% injected on 4 sites in each side of the head (8 injections in total): 2.5 to 5 ml. The dose depends on the site and on the body weight of the patients. (In total 20-40ml ropivacaine)
Boetto 2015 [22]	SAS	4mg ondansetron, 400mg cimetidine, 1g acetaminophen. Anticonvulsant drugs were continued in patients with seizure history.	Yes	Yes	20 ml lidocaine 2% with epinephrine
Cai 2013 [23]	SAS	NK	NK	NK	NK
Chacko 2013 [24]	MAC	NK	Yes	Yes	A mixture of bupivacaine (1–1.5mg kg^-1^) and lidocaine (3–4 mg kg^-1^) with epinephrine concentration of 5μg ml^-1^
Chaki 2014 [25]	SAS	Betamethasone (4 mg) and famotidine (20 mg) as PONV prophylaxis	Yes	Yes	Lidocaine 2% and ropivacaine 0.75% with epinephrine at 1:200,000 (5μg ml^-1^)
Conte 2013 [26]	SA	Midazolam 0.07–0.08 mg kg^-1^ i.m. and atropine 0.5 mg i.m. 30–60 min. before surgery. Antiemetic: metoclopramide chloridrate 10 mg i.v. or dolasetron 5 mg i.v., anticonvulsant medication in all patients	Yes	Yes	Mixture of ropivacaine 1%, mepivacaine 1%, epinephrine 1:200,000, and lidocaine 2%.
Deras 2012 [27]	SAS	4mg ondansetron, 400mg cimetidine, 1g acetaminophen	Yes	NK	NA
Garavaglia 2014 [28]	MAC	4mg ondansetron i.v.	Yes	Yes	0,375% bupivacaine without epinephrine (2-3ml at each injection site)
Gonen 2014 [29]	MAC	Midazolam 1-2mg and 50–100μg fentanyl	Yes	Yes	NK
Grossman 2007 [30]	MAC	Clonidine 2–3 μg kg^-1^ one hour before surgery. At the end of surgery: Additional lidocaine 2% and epinephrine 1:200,000 were infiltrated to the scalp and dura mater and the patients received metamizole 1g intramuscularly/ respectively diclofenac 75 mg.	Yes	Yes	Bupivacaine 0.5% and lidocaine 2% in a 1:1 mixture.
Grossman 2013 [31]	MAC	Midazolam 1-2mg and 50-100g fentanyl	Yes	Yes	NK
Gupta 2007 [32]	MAC	No	Yes	Yes	Bupivacaine 0.5% with epinephrine 1:200,000 (2.5ml at each injection site), maximum 225 mg bupivacaine
Hansen 2013 [33]	AAA	8 mg dexamethasone	Yes	Yes	28 ml of ropivacaine 0.75% with epinephrine 1:200,000
Hervey-Jumper 2015 [34]	MAC	50μg fentanyl, 1-2mg midazolam, antiemetic drugs: ondansetron and scopolamine	Yes	Yes	1:1 mixture of 1% lidocaine with 1:100,000 epinephrine, 0.5% bupivacaine, plus 4.5 ml of 8.4% sodium bicarbonate
Ilmberger 2008 [35]	MAC	750 mg phenytoin	NK	Yes	60–80 ml bupivacaine 0.25%
Jadavji-Mithani 2015 [36]	MAC	Midazolam (dosage NK) only in 14 patients, who received propofol + remifentanil anaesthesia	Yes	No	NA
Kim 2009 [37]	SAS	4 mg ondansetron, 20 mg famotidine, and 10 mg metoclopramide preoperative.	Yes	Yes	40 ml ropivacaine 0.5% with epinephrine 1:200,000
Li 2015 [38]	SAS	NK	Yes	Yes	Bupivacaine or ropivacaine (dosage NK)
Lobo 2007 [39]	SAS	Midazolam 2.2 ± 0.3mg i.v. Dexamethasone 10 mg and ondansetron 4mg i.v. were given before incision. Phenytoin 250 to 500 mg i.v. during surgery	NK	Yes	Up to 40 ml ropivacaine 0.75% with epinephrine 1:200,000
Low 2007 [40]	MAC	Intravenous mannitol, dexamethasone, antibiotics and anticonvulsants were administered prior to skin incision.	Yes	Yes	Bupivicaine 0.5% and epinephrine (1:200,000)
McNicholas 2014 [41]	MAC	NK	Yes	Yes	Rome: n = 28, 40ml ropivacaine 0,75%, Chicago: n = 1, 20ml bupivacaine 0.25%with epinephrine 1:200,000, the others, n = 13, 6 ml of 1% tetracaine and 30 ml lidocaine 1% with epinephrine 1:100,000
Nossek 2013 [42]	MAC	Anticonvulsant medication in all patients, midazolam 1-2mg and 50-100g fentanyl	Yes	Yes	NK
Nossek 2013 [43]	MAC	Anticonvulsant medication in all patients, midazolam 1-2mg and 50-100g fentanyl	Yes	Yes	NK
Olsen 2008 [44]	SA	Midazolam n = 4. Paracetamol 1-2mg i.v., dehydrobenzperidol 0.6 mg, ondansetron 4 mg, dexamethasone 8 mg, mannitol n = 22. Phenytoin loading dose n = 24	Yes	Yes	15-20ml bupivacaine 5mg ml^-1^ + 5μg ml^-1^ epinephrine
Ouyang 2013 [45]	SAS	Dexamethasone 10–20 mg i.v., mannitol 1–2 g kg^-1^ intraoperative, ondansetron 4mg and/ or metoclopramide 10mg	NK (local anaesthesia mentioned, but not specified)	NK (local anaesthesia mentioned, but not specified)	NK
Ouyang 2013 [46]	SAS	Dexamethasone 10–20 mg i.v., mannitol 1–2 g kg^-1^ intraoperative, ondansetron 4mg and/ or metoclopramide 10mg	NK (local anaesthesia mentioned, but not specified)	NK (local anaesthesia mentioned, but not specified)	NK
Pereira 2008 [47]	MAC	Additional naloxone in some patients for opioid revision before mapping.	Yes	Yes	Bupivacaine 0.07% and epinephrine 1:800,000 (whole hemi cranium)
Peruzzi 2011 [48]	MAC	NK	Yes	No	NA
Pinsker 2007 [49]	MAC	Antiepileptic drug.	NK	Yes	0.375% bupivacaine
Rajan 2013 [50]	SAS	NK	Yes	No	NA
Rughani 2011 [51]	SAS	Midazolam 1-2mg i.v. and 50–200μg fentanyl, 10 min. before entering surgery room; 10 mg dexamethasone, 4-8mg ondansetron i.v.; mannitol 12.5 to 100g only if brain swelling; phenytoin 18mg kg^-1^ for each patient with additional 500mg phenytoin to already treated patients.	Yes	Yes	40ml 0.25% bupivacaine
Sacko 2010 [52]	MAC	Levetiracetam, 500 mg, methylprednisolone 1 mg kg^-1^	Yes	Yes	Lidocaine 1% with epinephrine 1:100 000
Sanus 2015 [53]	SAS	Midazolam 30–50 μg kg^-1^ i.v., anticonvulsants and corticosteroids immediately before surgery	Yes	No	NA
See 2007 [54]	MAC	Midazolam (n = 5), anticonvulsant therapy and dexamethasone were continued perioperatively.	Yes	Yes	0.75% lidocaine (1:200,000 adrenaline) with or without 0.25% bupivacaine
Serletis 2007 [55]	MAC	Anticonvulsant and corticosteroid.	Yes	Yes	0.25% bupivacaine
Shen 2013 [56]	SAS	No midazolam	Yes	Yes	60ml ropivacaine 0.25% including local infiltration anaesthesia (pins and scalp)
Shinoura 2013 [57]	SAS	NK	Yes	Yes	Lidocaine 1% with epinephrine and 0.75% anapain
Sinha 2007 [58]	MAC	No midazolam. Clonidine 4 μg kg^-1^, ranitidine, atenolol 25mg and double the dose of anticonvulsants orally in the morning. Ondansetron 4mg before and at the end of surgery. Haloperidol 2.5-5mg i.v. at induction. Corticosteroids, anti-epileptic drugs and mannitol were applied additionally.	Yes	Yes	Bupivacaine 0.25% and lidocaine 1% with 1:200,000 epinephrine (2–5 ml at each site). Mean 34.3ml, range [28-66ml]
Sokhal 2015 [59]	MAC	No midazolam, preoperative application of corticosteroids (dosage NK) and mannitol at surgery start.	Yes	Yes	At each site, 3-5ml bupivacaine 0.25–0.5%
Souter 2007 [60]	SAS (n = 2), MAC (n = 4)	No midazolam.	Yes	Yes	35–40 ml lidocaine 1.0% with 1:200,000 epinephrine and bupivacaine 0.25%.
Wrede 2011 [61]	MAC	NK	Yes	No	NA
Zhang 2008 [62]	MAC	Only minimal preoperative sedation is described.	Yes	Yes	Ropivacaine 0.5%

AAA, awake-awake-awake technique; Anaesth., Anaesthesia; Ces, effect-site concentration; i.m., intra muscular; i.v., intravenous; LMA, laryngeal mask airway; min., minutes; n =, specified number of patients; NA, not applicable; NK, Not known as not reported; PONV, postoperative nausea and vomiting; RSNB, Regional selective scalp nerve block; SA, asleep-awake technique; SAS, asleep-awake-asleep technique; TCI, Target controlled infusion; TIVA, total intravenous anaesthesia.

**Table 3:** Anaesthesia characteristics part 2.

Study	SA(S) Management	Dosage SA(S)	MAC /AAA Management	Awake phase	End of surgery	Use of muscle relaxants	Anaesth. depth control	Airway
Abdou 2010 [17]	NA	NA	Propofol 0.5 mg kg^-1^ h^-1^ and ketamine 0.5 mg kg^-1^ h^-1^ infusion mixture in 1:1 ratio in one syringe, thereafter adapted to the OAA/S score (aim level 3)	No medication	Resumed propofol/ ketamine mixture, and additional fentanyl 1–2μg kg^-1^ for postoperative analgesia	No	Only clinical with the (OAA/S) score	Nasal cannula (4 l min^-1^), (spontaneous breathing)
Ali 2009 [18]	NA	NA	1. Before RSNB: bolus propofol 50–100 mg and fentanyl 50μg. 2.Continous propofol 1–2 mg kg^-1^ h^-1^ and fentanyl 0.5 mg kg^-1^ h^-1^.	Continued conscious sedation	Continued conscious sedation	No	No	n = 15 nasal cannula (2–4 l min^-1^), n = 5 oropharyngeal airway; (spontaneous breathing)
Amorim 2008 [19]	NA	NA	Midazolam, fentanyl, propofol n = 6; dexmedetomidine 3 mg kg^-1^ h^-1^ (over 20 min.), followed by 0.5 mg kg^-1^ h^-1^ n = 6	NK	NK	No	No	Spontaneous breathing
Andersen 2010 [20]	TIVA (propofol + remifentanil)	NK	NA	Remifentanil n = 37, mean 0.03 [0–0.08] μg kg^-1^ min^-1^	Nothing	No	No	LMA (controlled ventilation), endotracheal tube in one AC patient
Beez 2013 [21]	TIVA (propofol + remifentanil)	NK	NA	No medication	TIVA (propofol + remifentanil) n = 97	No	No	LMA (controlled ventilation)
Bilotta 2014 [10]	NA	NA	Initial bolus of fentanyl 0.5–1μg kg^-1^, dexmedetomidine, midazolam and remifentanil (clinically adjusted to the patients`need).	No medication	Nothing	No	Only clinical by Richmond agitation sedation score (RASS aim 0/-2)	Oxygen via facemask. (spontaneous breathing)
Boetto 2015 [22]	TCI-TIVA (propofol + Remifentanil)	TCI: Initial: Propofol 6 μg ml^-1^ and remifentanil 6 ng ml^-1^. After dural incision: reduction of propofol to 3 μg ml^-1^ and remifentanil to 4 ng ml^-1^.	NA	No medication (LMA removal)	TCI-TIVA, propofol 6–12 μg ml^-1^ and remifentanil 6–12 ng ml^-1^	No	No	LMA (controlled ventilation) for the initial asleep phase, LMA or orotracheal tube with controlled ventilation for the second phase
Cai 2013 [23]	TCI-TIVA (propofol + Remifentanil)	TCI: Initial: Propofol 3–6 μg ml^-1^ and remifentanil 3–4 ng ml^-1^. After dural incision: reduction Ces of propofol to 1 μg ml^-1^ and remifentanil to 1 ng ml^-1^. Aim BIS 40–60.	NA	Only remifentanil 1 ng ml^-1^	NK	Rocuronium 0.6mg kg^-1^	BIS	Oesophageal naso-pharyngeal catheter (controlled ventilation)
Chacko 2013 [24]	NA	NA	Initial: 50 μg boluses of fentanyl and propofol or dexmedetomidine infusion. Thereafter propofol (1–2mg kg h^−1^)	No medication	NK (for 1 patient propofol is described)	No	No	2l min^-1^ oxygen via nasal cannula (spontaneous breathing)
Chaki 2014 [25]	TCI-Propofol	TCI: Initial 4.0μg ml^-1^ propofol. Thereafter reduction to 1.5–3.5μg ml^-1^	NA	No medication, if pain: 50 mg flurbiprofen i.v.	TCI-Propofol and reinsertion of LMA	Rocuronium 0.6mg kg^-1^	No	LMA (controlled ventilation)
Conte 2013 [26]	TIVA (propofol + remifentanil)	Initial: Propofol 2.0–2.5 mg kg^-1^ and remifentanil 0.025–0.1 μg kg^-1^ min^-1^. Thereafter: Propofol 5–10 mg kg^-1^ h^-1^ and remifentanil 0.05–0.2 μg kg^-1^ min^-1^.	NA	Reduced remifentanil 0.025–0.1 μg kg^-1^ min^-1^.	Reduced remifentanil 0.025–0.1 μg kg^-1^ min^-1^	No	BIS	LMA (controlled ventilation)
Deras 2012 [27]	TCI-TIVA (propofol + Remifentanil)	TCI: Initial: Propofol 6 μg ml^-1^ and remifentanil 6 ng ml^-1^. After dural incision: reduction of propofol to 3 μg ml^-1^ and remifentanil to 4 ng ml^-1^.	NA	No medication (LMA removal)	TCI-TIVA, propofol 6–12 μg ml^-1^ and remifentanil 6–12 ng ml^-1^	No	No	LMA (controlled ventilation) for the initial asleep phase, LMA or orotracheal tube with controlled ventilation for the second phase
Garavaglia 2014 [28]	NA	NA	Initial: dexmedetomidine 0.5–1μg kg^-1^ loading dose. Thereafter: 0.3–0.4 μg kg^-1^ h^-1^dexmedetomidine supplemented with 50–100μg fentanyl or 0.01–0.015μg kg^-1^min^-1^remifentanil and midazolam 1-4mg	No medication	Dexmedetomidine 0.2–1μg kg^-1^min^-1^ and 0.005–0.01μg kg^-1^min^-1^remifentanil	No	Only clinical by Richmond agitation sedation score (RASS aim 0/-2)	3l min^-1^ oxygen via facemask. (spontaneous breathing)
Gonen 2014 [29]	NA	NA	Remifentanil in low dosage and if necessary supplementation with propofol. (Exact dosage NK)	No medication	Remifentanil and supplementation with propofol. (Dosage NK)	No	No	3l min^-1^ oxygen via nasal cannula. (spontaneous breathing)
Grossman 2007 [30]	NA	NA	1. Propofol at an initial dose of 50 μg kg^-1^ min^-1^ and remifentanil 0.05 μg kg^-1^ min^-1^. 2. Remifentanil reduction to 0.01 μg kg^-1^ min^-1^ and propofol adjusted.	No medication	Propofol was resumed with 15 μg kg^-1^ min^-1^ and if needed additional remifentanil 0.01 μg kg^-1^ min^-1^ was applied (n = 18).	No	No	Nasal cannula (spontaneous breathing)
Grossman 2013 [31]	NA	NA	Remifentanil in low dosage and if necessary supplementation with propofol. (Exact dosage NK)	No medication	Remifentanil and supplementation with propofol. (Dosage NK)	No	No	3l min^-1^ oxygen via nasal cannula. (spontaneous breathing)
Gupta 2007 [32]	NA	NA	Initial: Fentanyl 2–3 μg kg^-1^ and propofol 2–2.5 mg kg^-1^. Thereafter: additional bolus of fentanyl 1 μg kg^-1^ (usually every 2h), and continuous propofol 50–100 μg kg^-1^ min^-1^.	Reduced dosage of propofol and fentanyl	As at the beginning	No	No	3l min^-1^ oxygen via nasal cannula. (spontaneous breathing)
Hansen 2013 [33]	NA	NA	Remifentanil was only required in 34 patients. Mean dosage 156±100 μg for the whole AC procedure.	Required remifentanil dosage during tumour resection 96±57 μg	Remifentanil was only required in 34 patients. Mean dosage 156±100 μg for the whole AC procedure.	No	BIS	Nasal cannula (spontaneous breathing)
Hervey-Jumper 2015 [34]	NA	NA.	TIVA (propofol up to 100 μg kg^-1^ min^-1^ + remifentanil 0.07–2.0 μg kg^-1^ hr^-1^) n = 327, dexmedetomidine up to 1 μg kg^-1^ min^-1^+ remifentanil n = 26, adjusted technique using all drugs n = 258	No medication (LMA removal), remifentanil continued in anxious patients.	TIVA or dexmedetomidine + remifentanil (reinsertion of LMA if indicated)	No	No	Nasal cannula (spontaneous breathing), additionally nasal trumpet if snoring. In high-risk patients n = 8 (high BMI, high tumour mass, high blood loss estimated) LMA.
Ilmberger 2008 [35]	NA	NA	Continuous propofol (0.5–1.2 mg kg-^1^) and remifentanil (0.05–0.01 μg kg^-1^ min^-1^)	NK	Resuming sedation like at the beginning	No	No	Nasal cannula (spontaneous breathing)
Jadavji-Mithani 2015 [36]	NA	NA	TIVA (propofol + remifentanil) n = 14, dexmedetomidine n = 15	No medication	NK	No	No	Nasal cannula or facemask (spontaneous breathing)
Kim 2009 [37]	TIVA (propofol + remifentanil)	Initial: Propofol 50–100 mg and remifentanil 0.1–0.2 μg kg^-1^ min^-1^. Thereafter: ≤ 3% desflurane and remifentanil 0.05–0.2 μg.	NA	LMA removal, if needed: remifentanil 0.02 μg kg^-1^ min^-1^	Dexmedetomidine 0.5–0.7 μg kg^-1^ h^-1^ and propofol 25–50 μg kg^-1^ min^-1^ and remifentanil 0.02–0.05 μg kg^-1^ min^-1^, LMA reinserted.	50 mg rocuronium (some patients)	No	LMA (controlled ventilation)
Li 2015 [38]	Propofol	NK	NA	No medication (LMA removed)	Propofol and LMA reinserted	No	No	LMA (controlled ventilation)
Lobo 2007 [39]	TCI-TIVA (propofol + Remifentanil)	Initial: 1% Propofol-TCI Schneider model 200ml h^-1^ and remifentanil-TCI, Minto model, 2.5ng ml^-1^, thereafter: 1 mg ml^-1^ of propofol and 2 ng ml^-1^ effect site concentration	NA	TCI-TIVA on low level (LMA removed)	TCI-remifentanil 2.5 ng ml^-1^and propofol bolus 10mg. LMA if needed 0.05 mg kg^-1^ morphine	No	BIS	LMA (controlled ventilation)
Low 2007 [40]	NA	NA	Combination of midazolam, propofol, fentanyl or remifentanil	Reduced dosage	Deep sedation	No	No	Oxygen via facemask (spontaneous breathing)
McNicholas 2014 [41]	NA	NA	Rom: (n = 28) Initial 1μg kg^-1^ fentanyl + propofol 0.5mg kg^-1^. Thereafter propofol 1.6–8.3 μg kg^-1^ min^-1^. Chicago: (n = 13) Initial remifentanil (exact dosage NK, but aim 8–12 breaths min^-1^ + propofol 10–25 μg kg^-1^ min^-1^, (n = 1) 2mg midazolam and 100μg fentanyl	No medication	NK	No	No	Oxygen via facemask (spontaneous breathing)
Nossek 2013 [42]	NA	NA	Remifentanil in low dosage and if necessary supplementation with propofol. (Exact dosage NK)	No medication	Remifentanil and supplementation with propofol. (Dosage NK)	No	No	3l min^-1^ oxygen via nasal cannula. (spontaneous breathing)
Nossek 2013 [43]	NA	NA	Remifentanil in low dosage and if necessary supplementation with propofol. (Exact dosage NK)	No medication	Remifentanil and supplementation with propofol. (Dosage NK)	No	No	3l min^-1^ oxygen via nasal cannula. (spontaneous breathing)
Olsen 2008 [44]	TIVA (propofol + remifentanil)	Initial: remifentanil 0.7 μg kg^-1^ min^-1^, bolus propofol (median 200mg) until loss of eyelid reflex, followed by continuous propofol 0.17 mg kg^-1^ min^-1^, thereafter 50% reduction of remifentanil and propofol	NA	Remifentanil 0.3 μg kg^-1^ min^-1^	Nothing	No	No	LMA (controlled ventilation), endotracheal tube in one AC patient
Ouyang 2013 [45]	TIVA (Propofol + remifentanil + fentanyl)	Initial: Propofol 1–2 mg kg^-1^, lidocaine (0.5–1.5 mg kg^-1^ and fentanyl 1–2 μg kg^-1^. Thereafter: Propofol 100–150 μg kg^-1^ min^-1^ and remifentanil 0.05–0.09 μg kg^-1^ min^-1^.	NA	No medication	TIVA (Propofol + remifentanil)	No	No	LMA or nasal trumpets (spontaneous breathing)
Ouyang 2013 [46]	TIVA (Propofol + remifentanil + fentanyl)	Initial: Propofol 1–2 mg kg^-1^, lidocaine (0.5–1.5 mg kg^-1^ and fentanyl 1–2 μg kg^-1^. Thereafter: Propofol 100–150 μg kg^-1^ min^-1^ and remifentanil 0.05–0.10 μg kg^-1^ min^-1^.	NA	No medication	TIVA (Propofol + remifentanil)	No	No	LMA or nasal trumpets (0,5–1,0 F_i_O_2_, spontaneous breathing), during awake phase only 0,21 F_i_O_2._
Pereira 2008 [47]	NA	NA	Group A (n = 33) 3/1998–2/200,2 bolus titration of propofol and remifentanil or fentanyl, plus midazolam. Group B (n = 46) after 2/2002, only fentanyl (50μg) boluses slowly until the minimum dose of 10 μg kg-1 in the first 1 h, followed by fentanyl 1 μg kg-1 every further hour (n = 43)	Until 2002 no medication, after 2002 adapted fentanyl boluses	After 2002 repeated boluses of fentanyl	No	No	Spontaneous breathing
Peruzzi 2011 [48]	NA	NA	Initial: dexmedetomidine 0.1–0.7μg kg^-1^ h^-1^ and if needed: 0.1mg kg^-1^ midazolam, thereafter bolus propofol until loss of consciousness, followed by a continuous application of propofol 40–120 μg kg^-1^ min^-1^ combined with dexmedetomidine 0.1–0.7μg kg^-1^ h^-1^. Sevoflurane 0.5–1% was added, to reduce propofol. BIS aim 50–60.	Only titrated dexmedetomidine infusion and fentanyl 12.5–25 μg if needed for pain	Additional propofol	No	OAA/S and BIS	Oxygen via nasal trumpet, connected to the ventilator (spontaneous breathing)
Pinsker 2007 [49]	NA	NA	Propofol (dosage NK)	No medication	Propofol if required	No	No	Oxygen via nasal cannula (spontaneous breathing)
Rajan 2013 [50]	Propofol + dexmedetomidine	Initial: Propofol 50–250 μg kg^-1^ min^-1^ and dexmedetomidine 1 μg kg^-1^ loading dose (in 10–15 min.). Thereafter Propofol 50–250 μg kg^-1^ min^-1^ and dexmedetomidine 0.4–0.7 μg kg^-1^ hr^-1^.	NA	Cessation propofol, reduction/ cessation of dexmedetomidine and 25–50μg fentanyl, if required for pain (fentanyl mean ± SD 169.8 μg ± 80.32μg)	NK	No	No	2-8l min^-1^ oxygen via nasal airway and nasal cannula. (spontaneous breathing)
Rughani 2011 [51]	Propofol + fentanyl + midazolam	Initial: propofol 0.1–0.3mg kg^-1^, then continuously 0.025–0.05 mg kg^-1^ min^-1^. Fentanyl 50–200μg and midazolam 1-2mg titrated as needed.	NA	No medication	Resuming propofol induction and continuous infusion, with fentanyl and midazolam as needed.	No	No	Spontaneous breathing, oral airway only described for 5 patients
Sacko 2010 [52]	NA	NA	Continuous propofol (1–3 mg kg^-1^ h^-1^) and fentanyl 1–3 μg kg^-1^ hr^-1^ or remifentanil 0.01–0.25 μg kg^-1^ hr^-1^	Cessation propofol only	Resuming propofol infusion	No	No	6l min^-1^ oxygen via face mask
Sanus 2015 [53]	Propofol + dexmedetomidine + remifentanil	Dexmedetomidine 0.02–0.5 μg kg^-1^ hr^-1^, propofol 30–180 μg kg^-1^ hr^-1^ and remifentanil 0.03–0.09 μg kg^-1^ hr^-1^ are used. BIS target 60–80.	NA	BIS target >80, no further information	NK	No	BIS	Nasopharyngeal airway (spontaneous breathing)
See 2007 [54]	NA	NA	Fentanyl bolus 25–50μg, remifentanil 0.005 to 0.02 3 μg kg^-1^ min^-1^, propofol (n = 15),	No medication	NK	No	No	Nasal cannula (spontaneous breathing)
Serletis 2007 [55]	NA	NA	Propofol, midazolam and fentanyl, exact dosage NK	NK	Propofol, midazolam and fentanyl, exact dosage NK	No	No	Oxygen via nasal cannula, (spontaneous breathing)
Shen 2013 [56]	TCI-TIVA (propofol + remifentanil) + dexmedetomidine	Initial in both groups: propofol-TCI Marsh model, Cp 4 μg ml^-1^, cis-atracurium 0.2 mg kg^-1^ and remifentanil-TCI, Minto model, 3ng ml^-1^ (Cp). Thereafter in both groups: remifentanil 2ng ml^-1^ (Cp) and cis-atracurium 0.1 mg kg^-1^ h^-1^ and aim RE 60–80. Propofol group: propofol 1–4 μg ml^-1^ (Cp). Dexmedetomidine group: propofol discontinued and dexmedetomidine loaded with 1μg kg^-1^, followed by 0.2–0.7 μg kg^-1^ h^-1^.	NA	Both groups: Aim RE >80. Remifentanil 0.5 ng ml^-1^ (Cp). Propofol group: propofol discontinued and normal saline (placebo) 5 ml h^-1^ was infused. Dexmedetomidine group: dexmedetomidine 0.2 μg kg^-1^ h^-1^.	Only re-induction of anaesthesia is mentioned.	Cis-atracurium 0.2 mg kg^-1^ and continuous infusion of 0.1 mg kg^-1^ h^-1^	RE	Endotracheal tube, controlled ventilation, FiO_2_ = 1.0.
Shinoura 2013 [57]	Propofol, dexmedetomidine, or remifentanil	NK	NA	Nothing	Propofol and replacement of LMA	NK	NK	LMA at the beginning and the end, ventilation mode NK
Sinha 2007 [58]	NA	NA	1.) Fentanyl 25–50 μg before application of RSNB 2.) Induction with propofol 1–2 mg kg^-1^, fentanyl 0.5–1.0 μg kg^-1^, and midazolam 1–2 mg i.v. 3.) Continuous fentanyl 0.5–2 μg kg^-1^ h^-1^ and propofol 1–5 mg kg^-1^ h^-^1. Aim BIS >60. 4.) Diclofenac 50–75 mg/ tramadol 50–100 mg if needed	NK	NK	No	BIS (n = 16)	Oxygen via nasal cannula 2–4 l min^-1^, continuous positive airway pressure was delivered through nasal trumpet in 1 patient
Sokhal 2015 [59]	NA	NA	1.) Fentanyl and propofol until 2010 (n = 44), titrated as bolus/ continuous (fentanyl 0.25–1.5 μg kg^-1^ h^-1^, propofol 25–200 μg kg^-1^ min^-1^. 2.) Since 2010: Dexmedetomidine solely (n = 6) 1 μg kg^-1^ loading dose, followed by 0.2–0.7 μg kg^-1^ h^-1^, 3.) along with titrated doses of fentanyl (n = 3), 4.) along with titrated doses of propofol and fentanyl (n = 1). Aim RE/ BIS: 60–80	Cessation of propofol, but continued fentanyl and dexmedetomidine	NK	No	RE/ BIS (n = 14)	Oxygen mask or nasal cannula (spontaneous breathing)
Souter 2007 [60]	SAS (n = 2) Propofol, fentanyl, dexmedetomidine	Induction with 3 mg kg^-1^ propofol, thereafter 100–200 μg kg^-1^ min^-1^ and fentanyl 25 μg boluses.	1. Only dexmedetomidine 01.5–0.7 μg kg^-1^ h^-1^ (n = 3), 2. Additional propofol 150–200 μg kg^-1^ min^-1^ for the beginning of surgery (n = 1). Fentanyl 25–50μg was additionally applied in 3 patients.	SAS (n = 2): cessation of propofol, removal of LMA and start of dexmedetomidine 0.1–0.3 μg kg^-1^ h^-1^, MAC (n = 4) continuous dexmedetomidine.	SAS (n = 2) reinduction of propofol and reinsertion of LMA.	No	Only clinical with the (OAA/S) score	SAS (n = 2): LMA; MAC (n = 4) oxygen 2–4 l min^-1^ nasal cannula (spontaneous breathing)
Wrede 2011 [61]	NA	NA	Combination of midazolam, dehydrobenzperidol and piritramide	NK	NK	No	No	NK
Zhang 2008 [62]	NA	NA	TIVA-TCI with propofol (Marsh’s Model), and sufentanil (Bovill’s model) or remifentanil (Minto’s model)	NK	NK	No	BIS	Nasopharyngeal airway (spontaneous breathing)

± SD, and standard deviation; Cp, target plasma concentration; n =, specified number of patients; NA, not applicable; NK, not known as not reported; OAA/S, observer Assessment of alertness/ sedation score; RE, response entropy index; SD, standard deviation; TCI, target-controlled infusion.

**Table 4:** Intraoperative characteristics and adverse events.

Study	Duration surgery in min., mean ± SD [range]	Duration awake phase in min., mean [range]/ ± SD	AC failure	Conversion into GA	Intraoperative seizures /history of seizures in these patients	Intraoperative hypoxia	intraoperative hypertension (>20%deviation from baseline)	Nausea and/or vomiting
Abdou 2010 [17]	168.8 ± 19.4; [150–215]	NK	NK	0	2/NK	1	2	2 (postoperative), 1 (intraoperative)
Ali 2009 [18]	173 ± 13	NK	0	0	2/NK	5	NK	1 (postoperative), 0 (intraoperative)
Amorim 2008 [19]	NK	NK	0	0	2 (dex group)/2	0	NK	NK
Andersen 2010 [20]	NK	166 [75–320]	3 (LMA leakage n = 1, respiratory insufficiency n = 1, intraoperative bleeding n = 1)	3 (LMA leakage n = 1, respiratory insufficiency n = 1, intraoperative bleeding n = 1)	12/NK	1	1	4/2
Beez 2013 [21]	NK	76 [20–137]	0	0	14/12	NK, but no anaesthesiological complication reported	NK, but no anaesthesiological complication reported	NK
Bilotta 2014 [10]	NK	NK	0	0	3	0	NK	NK
Boetto 2015 [22]	NK	NK	0	0	13/12	NK	NK	NK
Cai 2013 [23]	[450–780]	NK	0	0	0	0	NK	NK
Chacko 2013 [24]	NK	NK	1 (restlessness)	0	3/NK	0	NK	NK
Chaki 2014 [25]	NK	96 ± 45	1 (pain)	1 (pain)	0	0	0	NK
Conte 2013 [26]	median 403 [259–562]	NK	0	0	4/NK	NK	1	NK
Deras 2012 [27]	NK	98 ± 27	0	0	0/NK	0	0	3 (intraoperative)
Garavaglia 2014 [28]	median 210 [180–540]	NK	0	0	0	0	0	NK
Gonen 2014 [29]	NK	NK	0	0	28/NK	NK	NK	NK
Grossman 2007 [30]	202 ± 45	NK	NK	NK	NK	NK	5	0
Grossman 2013 [31]	NK	NK	NK	0	20 (18 young and 2 elderly)/NK	NK	NK	NK
Gupta 2007 [32]	196	NK	1 (brain bulge)	1 (brain bulge)	1/NK	NK	NK	NK
Hansen 2013 [33]	217±45 [105–295]	NK	1 (seizure)	1 (seizure)	8/NK	3	22 (>10% deviation)	1 (postoperative)
Hervey-Jumper 2015 [34]	NK	NK	3 (seizures)	0	20/19	NK	NK	NK
Ilmberger 2008 [35]	NK	NK	0	0	7/69	NK	NK	NK
Jadavji-Mithani 2015 [36]	NK	NK	0	0	2/NK	1	NK	2 (intraoperative)
Kim 2009 [37]	NK	NK	1 (agitation/ pain)	8 (agitation/ pain)	27/22	NK	NK	NK
Li 2015 [38]	NK	NK	0	NK	7/NK	NK	NK	NK
Lobo 2007 [39]	NK	NK	0	0	1/NK	1	0	NK
Low 2007 [40]	165 [85–275]	NK	0	0	NK	1	3	NK
McNicholas 2014 [41]	NK	NK	0	NK	NK	NK	NK	NK
Nossek 2013 [42]	NK	NK	27 (seizures n = 5, severe restlessness n = 8, acute brain oedema n = 1, severe dysphasia n = 11, somnolence n = 2).	9 (seizures n = 5, severe restlessness n = 3, acute brain oedema n = 1).	49 /NK	NK	NK	NK
Nossek 2013 [43]	NK	NK	37 (intractable seizures n = 11), dysphasia, restlessness, and somnolence n = 26).	7 (seizures)	60/37	NK	NK	NK
Olsen 2008 [44]	NK	165 [75–245]	2 (LMA leakage n = 1, intraoperative brain swelling n = 1)	2 (LMA leakage n = 1, intraoperative brain swelling n = 1)	4/NK	0	1	1/1
Ouyang 2013 [45]	Malignant group 211.6±63.6, benign group 213.9±75.8	NK	NK	NK	NK	NK	NK	124 (benign group n = 39, malignant group n = 85) postoperative
Ouyang 2013 [46]	Midline shift 201.3±54.1, no midline shift 242.7±87.4	NK	NK	NK	NK	NK	NK	113 (midline shift n = 84, no midline shift n = 29) postoperative
Pereira 2008 [47]	NK	NK	2 (Intubation group B > 8/2004)	2 (Intubation group B > 8/2004)	16 (n = 2 group A, <8/2004; n = 14 group B >8/2004)/ NK	2 (Group B >8/2004)	NK	NK
Peruzzi 2011 [48]	NK	NK	0	0	NK/4	NK	NK	NK
Pinsker 2007 [49]	NK	NK	0	0	0/NK	NK	NK	NK
Rajan 2013 [50]	NK	NK	1 (hypoxia SpO_2_ <90%)	1 (hypoxia SpO_2_ <90%)	2/NK	26	28 (need for antihypertensive medication)	5 (postoperative)
Rughani 2011 [51]	159, range [75–315]	NK	1 (respiratory insufficiency)	1 (respiratory insufficiency)	3/1	NK	NK	0
Sacko 2010 [52]	135	NK	0	0	14/NK	0	0	NK
Sanus 2015 [53]	NK	NK	0	0	0	0	0	NK
See 2007 [54]	median 240 [120–420]	NK	0	0	0	3	4	1 (intraoperative), 2 (postoperative)
Serletis 2007 [55]	NK	NK	2 (seizures)	2 (seizures)	25/NK	NK	NK	NK
Shen 2013 [56]	Dexmedetomidine 271.9±20.0, propofol 254.5±29.4	Dexmedetomidine 31.7±7.0, propofol 29.6±5.9	0	0	NK	1 (propofol group)	3 (dexmedetomidine 1, propofol 2)	2 (dexmedetomidine n = 1, propofol n = 1) intraoperative
Shinoura 2013 [57]	NK	NK	6 (n = 2 air embolism, n = 1 seizure, n = 1 motor neglect, n = 1 somnolence, n = 1 no wake up after GA)	6 (n = 2 air embolism, n = 1 seizure, n = 1 motor neglect, n = 1 somnolence, n = 1 no wake up after GA)	1/NK	NK	NK	NK
Sinha 2007 [58]	376.7±105.6 [240–480]	NK	1 (restlessness)	2 (restlessness and hypoxia)	4 (no BIS n = 3, BIS n = 1) / NK	2	8	8 (postoperative)
Sokhal 2015 [59]	268±45,7 [165–390]	NK	1 (brain bulge)	1 (brain bulge)	5/5 (propofol n = 2, dexmedetomidine n = 3)	4 (propofol n = 3, dexmedetomidine n = 1)	9 (propofol)	0 (intraoperative)
Souter 2007 [60]	NK	NK	0	0	1(SAS group)/6	0	1	NK
Wrede 2011 [61]	NK	NK	0	0	NK	NK	NK	NK
Zhang 2008 [62]	NK	NK	6 (n = 4 brain bulge, n = 2 somnolence)	0	2/NK	0	NK	NK

AC, awake craniotomy; LMA, laryngeal mask airway; min., minutes; n =, specified number of patients; NK, not known; PON(V), postoperative nausea (and vomiting); SD, standard deviation; SpO2, peripheral oxygen saturation. Data are presented as numbers of patients, or mean ± standard deviation or [range].

**Table 5:** Patient outcomes.

Study	New neurological dysfunction	Persistent neurological dysfunction >6months if not otherwise stated	Mortality	Postoperative intracranial haematoma	Tumour total resection	Length of hospital stay in days (mean and standard deviation, if not otherwise stated)
Abdou 2010 [17]	NK	NK	0	NK	NK	3.1 ± 1.1, range [1–5]
Ali 2009 [18]	1	0	0	NK	8	3.8 ± 4.15
Amorim 2008 [19]	2	1	0	0	8	NK
Andersen 2010 [20]	NK	NK	0	NK	NK	NK
Beez 2013 [21]	NK	NK	0	NK	NK	NK
Bilotta 2014 [10]	7 (only for language described)	3 (only for language described)	0	NK	13	NK
Boetto 2015 [22]	NK	0	0	0	NK	NK
Cai 2013 [23]	4	0	0	NK	NK	NK
Chacko 2013 [24]	8	NK after 6 months but after 40 months only 3 of the new remained	1	NK	NK for all patients	NK
Chaki 2014 [25]	NK	NK	0	NK	NK	NK
Conte 2013 [26]	NK	NK	NK	NK	NK	NK
Deras 2012 [27]	NK	NK	0	3	NK	NK
Garavaglia 2014 [28]	0	0	0	0	NK	NK
Gonen 2014 [29]	17	0	0	NK	89	4,96±4,69 of all 4 groups
Grossman 2007 [30]	NK	NK	1 (<12h)	NK	NK	NK
Grossman 2013 [31]	92 (n = 71 young+n = 21 elderly)	NK	5 (n = 2 young+n = 3 elderly)	12 (9 young + 3 elderly)	343 (n = 272 young + n = 71 elderly)	6.6 ± 7.5 elderly vs. 4.9 ± 6.3 young
Gupta 2007 [32]	NK	NK	NK	NK	10	5.69±5.3 range [2–28]
Hansen 2013 [33]	8	NK	0	NK	29	NK
Hervey-Jumper 2015 [34]	58	16 (3 months)	0	3	NK	Median 3, range [2–20]
Ilmberger 2008 [35]	41	14	0	NK	74	NK
Jadavji-Mithani 2015 [36]	NK	NK	0	NK	NK	NK
Kim 2009 [37]	74	34 (1 month)	0	NK	199	4 [3–53]
Li 2015 [38]	49	1	0	NK	53	NK
Lobo 2007 [39]	NK	NK	0	NK	NK	NK
Low 2007 [40]	6	1	0	1	11	5.5 [11–16] for n = 16 patients
McNicholas 2014 [41]	7	0	0	NK	NK	NK
Nossek 2013 [42]	30 (n = 6 failure group + n = 24 not failure group) (only speech deterioration assessed)	13 (n = 4 failure group + n = 9 not failure group) (only speech deterioration assessed)	1	20 (n = 4 failure group + n = 16 not failure group)	343	The length of hospital stay was significantly longer for the failure group than the successful group (8.0 ± 10.1 vs. 4.9 ± 6.2).
Nossek 2013 [43]	54 (n = 12 seizure + n = 42 nonseizure)	0	1 (non-seizure)	21 (n = 3 seizure + n = 18 non-seizure)	379	4±3 seizure, 3±3 non-seizure
Olsen 2008 [44]	NK	NK	0	NK	NK	NK
Ouyang 2013 [45]	NK	NK	0	2	NK	NK
Ouyang 2013 [46]	NK	NK	0	2	NK	NK
Pereira 2008 [47]	NK	23 (n = 3 worsening of pre-existing dysfunction at 6 month in group A <8/2004; n = 20 worsening of pre-existing dysfunction at 6 month in group B >8/2004)	1	2 (n = 1 group A (<8/2004); n = 1 group B (>8/2004))	9 (n = 4 group A (<8/2004); n = 5 group B (>8/2004))	NK
Peruzzi 2011 [48]	4	NK	0	NK	22	3.5 range [3–5]
Pinsker 2007 [49]	14	8 (3 months)	0	NK	40	NK
Rajan 2013 [50]	NK	NK	0	NK	NK	3±3.4
Rughani 2011 [51]	6	2 (1 month)	0	NK	20	3.7 range [2–8]
Sacko 2010 [52]	7	NK	0	4	80	median 4.5
Sanus 2015 [53]	4	0	0	0	20	median 5
See 2007 [54]	5	1 (after discharge)	0	0	NK	median 9 [3–77]
Serletis 2007 [55]	20	12	NK	NK	NK	NK
Shen 2013 [56]	NK	NK	0	NK	NK	NK
Shinoura 2013 [57]	8	3 (1 month)	0	NK	54	NK
Sinha 2007 [58]	10	0	0	NK	NK	13.3±4.2
Sokhal 2015 [59]	14	12	0	1	NK	7±5 [3–30]
Souter 2007 [60]	NK	NK	0	NK	NK	NK
Wrede 2011 [61]	2	NK	0	NK	NK	NK
Zhang 2008 [62]	13 (n = 6 group 1; n = 1 group 2; n = 6)	7 (n = 2 group 1; n = 1 group 2; n = 4 group 3)	0	1	14	NK

n =, specified number of patients; NK, not known; vs., versus.

#### Risk of bias within and across studies

The risk of bias was assessed with the Cochrane Collaboration’s risk of bias tool (S2 Table) for the RCTs and for the remaining studies with the Agency of Healthcare Research and Quality (AHRQ-tool) [12] (S3 Table). Both RCTs [36,56] and the pseudo-RCT [36] showed a high risk of selection and performance bias. Observational studies showed a high risk of detection bias and confounding bias. Furthermore, they showed a varied degree of other risks of biases inherent to the study design.

#### Results of individual studies

We divided the identified records into three subtopics according to the used anaesthetic technique: Nineteen studies reported the asleep-awake-asleep (SAS) respectively sleep-awake (SA) technique [20–23,25–27,37–39,44–46,50,51,53,56,57,60], twenty-eight reported monitored anaesthesia care (MAC) [10,17–19,24,28–32,34–36,40–43,47–49,52,54,55,58–62] and one used the awake-awake-awake (AAA) technique [33]. Of note, Souter et al. have used the SAS as well as the MAC technique in their patients [60].

### Synthesis of results

General considerations for AC are provided in the S2 File.

#### SAS—asleep-awake-asleep technique

The protocols of the nineteen identified articles [20–23,25–27,37–39,44–46,50,51,53,56,57,60], reporting the SA(S) technique, showed a huge variability in the anaesthesia conduction, but all kinds of this technique were feasible and safe for the patients. A total intravenous anaesthesia (TIVA) with propofol and remifentanil or fentanyl for the first asleep phase was used in fourteen trials [20–23,26,27,37,39,44–46,51,56,60]. Two studies used only propofol as sedative [25,38]. Dexmedetomidine, an alpha-2 adrenoceptor agonist, enables sedation, anxiolytic effects, and analgesia. It was successfully used for AC since 2001 [64]. Dexmedetomidine was applied in four studies, either combined with remifentanil [34,56], or propofol [50], or with remifentanil and propofol together [53,57]. The use of dexmedetomidine seems to show several advantages in AC. Shen et al. compared the effect of dexmedetomidine- to propofol-based SAS technique [56]. They showed that patients in the dexmedetomidine group had a shorter arousal time after the first asleep phase and a higher degree of surgeon satisfaction. Fast and sufficient recovery after craniotomy is a crucial factor for successful awake cortical mapping within adequate surgery time. A further study, showed reduction of pain induced haemodynamic reactions to pinning and incision, when AC with propofol, dexmedetomidine and local anaesthesia was performed (n = 101), compared to balanced GA (n = 77) [50]. This could partly be explained by the analgesic and sympathic blockage effect of dexmedetomidine. Furthermore, the patients needed less intraoperative vasopressors and opioids compared to the GA group. Also postoperative requirement of opioids and antiemetic drugs was reduced in the AC group. Of note, in contrast to the AC group, a RSNB was not performed in all GA patients, which maybe accompanied by more opioid application and consecutive nausea. Conversely they observed more oxygen desaturations (S_a_O_2_ <90%) in the AC group, despite the absence of respiratory suppression by dexmedetomidine. This might be explained by the propofol saving effect of dexmedetomidine, which bears the risk of over sedation with propofol, especially during the painful beginning of the surgery. Of note, only one AC patient required the placement of a LMA. In contrast two GA patients showed significant postoperative desaturations and one of them required a re-intubation.

The airway in the included studies was secured either with a laryngeal mask (LMA) [21,25,26,38,45,46], an endotracheal tube in all [56],respectively one patient [20,44] or an oesophageal naso-pharyngeal catheter [23]. One study, which used a TIVA, did not mention the utilized airway device, they only reported naso-pharyngeal airway [53] and another one reported only an "oral airway" for five patients [51]. Twelve studies [21,23,26,56] used controlled ventilation, the others maintained spontaneous breathing. A nasal cannula with spontaneous breathing was used in one trial [34,50] and Shinoura et al. did not report the ventilation mode [57].

**Fig 2:**
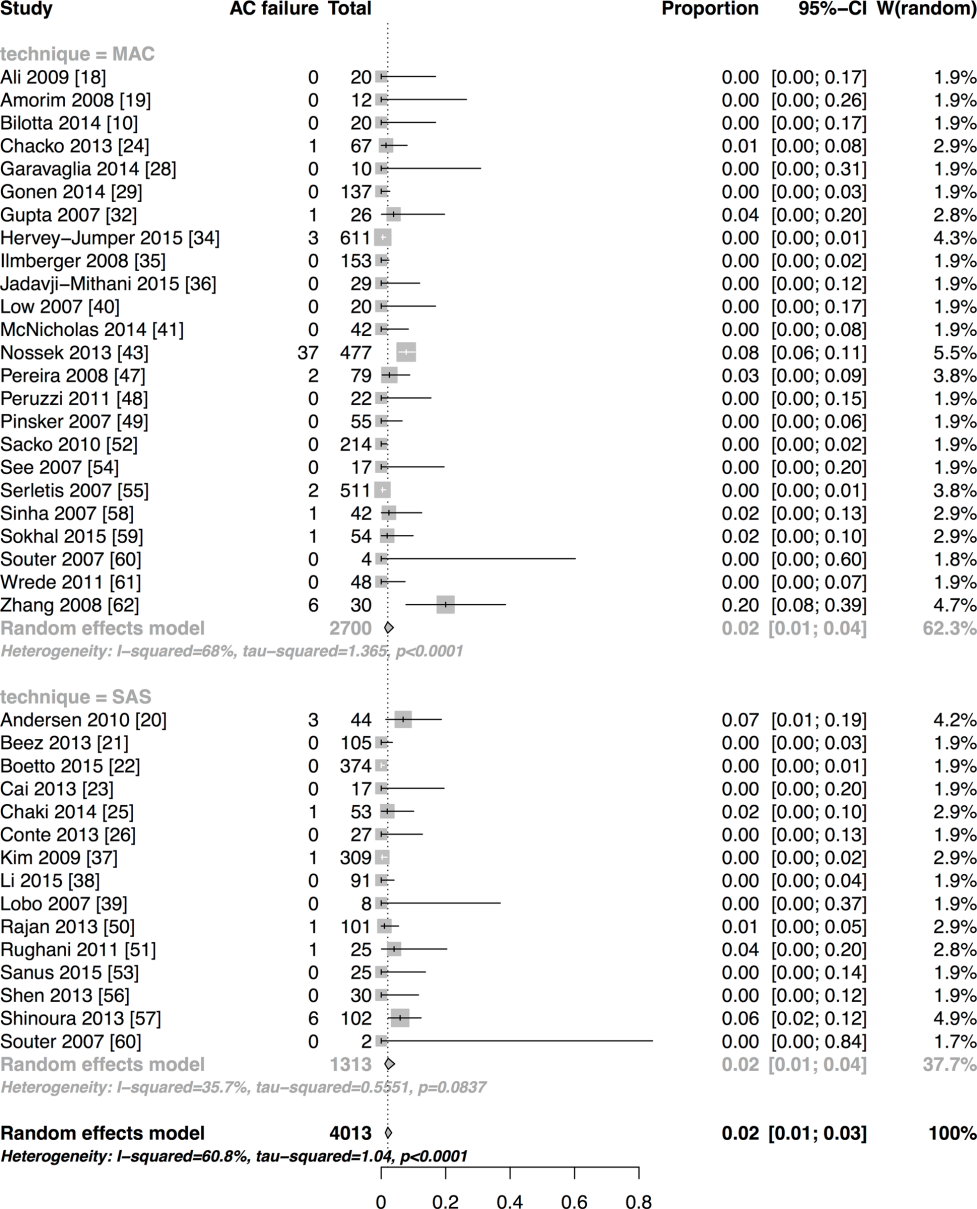
Forrest plot of awake craniotomy failure. The summary value is an overall estimate from a random-effect model. The vertical dotted line shows an overall estimate of outcome proportion (based on the meta-analysis) disregarding grouping by technique. Of note, Souter et al. [60] have used both anaesthesia techniques.

Once the dura was opened and brain exposed, propofol was terminated and remifentanil and dexmedetomidine infusions were reduced or also stopped to allow patient awakening and removal of the airway device. In the study, which used the naso-pharyngeal catheter, the proximal balloon sealing the naso- and oro-pharyngeal cavities was deflated to allow patient vocalisation [23]. Dexmedetomidine was also successfully used after cessation of propofol and fentanyl, during the awake resection phase of the SAS technique [60]. Of note, this study reported the SAS technique for only two patients and concurrently the MAC technique for four patients. The second asleep phase was not described in detail in all included studies, but it consisted of sedative anaesthesia, remaining spontaneous breathing during wound closure up to controlled ventilation with endotracheal intubation like in the study of Deras et al. [27]. Only four studies used a sleep-awake (SA) protocol for some patients [21] respective all patients [20,26,44]. Almost all patients undergoing SA(S) management underwent successful AC and the failure rate was minimal with 13 out of 1313 procedures (where failure rate was reported, and excluding the duplicate studies [27,44]). The meta-analysis showed a proportion of 2% [95%CI: 1–4] (Fig 2).

#### MAC—monitored anaesthesia care

Defined by the "American Society of Anesthesiologists" (ASA) this technique enables purposeful patient response to tactile or verbal stimulation, while preserving spontaneous ventilation without any airway instrumentation [8]. Twenty-eight included studies reported the successful use of MAC in AC [10,17–19,24,28–32,34–36,40–43,47–49,52,54,55,58–62]. Except of three studies, which only used local infiltration anaesthesia [36,48,61] respectively one, which did not mention the use of infiltration anaesthesia [17], all others applied an additional RSNB at the beginning of the surgery. The airway was secured with an oxygen mask, a nasal cannula or an additional nasal trumpet under maintained spontaneous breathing. Except in two studies [17,61], the used anaesthetics consisted of all possible combinations of fentanyl, remifentanil, propofol, midazolam and dexmedetomidine. Abdou et al. applied in the same syringe a mixture of ketamine and propofol 1:1 ‘‘ketofol” [17], and Wrede et al. used piritramide and midazolam for their conscious sedation [61]. Propofol and remifentanil were mostly discontinued 15 minutes before brain mapping and the patients did not receive any sedation and analgesia during the "awake" phase in thirteen studies [10,17,24,28–31,36,41–43,49,54], respectively three studies which applied only opioids if needed [34,47,52]. Three studies continued conscious sedation with propofol in a reduced dosage also during the awake phase [18,32], and the awake anaesthesia management is unknown for six studies [19,35,55,58,61,62]. Dexmedetomidine (around 0.1–0.7 μg kg^-1^ h^-1^) was continued in totally 36 procedures during the "awake" phase [48,59,60]. It could be shown, that dexmedetomidine has a minimal interference with electrocorticography (ECoG) during AC in a dosage of 0.2–0.5 μg kg^-1^ h-1 [60]. Anaesthesia for the end of surgery was not described in detail in the identified MAC studies, but it may be assumed that the initial regime was resumed until skin closure. Interestingly, Peruzzi et al. used additional sevoflurane until the opening of dura mater, to decrease the amount of propofol [48].

Grossman et al. included in their study 90 elderly patients, with a mean age of 71.7±5.1 years, of totally 424 patients [31]. Preservation of the neurological status has a strong impact on the quality of life in especially this population. They showed that a maximum gross total resection (GTR) of high-grade glioma under AC is feasible, without increased mortality or postoperative morbidity (including postoperative neurological complications) in elderly patients, compared to the younger one [31]. Furthermore, their survival increases significantly compared to restrictive treatment like subtotal resections and biopsies. MAC for AC was also used efficaciously in five elderly patients (>60 years), with complex co-morbidities [28]. Intraoperative hypoxia was reported for five patients [36,59], but all cases could be resolved with simple dose reduction and oxygen application. One large retrospective study (n = 611) used all possible combinations of propofol, remifentanil and dexmedetomidine in patients with significantly different baseline characteristics [34]. Only high-risk patients (high body-mass-index (BMI), high tumour mass, high blood loss estimated) (n = 8) received a LMA for the initial procedure [34]. The total rate of AC failure in all studies using the MAC technique and reporting the failure rate was 81 of totally 3616 procedures. Excluding the duplicate study of Nossek et al. [42] and Grossman et al. [31] which contained partially the same patients like the larger second study [43], our meta-analysis calculated with the random effects model revealed a proportion of a 2% failure rate [95%CI: 1–4] in 2700 procedures, which reported AC failure (Fig 2).

#### AAA—Awake-awake-awake technique

Hansen et al. were the first, who reported the awake-awake-awake technique avoiding sedatives in 47 patients undergoing 50 AC procedures by using RSNBs, permanent presence of a contact person, and therapeutic communication [33]. Instead of using premedication with benzodiazepines, a strong pre-operative confidence with calming the patient was established during an extensive pre-operative personal visit of the attending anaesthesiologist. Subsequently the anaesthesiologist continuously guided the patients intraoperatively with strong rapport, physical contact and therapeutically communication. This included hypnotic positive suggestions like reframing disturbing surgery related noises and dissociation into a "safe place". Only two-thirds of the patients requested remifentanil with an average total dose of 156μg. Intraoperative vigilance tests showed equal or higher scores than preoperative tests. In the postoperative interview conducted in twenty-two patients, 73% of patients reported a lack of any discomfort, 95% felt “adequate prepared”, and 82% did not experience any fear at all. BIS monitoring was applied in all patients. The AC failure rate was minimal with one patient out of 50 AC procedures. This patient experienced general seizure, which could not be handled only with cold saline solution or minimal doses of propofol, but the surgery was smoothly continued in GA. A meta-analysis could not be performed for the AAA technique due to only one study reporting it.

#### Adverse events

A reasonable meta-analysis and logistic meta-regression could only be performed for four outcome variables: AC failures, seizures, conversion into general anaesthesia and new postoperative neurologic dysfunction based on the anaesthetic approach of MAC or SAS. The other variables were not reported frequently enough in the included studies for both kinds of anaesthesia technique. Mortality was reported in thirty-eight studies, but not included in the meta-analysis as a single outcome variable due to the extremely rare event rate. It was integrated in the composite outcome analysis together with AC failure and intraoperative seizure.

#### AC failure

Our primary outcome of interest was the failure rate of AC, depending on the used anaesthesia technique. The meta-analysis for the proportion of awake craniotomy failures, depending on the used anaesthetic approach (MAC vs. SAS) included thirty-eight studies (Fig 2) [10,18–26,28,29,32,34–41,43,47–62]. It included the largest of the duplicate studies and excluded the smaller ones [27,42,44], which have also reported this outcome, according to Tramer et al. [14] and van Elm et al. [15]. The particular reasons for AC failures are shown in Table 4 and included all cases where a complete intraoperative awake monitoring of the brain function during the tumour resection could not be achieved. Of note, an AC failure was not only restricted to the cases, where conversion to GA was required. The proportion of AC failures was 2% [95%CI 1–3], and the studies showed a substantial heterogeneity (I^2^ = 61%) (Fig 2). The relationship of the used technique (SAS/ MAC) as a possible source of the heterogeneity was explored using logistic meta-regression. The OR comparing SAS to MAC was 0.98 [CI95%: 0.36–2.69]. The employed anaesthesia technique did not explain a substantial portion of the heterogeneity between studies (QM = 0.001, df = 1, p = 0.972), and the test for residual heterogeneity was significant (QE = 93.70, df = 37, p < 0.001).

#### Conversion into general anaesthesia

**Fig 3:**
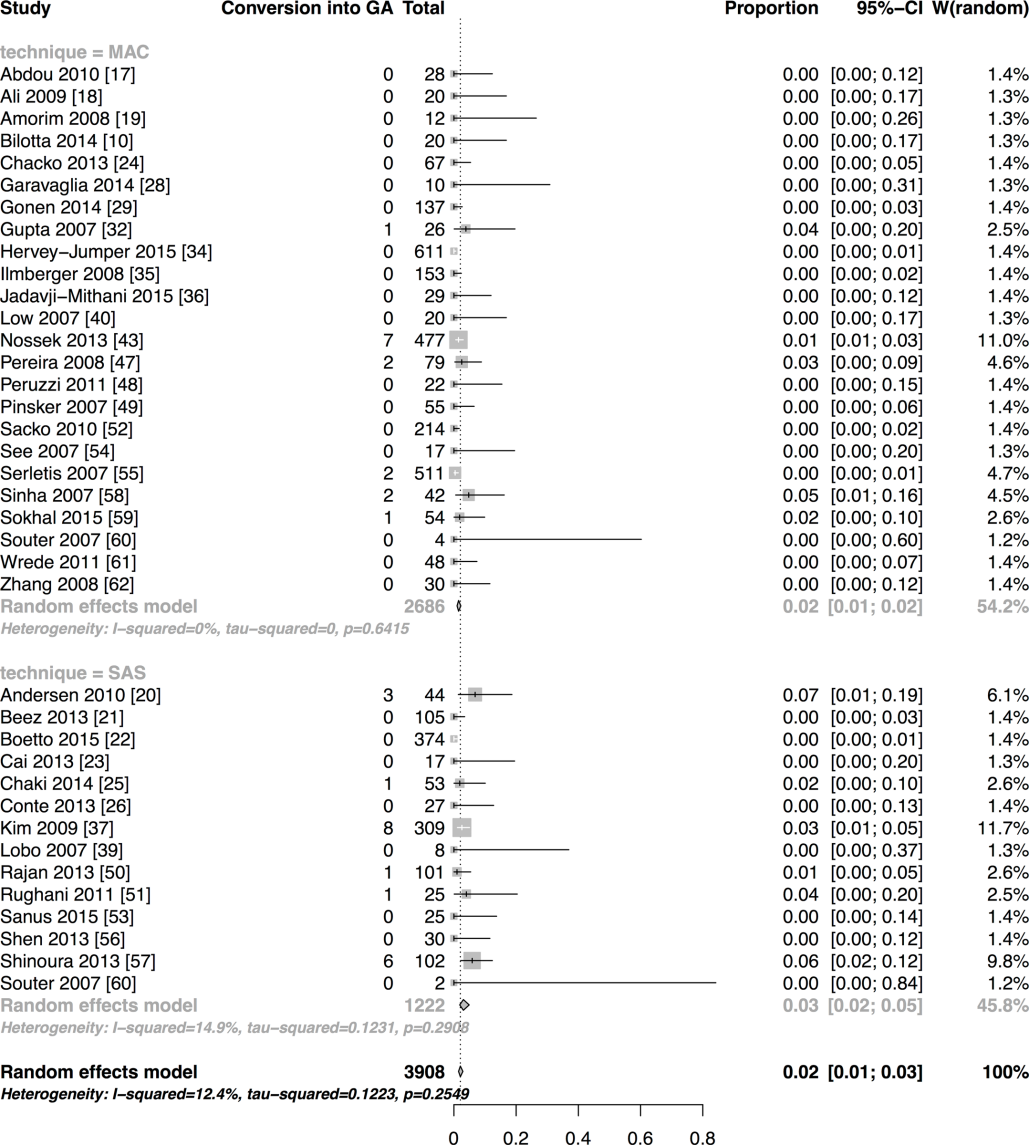
Forrest plot of conversion into general anaesthesia. The summary value is an overall estimate from a random-effect model. The vertical dotted line shows an overall estimate of outcome proportion (based on the meta-analysis) disregarding grouping by technique. Of note, Souter et al. [60] have used both anaesthesia techniques. GA, general anaesthesia.

The discrepancy between the numbers of required conversion to GA and AC failure rates may be explained as follows: Not every AC failure required conversion into GA and not every conversion into GA was performed during the awake tumour resection phase, but also at the end of surgery, where it did not compromise the success of AC, like in the study of Sinha et al. [58]. Forty-two studies reported 47 unplanned conversions into GA during totally 4971 AC procedures [10,17–29,31–37,39,40,42–44,47–62]. The particular reasons for unplanned conversion into GA are shown in Table 4. After exclusion of the duplicate studies [27,31,42,44] and the AAA study of Hansen et al. [33], our meta-analysis showed a total proportion of conversion into GA of 2% [95%CI 1–3] (Fig 3). Logistic meta-regression was also performed for this outcome, to analyse if the used technique (SAS/ MAC) may explain the differences between the studies. The OR comparing SAS to MAC was 2.17 [95%CI: 1.22–3.85] and the likelihood ratio test (LR test) showed a significant p-value of 0.03. However, the predicted proportion of conversions in the MAC and SAS group were not substantially different (MAC: 2% [95%CI: 1–2], SAS: 3% [95%CI: 2–5]).

#### Seizures

Threatening adverse events during AC are seizures. The most seizures in the included studies were triggered by electrical cortical stimulation and were self-limited after cessation of cortical stimulation. The other could be treated with cold saline solution, or finally with anticonvulsive medication, or low doses of propofol, thiopental or benzodiazepines. Discontinuation of AC was rarely necessary. Thirty-nine studies reported the incidence of intraoperative seizures and their consequences [10,17–29,31–39,42–44,47,49–55,57–60,62]. The total number of performed AC procedures in these studies was 4942 and 351 (7.1%) intraoperative seizures were reported (Table 4). Only twenty-three (0.5%) intraoperative seizures led to a failure of AC, but they were resolved without any serious problems and the surgery was continued in GA [33,34,42,43,55,57]. Interestingly, the AAA technique showed a high proportion of eight seizures in fifty AC procedures, but only one led to AC failure due to required intubation [33].

**Fig 4:**
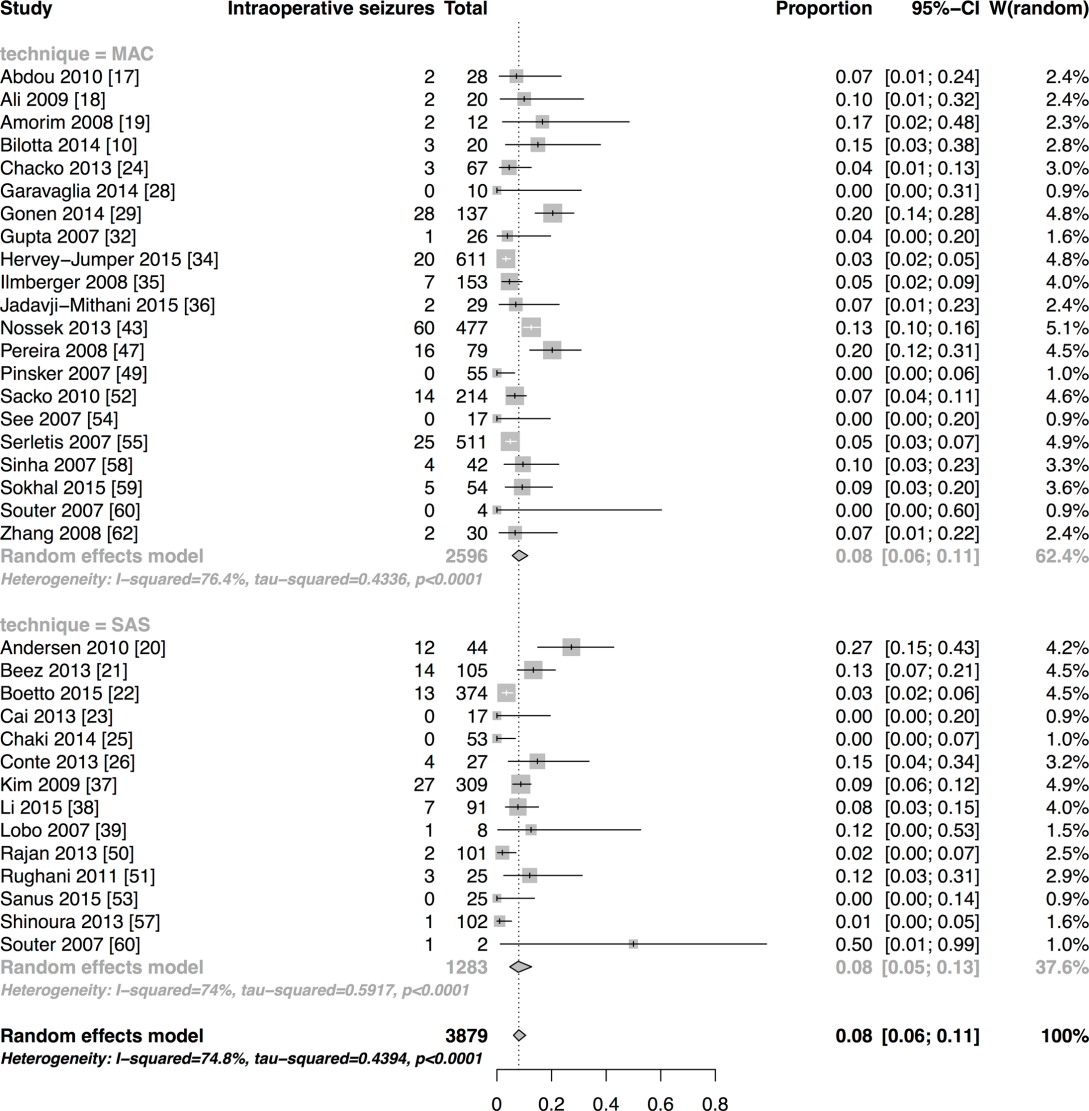
Forrest plot of intraoperative seizures. The summary value is an overall estimate from a random-effect model. The vertical dotted line shows an overall estimate of outcome proportion (based on the meta-analysis) disregarding grouping by technique. Of note, Souter et al. [60] have used both anaesthesia techniques.

Intraoperative seizures were more common in younger patients and those with a history of seizures [31,42]. A meta-analysis was performed for thirty-four studies, [10,17–26,28,29,32,34–39,43,47,49–55,57–60,62], which used the MAC and SAS technique, excluding the duplicate studies from Tel Aviv [31,42] and Glostrup [27,44]. Meta-analysis showed an estimated proportion of seizures of 8% [95%CI: 6–11] with substantial heterogeneity between studies (I^2^ = 75%) (Fig 4). In the meta-regression analysis, the techniques used did not explain the differences in the studies (QM < 0.001, df = 1, p = 0.983). The OR comparing SAS to MAC technique was 1.01 [CI95%: 0.52–1.88].

#### Postoperative neurological dysfunction (new/ late)

**Fig 5:**
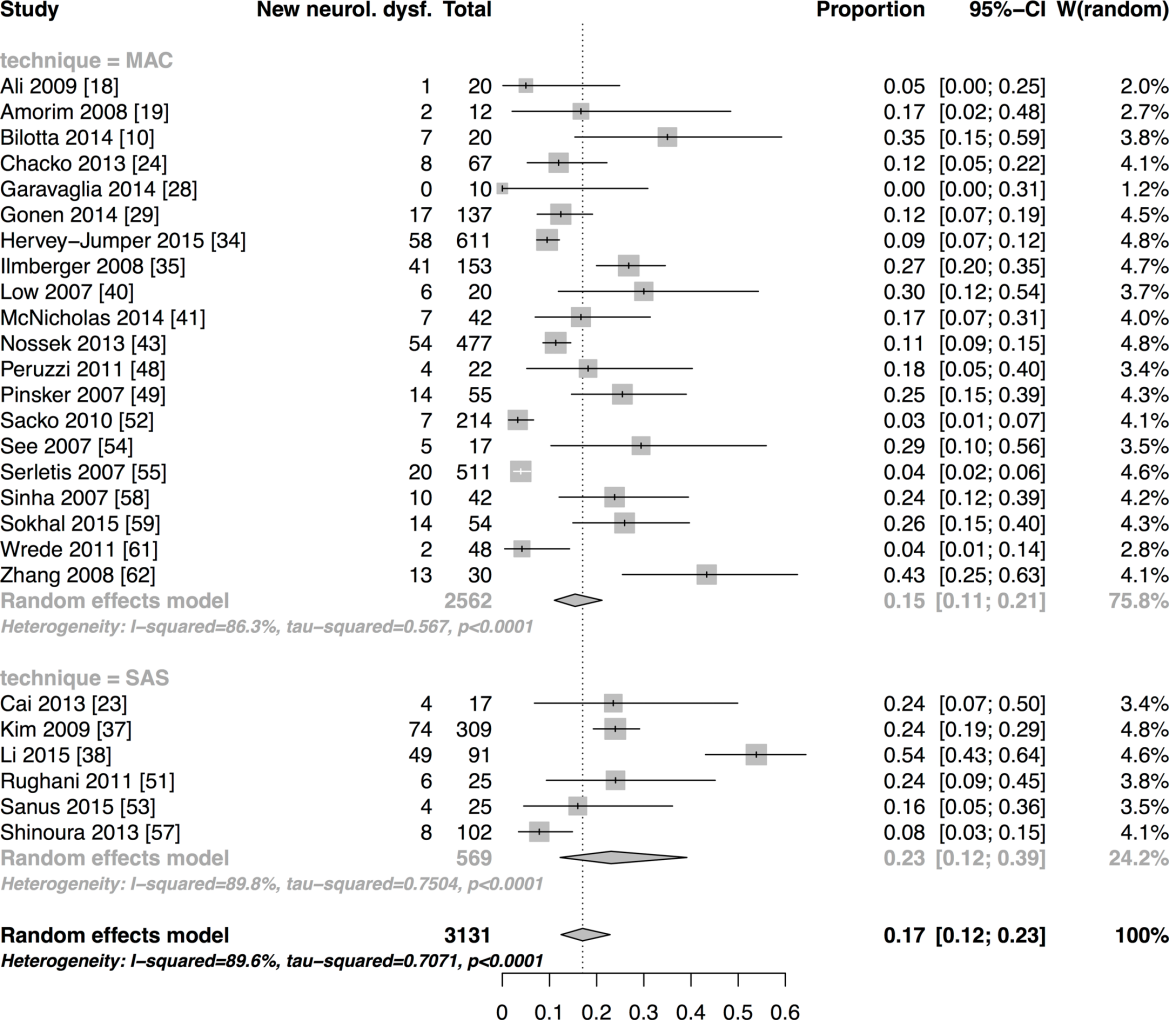
Forrest plot of new neurological dysfunction. The summary value is an overall estimate from a random-effect model. The vertical dotted line shows an overall estimate of outcome proportion (based on the meta-analysis) disregarding grouping by technique. Neurol. dysf., neurological dysfunction.

Description of particular postoperative neurological dysfunctions differed significantly in the included studies. Therefore we have subsumed all kinds of new neurological dysfunctions under these superordinate two outcome variables. Of note, we did not include data of patients with deterioration of a pre-existing neurological dysfunction. Twenty-nine studies [10,18,19,23,24,28,29,31,33–35,37,38,40–43,48,49,51–55,57–59,61,62] reported new postoperative neurological dysfunctions after 565 (14.0%) of totally 4029 AC procedures. A later follow up result (six months) was provided for 279 of these patients with new neurological dysfunction. It showed a persistent neurological dysfunction in 64 patients. Of note, late neurological outcome after six months was reported in only seventeen studies comprising 2085 AC procedures in total. Considering twenty-six studies [10,18,19,23,24,28,29,34,35,37,38,40,41,43,48,49,51–55,57–59,61,62], which were reasonable included in our meta-analysis, the proportion of new neurological dysfunction was estimated to be 17% [95%CI: 12–23], with a high heterogeneity (I^2^ = 90%) (Fig 5). Meta-regression analysis did not reveal a difference depending on the anaesthesia technique (MAC/ SAS) (QM = 1.52, df = 1, p = 0.217), with an OR of 1.66 [95%CI: 1.35–3.70]. Furthermore, there is a large proportion of residual heterogeneity (QE = 187.55, df = 24, p < .0001), which cannot be explained by the applied anaesthesia technique. However, it has to be noted that there are only six studies available in the SAS group.

#### Other adverse events/outcomes

The other extracted adverse events and outcome data are shown in Tables 4 and 5. Mortality was very low with 10 patients (0.2%) of all forty-four studies comprising 5381 patients, which reported the outcome variable mortality (Table 5). Of note, two deaths include probably duplicate patients [42,43] to the study of Grossman et al. [31]. Furthermore, we have only included deaths within 30 days after surgery in this analysis.

Interestingly 44% of the patients in the AAA approach of Hansen et al. experienced arterial hypertension [33], but this refers only to the test phase. During the pinning, craniotomy and tumour resection there were only 5 patients with 10–20% increase in blood pressure.

#### Additional analyses

The analysis of the composite outcome, including AC failure, intraoperative seizure and mortality was based on forty-one studies (S1 Fig) [10,17–26,28–30,32,34–41,43,46–62]. Of note, intraoperative seizure events, which concurrently led to an AC failure, were counted only once for this composite outcome. The total proportion was estimated to be 8% [95% CI: 6–11], with 8% [95% CI: 6–12] in the MAC group and 8% [95% CI: 5–12] in the SAS group. Logistic meta-regression did not show a difference of the event rate depending on the technique (MAC/ SAS). The OR was 0.9 [95% CI: 0.47–1.76] and the residual heterogeneity I^2^ = 80%.

Sensitivity analysis, by including only prospectively conducted trials, was performed to look at the robustness of our findings in the main summary measure analyses of the four outcomes (AC failure, conversion to GA, intraoperative seizure and new neurological dysfunction) and the additional analysis of the composite outcome. Sensitivity analysis referred to eighteen trials [10,17,18,21,22,25,26,28,30,32,35,36,38,47,52,55,56,61], after exclusion of one duplicate study [27]. Of note, it was not possible to predict an estimate for the outcome new neurological dysfunction in the SAS group, because only one prospective SAS study provided data for this outcome [38].

The proportions of outcomes were slightly lower in prospective studies compared to results from the main analysis, which is shown in S2 Fig. The logistic meta-regression models using the independent variables anaesthesia technique (MAC/ SAS) and prospective studies (yes/ no) showed only very small and statistically not significant differences.

## Discussion

Our systematic review has pointed out forty-seven studies addressing three main topics: SAS-, MAC- and AAA-technique of anaesthesia management for AC since 2007. We identified only two small RCTs [32,56] and one pseudo-RCT [36]. These were as well as the remaining observational studies of moderate to low methodological quality. In summary all three anaesthetic approaches were feasible and safe. But our results have to be seen within their limits. Nine of the identified forty-seven studies reported partially duplicate patient data, first the studies of Ouyang et al. [45,46], second the studies from Tel Aviv [31,42,43], third the studies from Glostrup [20,44] and at least the studies of Boetto and Deras et al. [22,27]. Furthermore, the results from our meta-analysis are dominated by two larger retrospective studies with 611 [34], respectively 477 patients [43] and one prospective study with 511 patients [55]. This was partially taken into account in our meta-analysis with the use of the random effects model, which applies less weight to large studies than fixed effect models. The meta-analyses revealed no statistically significant differences of AC failures, intraoperative seizures, new neurological dysfunctions, and the composite outcome (AC failure, intraoperative seizure, mortality) depending on the use of SAS or MAC technique. We found a substantial heterogeneity between the included studies and only the heterogeneity for conversion to GA showed a possible significant connection to the anaesthesia technique in the logistic meta-regression analysis. This analysis suggested significantly more unplanned conversions into GA with the use of SAS than MAC anaesthesia technique. However, this result was mainly depending on one high risk of bias retrospective SAS study with 6 events in 102 patients [57]. Removing this study abolishes the significant difference between the techniques. Of note, two of the patients in this study required conversion into GA due to an air embolism, which was most likely related to the half-sitting patient position and not the used anaesthesia technique [57]. Although air embolism was not analysed in detail in our SR, this was the only study, which reported a failure of AC due to this life-threatening adverse event. Furthermore, the sensitivity analysis, which included only prospective studies, confirmed the weakness of the result obtained by the main meta-regression analysis. A significant difference between the used anaesthesia techniques in regard to conversion to GA could not be revealed by the sensitivity analysis anymore. The decision to perform a sensitivity analysis by including only prospective studies and not the largest ones, was justified by the increased risk for confounding in larger studies due to a prolonged study duration. The most studies with more than 100 AC procedures, where performed during 5–8 [22,31,42,43,45,46,52] or 10–18 years [34,35,37,55,57]. The probability of a continuously same anaesthesia or AC surgery conduction in these observational studies during the large time-spans is very low. Our sensitivity analysis did also not reveal any statistical significant difference for the other four outcomes, which were included in the meta-analyses. Of note, the new neurological dysfunction outcome was only presented by one prospective study [38] in the SAS group. Therefore, we could not estimate the proportions for this outcome in the meta-analysis (S1 Fig). However, the main analysis included also only six studies in the SAS group [23,37,38,51,53,57] and the result was dominated by this prospective observational study of Li et al. in a Chinese population [28]. Although 53.8% of the 91 patients exhibited new neurological dysfunctions, these dysfunctions remained permanent only in 1 patient, which suggests that the aim of safe resection was achieved in the longer-term. Furthermore, the generalizability of their results is unclear, due to possible differences in the distribution of the Chinese language areas compared to Non-Chinese people. Therefore we suggest interpreting our result of the meta-regression analysis in regard to new neurological dysfunctions with caution. According to previous investigations [5], Nossek et al. showed a better neurological outcome in the AC, than in the failure group [42]. Furthermore, similar to other studies [65,66], Grossman et al. [31] could confirm a longer survival time depending on the extent of tumour resection. Of, note a selection bias in this analysis cannot be excluded, as there were most likely baseline differences between the patients who underwent AC with gross total resection and patients who underwent only biopsy or subtotal resection. Kim et al. underlined the importance of gross total resection particularly with regard to a significantly better neurological outcome [37].

Awake craniotomy is a demanding but safe procedure, none of the patients involved in the studies selected for this SR showed a serious adverse event, which could not be handled during AC. Well-considered patient selection has a big impact on the success of AC. More studies including multi-morbid or high-risk patients are required to confirm their eligibility to undergo AC as reported in four of our identified studies [28,34,43,45]. Pre-/ and postoperative MRI and neuropsychological testing were reported in almost all studies and should be performed routinely before AC. Additional recording of neurological exam videos before and after surgery may facilitate the neurological outcome measurement [47]. Bilotta et al. described their experience with perioperative language testing by an anaesthesiologist in twenty patients undergoing MAC technique for AC [10]. They pointed out the importance of perioperative language testing, also in settings without the presence of a professional language therapist. During the pre-operative language testing they identified patients with risk for postoperative language disturbances and patients with pre-operative deficits, which facilitated intraoperative identification of language deterioration. Furthermore, the patients were prepared for the upcoming intraoperative language testing tasks.

Administration of RSNBs, independent of the anaesthetic technique, has evolved as a safe and reasonable supportive procedure at the beginning of AC. This procedure appears to be superior over merely local scalp infiltration, as it blocks superficial as well as nociceptive afferents to profound tissues [67]. A recent systematic review and meta-analysis of RCTs evaluated postoperative pain after RSNB for craniotomy [67]. The published RCTs of RSNBs were small and of limited methodological quality, but the meta-analysis showed a consistent finding of reduced postoperative pain. Although RSNBs have potential complications, like local anaesthetic toxicity, hypertension, infection, haematoma, nerve injuries and inadvertent subarachnoid injection [68], this SR did not identify any adverse events associated with this procedure [67]. In contrast, a case series of McNicholas et al., including 42 patients with RSNBs, reported seven patients with transient postoperative facial nerve palsy. They recommend limiting the local anaesthetic volume for auriculotemporal nerve block to 3 ml, and staying above the level of the tragus. [41] The specific learning rate to apply adequate RSNB is about ten procedures [69].

Postoperative questionnaires in the study of Beez et al. [21] revealed only in 5.1% severe discomfort, while the preoperative preparation was rated adequate in 94.9%. Other studies support these findings with postoperative satisfaction rates of 96.5% up to 100% [20,44,47,52,60]. Degree of satisfaction measured by visual analogue scale (VAS) in one study [56], which compared propofol-based to dexmedetomidine-based SAS protocol, showed a high degree of satisfaction (VAS 92) in both patient groups. In contrast, the blinded surgeons`satisfaction was significantly higher in the dexmedetomidine group. Careful patient-positioning is a further crucial factor influencing the success of AC, due to patient comfort and compliance [21]. Active participation of the patients in the positioning phase supported probably the high patient satisfaction (84%) in a further study [27].

Avoidance of PONV is another contributing factor for patient satisfaction after AC. Beside this, PONV bears the risk of dehydration and in case of vomiting it could increase critically the intracranial pressure [70]. Incidence of Nausea within 24h after craniotomy in GA technique was reported with 30–70% [70], favouring the use of antiemetic prophylaxis. Fabling et al. showed a significant reduction of PONV by prophylaxis with low dose droperidol or ondansetron in their RCT [70]. Nausea was analysed intraoperatively in eleven of our included studies [17,18,20,27,30,36,44,51,54,56,59], and postoperatively in ten studies [17,18,30,33,45,46,50,51,54,58]. The intra- and postoperative incidences showed a range between 0 [18,30,46,51,59] and 30% [45,46]. The effect of antiemetic prophylaxis could not be evaluated for all of these studies, as it was not reported entirely. Ouyang et al. used ondansetron as well as dexamethasone and had a similar incidence of 30% as previously reported for patients receiving ondansetron [70]. Interestingly, preoperative midline shift of averagely 5.96mm did not enhance the risk for PONV [45], although it is an independent risk factor for intraoperative brain oedema. The tumour histopathology was also not associated with an increased incidence of PONV [46].

Usefulness of BIS, or equal monitoring of anaesthesia depth, remains debatable in patients with neurological disorders, or antiepileptic drug therapy. While one report a strong delay in actual BIS values and awareness in AC patients with lower values than 80 [71], others recommend its use for AC [72]. However, in our review there was no difference between the occurrence of AC failures in studies, which did not use any objective anaesthesia depth control [10,18–22,24,25,27–29,32,34–38,40–44,47,49–52,54,55,60,61] compared to studies, which used either RE or BIS monitoring [23,26,33,39,48,53,56,58,59,62]. Favourable evidence for using BIS in SAS was shown in one study, where the patients recovered faster if the BIS values were targeted to higher levels before commence of the awake phase [26]. Another study with MAC anaesthesia showed significantly reduced propofol and fentanyl dosages in patients with BIS monitoring compared to patients without [58]. This could have an impact on the success of awake surgery tasks. The influence of prior sedation on the cognitive and motoric ability to perform intraoperative tasks [73]. Reduction of propofol dosage was also the aim in a further of our included studies [48]. Interestingly, they used the volatile anaesthetic sevoflurane until the dura opening for this purpose. Due to the cerebral vasodilatative effect of sevoflurane, it is at higher risk for increasing intracranial pressure and brain swelling compared to intravenous agents, especially in patients with pre-existing intracranial hypertension [74]. Abdou et al. used so-called "ketofol" anaesthesia, comprising ketamine and propofol mixture in one syringe, to avoid the side effects of opioids and reduce the propofol requirement [17]. The generalizability of this method remains questionable, as this mixture is not approved in many countries, like e.g. Germany. Clear evidence exists for meticulous preparation to handle intraoperative seizures. Most seizures occur in regard to cortical stimulation, which should be discontinued immediately and direct cortex irrigation with cold saline solution should take place [75]. This method was used throughout all AC studies, which we have analysed in this review. Only resistant seizures were treated escalating with small doses of benzodiazepines, propofol or thiopental, antiepileptic drugs, or GA. Furthermore, it is beneficial to recognise already preoperatively patients at higher risk for intraoperative seizures. Patients with tumours in the frontal lobe [43], and especially the supplementary motor area [29] showed a higher incidence of intraoperative seizures and this should be considered during the patient preparation. Furthermore, younger patients, patients with low-grade glioma and history of seizures were prone for intraoperative seizures [29,31,37,43]. Adequate treatment with antiepileptic drugs (AEDs) could not prevent the occurrence of intraoperative seizures [29,42], similar to previous findings [76]. Moreover caution is required for patients receiving phenytoin perioperatively, as it probably increases the risk for communication failures during AC [42]. Length of hospital stay was rarely described in the included studies (Table 5) and is very difficult to compare between different healthcare systems, thus a reasonable meta-analysis was not feasible. Interestingly, one study showed a substantial longer length of stay (13.3±4.2 days) than the others [58], which is probably explained by their hospital policy. In contrast, there is some evidence that AC can also be performed as a same day surgery procedure [77]. AC failure rate was our primary outcome of interest, as it plays a crucial role in the extent of the tumour resection, the consecutive postoperative survival time and neurological outcome of the patients [5]. Shinoura et al. could confirm a significantly impaired neurological function after failed AC [57]. Sacko et al. additionally pointed out the importance of successful awake surgery for tumours near eloquent brain areas [52]. They found a significantly better neurological outcome, higher proportion of GTR and shorter hospital length of stay in patients undergoing AC compared to GA. Ali et al. had similar results, favouring AC [18]. In contrast, Gupta et al. could not find any significant differences between GA and AC in their RCT, except for the procedure time, which was significantly shorter in the GA group [32]. The total reported failure rate for all three AC techniques (excluding the partially duplicate studies [27,42,44] was 1.7% (68 out of 4063 patients). This was confirmed by our meta-analysis of the MAC and SAS studies (Fig 2). Due to the heterogeneity and low quality of all included studies, this result has also to be seen within its limits. Only a large-scale multi-centre randomised controlled trial, with a standardised perioperative protocol would enable a definitive distinction of these two procedures. Furthermore, the AAA technique [33], with its low failure rate of 1 out of 50 patients seems to have potential for implementation in AC, but further clinical data are required to confirm the feasibility of this technique to larger populations.

Dexmedetomidine has been successfully used for MAC as well as the SAS technique in our included studies. Further investigations are required to show a potential significant superiority of dexmedetomidine to especially propofol.

### Limitations

First, we accessed only two databases and restricted our search to English language, which might not have identified all clinical studies meeting our inclusion criteria. Second, we focused our search on the years 2007–2015, to analyse the recent development of anaesthesia techniques for AC. One justification for our chosen time-span is the continuous development of the anaesthetics and our aim to provide a SR for the actually usually used anaesthetics. Another one is that the information quality of clinical articles is significantly depending on the reporting quality. As the most of the identified studies were of observational nature, we have decided to include only studies since 2007, when the latest STROBE statement for improving reporting quality of observational trials was published [78]. Of note, some of our included studies were already published in 2007 before the latest STROBE statement release. Due to our specific search strategy we identified only forty-seven studies, which were mostly observational, retrospective and heterogeneous. All studies, including the two small RCTs with 26 [32] and 30 patients [56], and one pseudo-RCT with 29 patients [36] had low methodological quality with a moderate to high risk of bias. Furthermore, the primary endpoint of the pseudo-RCT, which used an alternating assignation method, was the difference between listening to major key or minor key music during AC [36], which was not our focus in this SR. Eleven studies were performed during a large time-scale of more than six years [24,31,35,37,38,42,43,47,55,57,59], and even 18 years [34]. It is likely, that the findings were strongly affected by a learning curve of the whole team involved in conduction of AC. This implies also the anaesthetic techniques, which were subjects to change. Of note, outcome assessment differed significantly between the studies. Our inclusion of small studies ≤20 patients [19,23,28,39,54,60], bears the risk of overestimation of beneficial outcomes, due to random chance [79]. Furthermore, the estimated treatment-effect tends to be larger in non-randomised studies [80]. Our pre-described outcome variables were not reported in each of the identified studies and hindered therefore a meta-analysis of more than five outcome variables. Inclusion of observational studies into our meta-analysis was justified by the absence of better evidence for the different anaesthetic AC techniques, presently. Concurrently, a sensitivity analysis could only be performed by inclusion of these observational studies, despite the present high-risk of bias in them. Furthermore, we have excluded studies, which were performed outside the operating room or with the use of an intraoperative MRI guidance. This decision was made due to the limited generalizability of these techniques to many hospitals, which do not have a complex infrastructure and the potentially prolonged surgery time by using them. In addition, it has to be acknowledged that the neurological outcome measures and the detection of intraoperative seizures may have differed between the studies.

### Conclusion

SAS and MAC technique for AC seem to be similarly safe without serious complications, whereas evidence for the AAA technique is limited. AC requires a multidisciplinary teamwork and personal experience. The anaesthesiologist has to be skilled in multiple areas, including local anaesthesia for RSNB, advanced airway management, dedicated sedation protocols, an exquisite management of haemodynamics and a high rapid alert to treat possible intraoperative adverse events. AC can be conducted safely even in patients older than 65 years. The neurological outcome can be preserved and even improved in patients undergoing AC. A consequently performed local anaesthesia and scalp nerve block reduces the requirement of sedative agents and postoperative pain. The additionally use of dexmedetomidine enables further reduction of opioid and propofol infusion, while preserving haemodynamic stability. The benefit of MAC and AAA technique consists of reduction/ waiving of sedatives, which probably improves the intraoperative brain mapping. Large RCTs with a standardised protocol are required to prove if there is a significant superiority of one of the three anaesthetic regimes for AC.

## Supporting Information

S1 Checklist Prisma Checklist. (PDF)

S1 Fig Forrest plot of the composite outcome. The summary value is an overall estimate from a random-effect model. The vertical dotted line shows an overall estimate of outcome proportion (based on the meta-analysis) disregarding grouping by technique. Of note, Souter et al. [60] have used both anaesthesia techniques. The composite outcome comprised the outcomes: awake craniotomy failure, intraoperative seizures and mortality within 30 days of surgery.
(TIF)

S2 Fig Comparison between all and prospective studies. The figure shows the predicted proportions for each outcome. The left panels depict results for all studies, and right panels show results for prospective studies only. Of note there is no estimate for new neurological dysfunctions in the SAS group among prospective studies, because only one study provided data.
(TIF)

S1 File EMBASE and PubMed search strategy. (PDF)

S2 File Results of general considerations for AC. (PDF)

S1 Table Patient characteristics. HGG, high grade glioma; LGG, low grade glioma; NK, not known; SD, standard deviation.
(PDF)

S2 Table Risk of bias assessed with the Cochrane Collaboration’s risk of bias tool. +, high risk; -, low risk;?, unknown risk (PDF)

S3 Table Risk of bias according to Agency of Healthcare Research and Quality tool [12]. AC, awake craniotomy; BIS, bispectral index; CT, computed tomography; MMSE, mini-mental state examination; MRI, magnetic resonance imaging; PONV, postoperative nausea and vomiting; VAS, visual analogue scale.
(PDF)

## References

[1] July J, Manninen P, Lai J, Yao Z, Bernstein M. The history of awake craniotomy for brain tumor and its spread into Asia. Surg Neurol. 2009;71: 621–4; discussion 624–5 10.1016/j.surneu.2007.12.022 18452979

[2] Sanai N, Mirzadeh Z, Berger MS. Functional outcome after language mapping for glioma resection. N Engl J Med. 2008;358: 18–27 10.1056/NEJMoa067819 18172171

[3] Caulo M, Briganti C, Mattei PA, Perfetti B, Ferretti A, Romani GL, et al New morphologic variants of the hand motor cortex as seen with MR imaging in a large study population. AJNR Am J Neuroradiol. 2007;28: 1480–1485 17846195 10.3174/ajnr.A0597 PMC8134386

[4] Surbeck W, Hildebrandt G, Duffau H. The evolution of brain surgery on awake patients. Acta Neurochir (Wien). 2015;157: 77–84 25352088 10.1007/s00701-014-2249-8

[5] Brown T, Shah AH, Bregy A, Shah NH, Thambuswamy M, Barbarite E, et al Awake craniotomy for brain tumor resection: the rule rather than the exception? J Neurosurg Anesthesiol. 2013;25: 240–247 10.1097/ANA.0b013e318290c230 23603885

[6] Mallet L, Polosan M, Jaafari N, Baup N, Welter M-L, Fontaine D, et al Subthalamic nucleus stimulation in severe obsessive-compulsive disorder. N Engl J Med. 2008;359: 2121–2134 10.1056/NEJMoa0708514 19005196

[7] Bilotta F, Rosa G. 'Anesthesia' for awake neurosurgery. Curr Opin Anaesthesiol. 2009;22: 560–565 10.1097/ACO.0b013e3283302339 19623055

[8] Ghisi D, Fanelli A, Tosi M, Nuzzi M, Fanelli G. Monitored anesthesia care. Minerva Anestesiol. 2005;71: 533–538 16166913

[9] Liberati A, Altman DG, Tetzlaff J, Mulrow C, Gøtzsche PC, Ioannidis JPA, et al The PRISMA statement for reporting systematic reviews and meta-analyses of studies that evaluate health care interventions: explanation and elaboration. J Clin Epidemiol. 2009;62: e1–34 19631507 10.1016/j.jclinepi.2009.06.006

[10] Bilotta F, Stazi E, Titi L, Lalli D, Delfini R, Santoro A, et al Diagnostic work up for language testing in patients undergoing awake craniotomy for brain lesions in language areas. Br J Neurosurg. 2014;28: 363–367 10.3109/02688697.2013.854313 24195669

[11] Higgins JP, Green S (2008) Cochrane handbook for systematic reviews of interventions Wiley Online Library.

[12] Viswanathan M, Berkman ND, Dryden DM, Hartling L (2013) Assessing Risk of Bias and Confounding in Observational Studies of Interventions or Exposures: Further Development of the RTI Item Bank Rockville (MD): Agency for Healthcare Research and Quality (US). 24006553

[13] Viechtbauer W Conducting meta-analyses in R with the metafor package. Journal of Statistical Software. 2010;36: 1–48

[14] Tramèr MR, Reynolds DJ, Moore RA, McQuay HJ. Impact of covert duplicate publication on meta-analysis: a case study. BMJ. 1997;315: 635–640 9310564 10.1136/bmj.315.7109.635 PMC2127450

[15] von Elm E, Poglia G, Walder B, Tramèr MR. Different patterns of duplicate publication: an analysis of articles used in systematic reviews. JAMA. 2004;291: 974–980 14982913 10.1001/jama.291.8.974

[16] Computing S. R Foundation for Statistical Computing. Vienna, Austria. Salton, G; 1991.

[17] Abdou SA, Shehab HA, Samir EM, Eissa EM. Preliminary evaluation of ketofol-based sedation for awake craniotomy procedures. Egyptian Journal of Anaesthesia. 2010;26: 293–297

[18] Ali MZ, Fadel NA, Abouldahab HA. Awake craniotomy versus general anesthesia for managing eloquent cortex low-grade gliomas. Neurosciences (Riyadh). 2009;14: 263–272 21048628

[19] de Amorim RLO, de Almeida AN, de Aguiar PHP, Fonoff ET, Itshak S, Fuentes D, et al Cortical stimulation of language fields under local anesthesia: optimizing removal of brain lesions adjacent to speech areas. Arq Neuropsiquiatr. 2008;66: 534–538 18813714 10.1590/s0004-282x2008000400018

[20] Andersen JH, Olsen KS. Anaesthesia for awake craniotomy is safe and well-tolerated. Dan Med Bull. 2010;57: A4194 21040682

[21] Beez T, Boge K, Wager M, Whittle I, Fontaine D, Spena G, et al Tolerance of awake surgery for glioma: a prospective European Low Grade Glioma Network multicenter study. Acta Neurochir (Wien). 2013;155: 1301–1308 23689968 10.1007/s00701-013-1759-0

[22] Boetto J, Bertram L, Moulinié G, Herbet G, Moritz-Gasser S, Duffau H. Low Rate of Intraoperative Seizures During Awake Craniotomy in a Prospective Cohort with 374 Supratentorial Brain Lesions: Electrocorticography Is Not Mandatory. World Neurosurg. 2015;84: 1838–1844 10.1016/j.wneu.2015.07.075 26283485

[23] Cai T, Gao P, Shen Q, di Zhang Z, Yao Y, Ji Q. Oesophageal naso-pharyngeal catheter use for airway management in patients for awake craniotomy. Br J Neurosurg. 2013;27: 396–397 10.3109/02688697.2012.743969 23171147

[24] Chacko AG, Thomas SG, Babu KS, Daniel RT, Chacko G, Prabhu K, et al Awake craniotomy and electrophysiological mapping for eloquent area tumours. Clin Neurol Neurosurg. 2013;115: 329–334 10.1016/j.clineuro.2012.10.022 23177182

[25] Chaki T, Sugino S, Janicki PK, Ishioka Y, Hatakeyama Y, Hayase T, et al Efficacy and Safety of a Lidocaine and Ropivacaine Mixture for Scalp Nerve Block and Local Infiltration Anesthesia in Patients Undergoing Awake Craniotomy. J Neurosurg Anesthesiol. 2014; 10.1097/ANA.0000000000000149 25493926

[26] Conte V, L'Acqua C, Rotelli S, Stocchetti N. Bispectral index during asleep-awake craniotomies. J Neurosurg Anesthesiol. 2013;25: 279–284 10.1097/ANA.0b013e3182913afd 23603886

[27] Deras P, Moulinié G, Maldonado IL, Moritz-Gasser S, Duffau H, Bertram L. Intermittent general anesthesia with controlled ventilation for asleep-awake-asleep brain surgery: a prospective series of 140 gliomas in eloquent areas. Neurosurgery. 2012;71: 764–771 22989957 10.1227/NEU.0b013e3182647ab8

[28] Garavaglia MM, Das S, Cusimano MD, Crescini C, Mazer CD, Hare GMT, et al Anesthetic approach to high-risk patients and prolonged awake craniotomy using dexmedetomidine and scalp block. J Neurosurg Anesthesiol. 2014;26: 226–233 10.1097/ANA.0b013e3182a58aba 24064713

[29] Gonen T, Grossman R, Sitt R, Nossek E, Yanaki R, Cagnano E, et al Tumor location and IDH1 mutation may predict intraoperative seizures during awake craniotomy. J Neurosurg. 2014;121: 1133–1138 10.3171/2014.7.JNS132657 25170661

[30] Grossman R, Ram Z, Perel A, Yusim Y, Zaslansky R, Berkenstadt H. Control of postoperative pain after awake craniotomy with local intradermal analgesia and metamizol. Isr Med Assoc J. 2007;9: 380–382 17591378

[31] Grossman R, Nossek E, Sitt R, Hayat D, Shahar T, Barzilai O, et al Outcome of elderly patients undergoing awake-craniotomy for tumor resection. Ann Surg Oncol. 2013;20: 1722–1728 10.1245/s10434-012-2748-x 23212761

[32] Gupta DK, Chandra PS, Ojha BK, Sharma BS, Mahapatra AK, Mehta VS. Awake craniotomy versus surgery under general anesthesia for resection of intrinsic lesions of eloquent cortex—a prospective randomised study. Clin Neurol Neurosurg. 2007;109: 335–343 17303322 10.1016/j.clineuro.2007.01.008

[33] Hansen E, Seemann M, Zech N, Doenitz C, Luerding R, Brawanski A. Awake craniotomies without any sedation: the awake-awake-awake technique. Acta Neurochir (Wien). 2013;155: 1417–1424 23812965 10.1007/s00701-013-1801-2

[34] Hervey-Jumper SL, Li J, Lau D, Molinaro AM, Perry DW, Meng L, et al Awake craniotomy to maximize glioma resection: methods and technical nuances over a 27-year period. J Neurosurg. 2015;123: 325–339 10.3171/2014.10.JNS141520 25909573

[35] Ilmberger J, Ruge M, Kreth F-W, Briegel J, Reulen H-J, Tonn J-C. Intraoperative mapping of language functions: a longitudinal neurolinguistic analysis. J Neurosurg. 2008;109: 583–592 10.3171/JNS/2008/109/10/0583 18826344

[36] Jadavji-Mithani R, Venkatraghavan L, Bernstein M. Music is Beneficial for Awake Craniotomy Patients: A Qualitative Study. Can J Neurol Sci. 2015;42: 7–16 10.1017/cjn.2014.127 25635400

[37] Kim SS, McCutcheon IE, Suki D, Weinberg JS, Sawaya R, Lang FF, et al Awake craniotomy for brain tumors near eloquent cortex: correlation of intraoperative cortical mapping with neurological outcomes in 309 consecutive patients. Neurosurgery. 2009;64: 836–45; discussion 345–6 10.1227/01.NEU.0000342405.80881.81 19404147

[38] Li T, Bai H, Wang G, Wang W, Lin J, Gao H, et al Glioma localization and excision using direct electrical stimulation for language mapping during awake surgery. Exp Ther Med. 2015;9: 1962–1966 26136923 10.3892/etm.2015.2359 PMC4471693

[39] Lobo F, Beiras A. Propofol and remifentanil effect-site concentrations estimated by pharmacokinetic simulation and bispectral index monitoring during craniotomy with intraoperative awakening for brain tumor resection. J Neurosurg Anesthesiol. 2007;19: 183–189 17592350 10.1097/ANA.0b013e31805f66ad

[40] Low D, Ng I, Ng W-H. Awake craniotomy under local anaesthesia and monitored conscious sedation for resection of brain tumours in eloquent cortex—outcomes in 20 patients. Ann Acad Med Singapore. 2007;36: 326–331 17549278

[41] McNicholas E, Bilotta F, Titi L, Chandler J, Rosa G, Koht A. Transient facial nerve palsy after auriculotemporal nerve block in awake craniotomy patients. A A Case Rep. 2014;2: 40–43 10.1097/ACC.0b013e3182a8ee71 25611249

[42] Nossek E, Matot I, Shahar T, Barzilai O, Rapoport Y, Gonen T, et al Failed awake craniotomy: a retrospective analysis in 424 patients undergoing craniotomy for brain tumor. J Neurosurg. 2013;118: 243–249 10.3171/2012.10.JNS12511 23121432

[43] Nossek E, Matot I, Shahar T, Barzilai O, Rapoport Y, Gonen T, et al Intraoperative seizures during awake craniotomy: incidence and consequences: analysis of 477 patients. Neurosurgery. 2013;73: 135–40; discussion 140 10.1227/01.neu.0000429847.91707.97 23615101

[44] Olsen KS The asleep-awake technique using propofol-remifentanil anaesthesia for awake craniotomy for cerebral tumours. Eur J Anaesthesiol. 2008;25: 662–669 10.1017/S0265021508003633 18289443

[45] Ouyang MW, McDonagh DL, Phillips-Bute B, James ML, Friedman AH, Gan TJ. Does midline shift predict postoperative nausea in brain tumor patients undergoing awake craniotomy? A retrospective analysis. Curr Med Res Opin. 2013;29: 1033–1038 10.1185/03007995.2013.811071 23731200

[46] Ouyang MW, McDonagh DL, Phillips-Bute B, James ML, Friedman AH, Gan TJ. Comparison of postoperative nausea between benign and malignant brain tumor patients undergoing awake craniotomy: a retrospective analysis. Curr Med Res Opin. 2013;29: 1039–1044 10.1185/03007995.2013.811070 23731201

[47] Pereira LCM, Oliveira KM, L'Abbate GL, Sugai R, Ferreira JA, da Motta LA. Outcome of fully awake craniotomy for lesions near the eloquent cortex: analysis of a prospective surgical series of 79 supratentorial primary brain tumors with long follow-up. Acta Neurochir (Wien). 2009;151: 1215–1230 19730779 10.1007/s00701-009-0363-9

[48] Peruzzi P, Bergese SD, Viloria A, Puente EG, Abdel-Rasoul M, Chiocca EA. A retrospective cohort-matched comparison of conscious sedation versus general anesthesia for supratentorial glioma resection. Clinical article. J Neurosurg. 2011;114: 633–639 10.3171/2010.5.JNS1041 20560720 PMC4256674

[49] Pinsker MO, Nabavi A, Mehdorn HM. Neuronavigation and resection of lesions located in eloquent brain areas under local anesthesia and neuropsychological-neurophysiological monitoring. Minim Invasive Neurosurg. 2007;50: 281–284 18058644 10.1055/s-2007-985825

[50] Rajan S, Cata JP, Nada E, Weil R, Pal R, Avitsian R. Asleep-awake-asleep craniotomy: a comparison with general anesthesia for resection of supratentorial tumors. J Clin Neurosci. 2013;20: 1068–1073 10.1016/j.jocn.2012.09.031 23453156

[51] Rughani AI, Rintel T, Desai R, Cushing DA, Florman JE. Development of a safe and pragmatic awake craniotomy program at Maine Medical Center. J Neurosurg Anesthesiol. 2011;23: 18–24 10.1097/ANA.0b013e3181ebf050 20706142

[52] Sacko O, Lauwers-Cances V, Brauge D, Sesay M, Brenner A, Roux F-E. Awake craniotomy vs surgery under general anesthesia for resection of supratentorial lesions. Neurosurgery. 2011;68: 1192–8; discussion 1198–9 10.1227/NEU.0b013e31820c02a3 21273923

[53] Sanus GZ, Yuksel O, Tunali Y, Ozkara C, Yeni N, Ozlen F, et al Surgical and anesthesiological considerations of awake craniotomy: Cerrahpasa experience. Turk Neurosurg. 2015;25: 210–217 10.5137/1019-5149.JTN.8176-13.1 26014002

[54] See J-J, Lew TWK, Kwek T-K, Chin K-J, Wong MFM, Liew Q-Y, et al Anaesthetic management of awake craniotomy for tumour resection. Ann Acad Med Singapore. 2007;36: 319–325 17549277

[55] Serletis D, Bernstein M. Prospective study of awake craniotomy used routinely and nonselectively for supratentorial tumors. J Neurosurg. 2007;107: 1–6 10.3171/JNS-07/07/0001 17639865

[56] Shen S-L, Zheng J-Y, Zhang J, Wang W-Y, Jin T, Zhu J, et al Comparison of dexmedetomidine and propofol for conscious sedation in awake craniotomy: a prospective, double-blind, randomized, and controlled clinical trial. Ann Pharmacother. 2013;47: 1391–1399 10.1177/1060028013504082 24259599

[57] Shinoura N, Midorikawa A, Yamada R, Hana T, Saito A, Hiromitsu K, et al Awake craniotomy for brain lesions within and near the primary motor area: A retrospective analysis of factors associated with worsened paresis in 102 consecutive patients. Surg Neurol Int. 2013;4: 149 10.4103/2152-7806.122003 24381792 PMC3872643

[58] Sinha PK, Koshy T, Gayatri P, Smitha V, Abraham M, Rathod RC. Anesthesia for awake craniotomy: a retrospective study. Neurol India. 2007;55: 376–381 18040111 10.4103/0028-3886.33308

[59] Sokhal N, Rath GP, Chaturvedi A, Dash HH, Bithal PK, Chandra PS. Anaesthesia for awake craniotomy: A retrospective study of 54 cases. Indian J Anaesth. 2015;59: 300–305 10.4103/0019-5049.156878 26019355 PMC4445152

[60] Souter MJ, Rozet I, Ojemann JG, Souter KJ, Holmes MD, Lee L, et al Dexmedetomidine sedation during awake craniotomy for seizure resection: effects on electrocorticography. J Neurosurg Anesthesiol. 2007;19: 38–44 17198099 10.1097/01.ana.0000211027.26550.24

[61] Wrede KH, Stieglitz LH, Fiferna A, Karst M, Gerganov VM, Samii M, et al Patient acceptance of awake craniotomy. Clin Neurol Neurosurg. 2011;113: 880–884 10.1016/j.clineuro.2011.06.010 21782320

[62] Zhang Z, Jiang T, Xie J, Liu F-S, Li S-W, Qiao H, et al Surgical strategies for glioma involving language areas. Chin Med J (Engl). 2008;121: 1800–1805 19080361

[63] Wood J Methodology for dealing with duplicate study effects in a meta-analysis. Organizational Research Methods. 2008;

[64] Bekker AY, Kaufman B, Samir H, Doyle W. The use of dexmedetomidine infusion for awake craniotomy. Anesth Analg. 2001;92: 1251–1253 11323355 10.1097/00000539-200105000-00031

[65] McGirt MJ, Chaichana KL, Gathinji M, Attenello FJ, Than K, Olivi A, et al Independent association of extent of resection with survival in patients with malignant brain astrocytoma. J Neurosurg. 2009;110: 156–162 10.3171/2008.4.17536 18847342

[66] Yamaguchi S, Kobayashi H, Terasaka S, Ishii N, Ikeda J, Kanno H, et al The impact of extent of resection and histological subtype on the outcome of adult patients with high-grade gliomas. Jpn J Clin Oncol. 2012;42: 270–277 10.1093/jjco/hys016 22399670

[67] Guilfoyle MR, Helmy A, Duane D, Hutchinson PJA. Regional scalp block for postcraniotomy analgesia: a systematic review and meta-analysis. Anesth Analg. 2013;116: 1093–1102 10.1213/ANE.0b013e3182863c22 23477962

[68] Osborn I, Sebeo J. "Scalp block" during craniotomy: a classic technique revisited. J Neurosurg Anesthesiol. 2010;22: 187–194 10.1097/ANA.0b013e3181d48846 20479675

[69] Bilotta F, Titi L, Lanni F, Stazi E, Rosa G. Training anesthesiology residents in providing anesthesia for awake craniotomy: learning curves and estimate of needed case load. J Clin Anesth. 2013;25: 359–366 10.1016/j.jclinane.2013.01.012 23965201

[70] Fabling JM, Gan TJ, El-Moalem HE, Warner DS, Borel CO. A randomized, double-blinded comparison of ondansetron, droperidol, and placebo for prevention of postoperative nausea and vomiting after supratentorial craniotomy. Anesth Analg. 2000;91: 358–361 10910848 10.1097/00000539-200008000-00023

[71] Pemberton PL, Dinsmore J. Bispectral index monitoring during awake craniotomy surgery. Anaesthesia. 2002;57: 1244–1245

[72] De Sloovere V, De Deyne C, Wuyts J, and Heylen R. Bispectral index monitoring during asleep-awake technique for craniotomy. Eur J Anaesthesiol. 2009;26: 443–444. 10.1097/EJA.0b013e32831bc70c 19521303

[73] Ott C, Kerscher C, Luerding R, Doenitz C, Hoehne J, Zech N, et al The impact of sedation on brain mapping: a prospective, interdisciplinary, clinical trial. Neurosurgery. 2014;75: 117–23; discussion 123; quiz 123 10.1227/NEU.0000000000000359 24691469

[74] Dahyot-Fizelier C, Frasca D, Debaene B. [Inhaled agents in neuroanaesthesia for intracranial surgery: pro or con]. Ann Fr Anesth Reanim. 2012;31: e229–e234 10.1016/j.annfar.2012.08.003 22995641

[75] Sartorius CJ, Berger MS. Rapid termination of intraoperative stimulation-evoked seizures with application of cold Ringer's lactate to the cortex. Technical note. J Neurosurg. 1998;88: 349–351 9452250 10.3171/jns.1998.88.2.0349

[76] Sartorius CJ, Wright G. Intraoperative brain mapping in a community setting—technical considerations. Surg Neurol. 1997;47: 380–388 9122843 10.1016/s0090-3019(96)00340-0

[77] Carrabba G, Venkatraghavan L, Bernstein M. Day surgery awake craniotomy for removing brain tumours: technical note describing a simple protocol. Minim Invasive Neurosurg. 2008;51: 208–210 10.1055/s-2008-1073132 18683111

[78] von Elm E, Altman DG, Egger M, Pocock SJ, Gøtzsche PC, Vandenbroucke JP, et al The Strengthening the Reporting of Observational Studies in Epidemiology (STROBE) statement: guidelines for reporting observational studies. PLoS Med. 2007;4: e296 17941714 10.1371/journal.pmed.0040296 PMC2020495

[79] Moore RA, Gavaghan D, Tramèr MR, Collins SL, McQuay HJ. Size is everything—large amounts of information are needed to overcome random effects in estimating direction and magnitude of treatment effects. Pain. 1998;78: 209–216 9870574 10.1016/S0304-3959(98)00140-7

[80] Deeks JJ, Dinnes J, D'Amico R, Sowden AJ, Sakarovitch C, Song F, et al Evaluating non-randomised intervention studies. Health Technol Assess. 2003;7: iii–x, 1–173. 10.3310/hta7270 14499048

