# Supplementary figures and images for: Anaesthesia Management for Awake Craniotomy: Systematic Review and Meta-Analysis
Source: PLoS One. 2016 May 26;11(5):e0156448. doi: 10.1371/journal.pone.0156448 (PMC4882028; doi:10.1371/journal.pone.0156448)

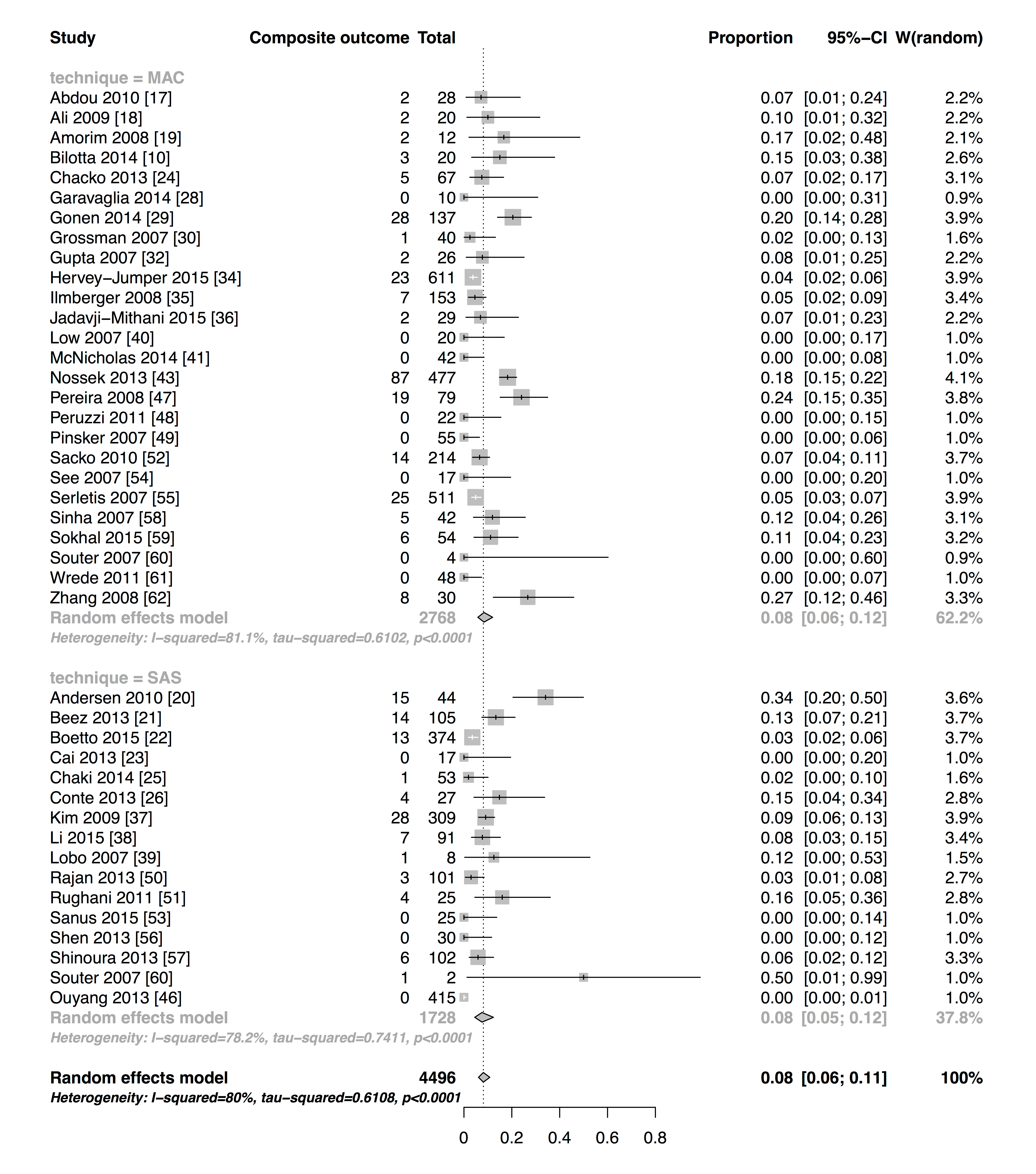

Supplement: S1 Fig — The summary value is an overall estimate from a random-effect model. The vertical dotted line shows an overall estimate of outcome proportion (based on the meta-analysis) disregarding grouping by technique. Of note, Souter et al. [60] have used both anaesthesia techniques. The composite outcome comprised the outcomes: awake craniotomy failure, intraoperative seizures and mortality within 30 days of surgery. (TIF) [file pone.0156448.s002.tif]

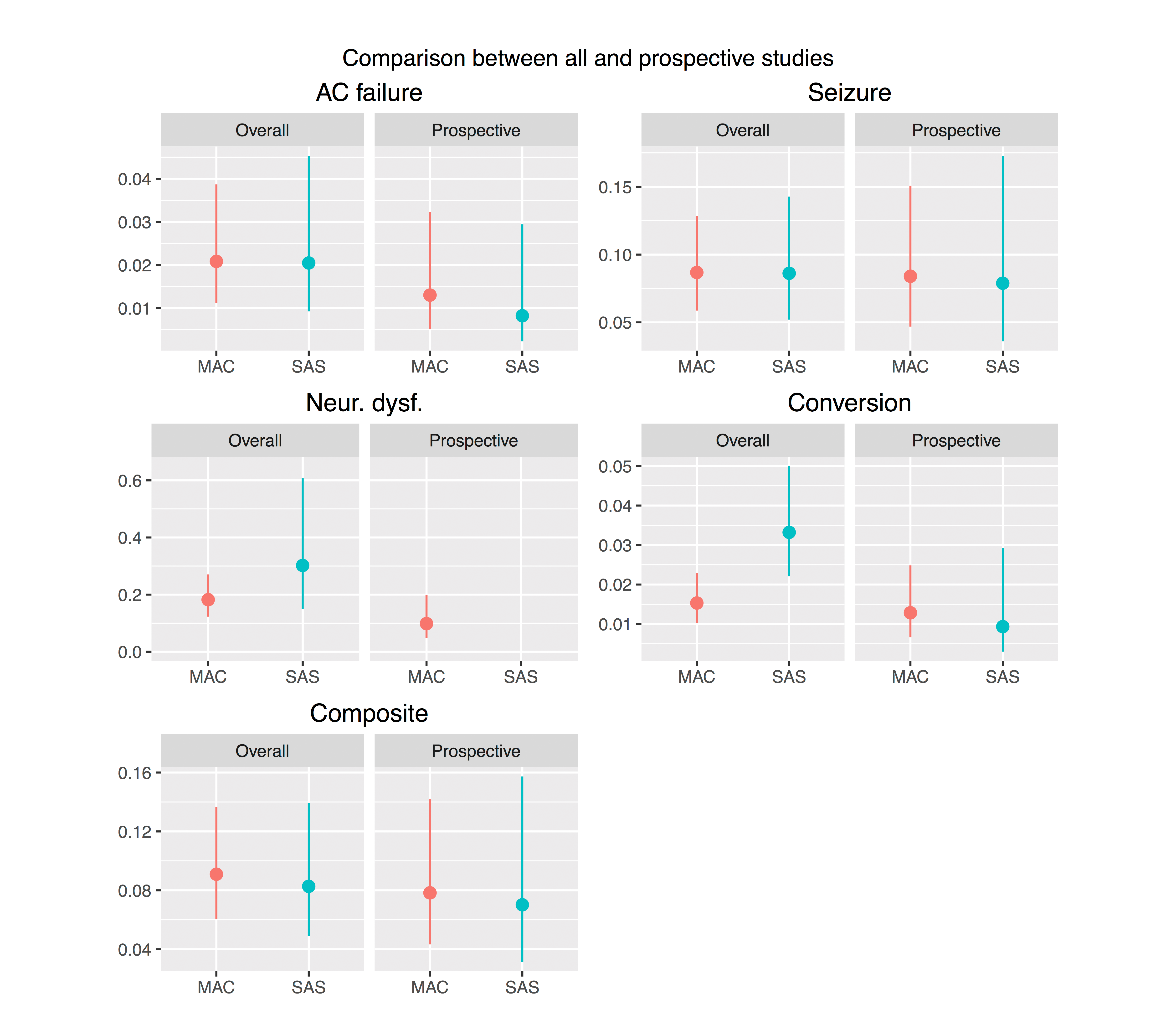

Supplement: S2 Fig — The figure shows the predicted proportions for each outcome. The left panels depict results for all studies, and right panels show results for prospective studies only. Of note there is no estimate for new neurological dysfunctions in the SAS group among prospective studies, because only one study provided data. (TIF) [file pone.0156448.s003.tif]
