# Supplementary material for: Anaesthesia Management for Awake Craniotomy: Systematic Review and Meta-Analysis
Source: PLoS One. 2016 May 26;11(5):e0156448. doi: 10.1371/journal.pone.0156448 (PMC4882028; doi:10.1371/journal.pone.0156448)
Supplement: S1 File — (PDF) [file pone.0156448.s004.pdf]

## S1 File. EMBASE and PubMed search strategy.

### EMBASE

The following search term was used for the EMBASE database: “((awake and an?sthesia and craniotom?) not child?)” OR “((awake and craniotom?) not child?)” OR “((awake an?sthesia and techni?) not child?)” OR “((selective and scalp and nerve and block) and craniotom?)”. The following filter were additionally applied: "AND PY=2007 to 2015 AND pps=human".

### PubMed

1. (awake[All Fields] AND ("craniotomy"[MeSH Terms] OR "craniotomy"[All Fields])) AND ("2007/01/01"[PDAT] : "2015/12/31"[PDAT])
2. awake[All Fields] AND ("anaesthesia"[All Fields] OR "anesthesia"[MeSH Terms] OR "anesthesia"[All Fields]) AND ("craniotomy"[MeSH Terms] OR "craniotomy"[All Fields])
3. (awake[All Fields] AND ("anaesthesia"[All Fields] OR "anesthesia"[MeSH Terms] OR "anesthesia"[All Fields]) AND technique[All Fields]) AND ("2007/01/01"[PDAT] : "2015/12/31"[PDAT])
4. (selective[All Fields] AND ("scalp"[MeSH Terms] OR "scalp"[All Fields]) AND ("nerve tissue"[MeSH Terms] OR ("nerve"[All Fields] AND "tissue"[All Fields]) OR "nerve tissue"[All Fields] OR "nerve"[All Fields]) AND block[All Fields] AND ("craniotomy"[MeSH Terms] OR "craniotomy"[All Fields])) AND ("2007/01/01"[PDAT] : "2015/12/31"[PDAT])
