# Supplementary material for: Anaesthesia Management for Awake Craniotomy: Systematic Review and Meta-Analysis
Source: PLoS One. 2016 May 26;11(5):e0156448. doi: 10.1371/journal.pone.0156448 (PMC4882028; doi:10.1371/journal.pone.0156448)
Supplement: S2 File — (PDF) [file pone.0156448.s005.pdf]

S2 File. Results of general considerations for AC.

Pre-operative patient selection and preparation

Accurate patient selection is a crucial point for successful AC. The patient inclusion criteria in the analysed studies showed a huge variation. While some studies excluded patients with obesity, reflux, obstructive sleep apnoea syndrome (OSAS), cardiovascular or pulmonary disease, difficult airway, anxiety, poor preoperative functional status (like pre-existing paralysis, severe aphasia, or severe cognitive dysfunction) and psychiatric disorders [10,17,18,27,33,35,36,38,42,47,53,56,59], others enabled AC surgery also to higher risk patients. Patients with preoperative midline shift, or signs of increased intracranial pressure, were successfully treated in four studies [28,34,45,55]. Patients with chronic obstructive pulmonary disease (COPD) or chronic chough, congestive heart failure, obesity, OSAS, anticipated difficult airway, anxiety with panic attacks, and depression underwent AC in two studies [28,34]. Nossek et al. [42] included also cooperative patients with preoperative language dysfunction, justified by the importance to preserve at least some language function. Psychological preparation of the patients is another important issue for a successful AC conduction. This can be achieved by a pre-operative personal contact of the attending anaesthesiologist [10], a social worker [31], a neurologist or neuropsychologist [24], or a neurosurgical team [21]. Hereby the patient should be explained in detail the course of the awake surgery, the environment of the surgery room including the upcoming noises, the intraoperative testing procedure and potential adverse events. The aim is to attenuate patients` anxiety and to build up confidence.

Premedication

The most studies applied around 1-2 mg midazolam intravenously as premedication in all [10,17,26,29,31,34,39,42,43,51,53], respectively some of their patients [20,36,44,54]. Only five studies stated clearly that midazolam was not used [32,56,58-60], the remaining did not report it.

Other prophylactic drugs

Intraoperative nausea is accompanied by the risk of vomiting with consecutive increase of intracranial pressure and potential aspiration. Furthermore, PONV has a significant impact on the patients` satisfaction [71]. Some studies reported the prophylactic antiemetic application of ondansetron, dolasetron, metoclopramide, dexamethasone and betamethasone [17,18,22,25-28,33,34,37,39,40,44-46,51,58,59]. Interestingly, Sinha et al. used also haloperidol for the antiemetic purpose [58], despite its potential to induce neurologic adverse effects. Prophylaxis of brain oedema by application of corticosteroids and mannitol at the beginning of surgery was also performed in some studies routinely [17,20,40,44,51-55,59].

Anaesthesia management

Monitoring

Standard monitoring consisted in all included studies of electrocardiogram, pulse oximetry, capnometry, temperature and invasive blood pressure monitoring. Urinary catheters were routinely inserted in the most studies. Waiving of urinary catheter induced discomfort by urine holding in one study [38]. Interestingly, only one study used an intraoperative intra cranial pressure (ICP) monitoring, by placing an intraparenchymal ICP sensor [17].

Regional scalp nerve block (RSNB)

Scalp nerve blocks are an established technique that involves infiltration of local anaesthetic at well-defined anatomical sites targeting the major sensory innervation of the scalp. Independent of the used anaesthesia technique, a RSNB and/ or local anaesthesia to head fixation pin sites and the skin incision area were performed except in one study, where it was not explicitly mentioned [23]. The applied local anaesthetics showed a huge variation. Most studies used solely ropivacaine [10,33,37-39,41,56,62], or bupivacaine [28,32,35,38,40,41,44,47,49,51,55,59]. Ropivacaine was also used in combination with lidocaine [25], or mepivacaine [26],

others used bupivacaine combined with lidocaine [18,19,24,30,34,54,58,60], thirteen patients in the study of McNicholas et al. [29] received a combination of lidocaine and tetracaine. Furthermore, epinephrine was inconsistently added to the anaesthetics.

Anaesthesia depth control

Consciousness was measured in only sixteen studies: clinically by Richmond agitation sedation score (RASS) [10,28] and the Observer's assessment of alertness/ sedation (OAA/S) scale [17,48,60], objectively by Bispectral Index (BIS) [23,26,33,39,48,53,58,59,62] and by the Response Entropy Index (RE) [56,59].
