# Supplementary material for: Anaesthesia Management for Awake Craniotomy: Systematic Review and Meta-Analysis
Source: PLoS One. 2016 May 26;11(5):e0156448. doi: 10.1371/journal.pone.0156448 (PMC4882028; doi:10.1371/journal.pone.0156448)
Supplement: S1 Table — HGG, high grade glioma; LGG, low grade glioma; NK, not known; SD, standard deviation. (PDF) [file pone.0156448.s006.pdf]

**S1 Table. Patient characteristics.**

| Study | Male | Female | Mean age in years [range] or ±SD | Kind of tumour description |
|---|---|---|---|---|
| Abdou 2010 [17] | 19 | 9 | 33.4 ± 9.3 [21–56] | NK |
| Ali 2009 [18] | 15 | 5 | 49 ± 7.44 [23-55] | 20 LGG |
| Amorim 2008 [19] | 6 | 6 | 35.4 [14-52] | 6 LGG, 1 HGG, 3 cavernoma, 2 other |
| Andersen 2010 [20] | 29 | 15 | 43 [19-69] | NK |
| Beez 2013 [21] | 57 | 48 | 46 [18-64] | 47 LGG, 58 HGG |
| Bilotta 2014 [10] | 8 | 12 | 56 [34-77] | 15 glioma, 2 meningioma, 3 metastases |
| Boetto 2015 [22] | 205 | 169 | 39.5 ± 10.7 | 329 LGG, 36 HGG, 5 cavernoma, 4 metastases |
| Cai 2013 [23] | 14 | 3 | [15-41] | NK, only described: tumours in eloquent area |
| Chacko 2013 [24] | 55 | 12 | median 34.6 [13–58] | Glioma |
| Chaki 2014 [25] | 26 | 27 | 48±18 | 48 brain tumours, 2 epilepsy, 2 intracranial aneurysm, 1 haemangioma |
| Conte 2013 [26] | 15 | 12 | 39 [19-70] | 25 glioma, 1 metastasis, 1 angioma |
| Deras 2012 [27] | 92 | 48 | 39.5 ± 10 | Glioma |
| Garavaglia 2014 [28] | 6 | 4 | 55,7 [29-79] | 2 LGG, 3 HGG, 5 metastases |
| Gonen 2014 [29] | 80 | 57 | 47,8 ± 17,2 | 36 LGG, 77 HGG, 17 metastases, 6 other |
| Grossman 2007 [30] | NK | NK | NK | NK (only brain tumours described) |
| Grossman 2013 [31] | 238 (188 young + 50 elderly) | 186 (146 young + 40 elderly) | young: 45.4±13.2 elderly: 71.7±5.1 | 81 LGG, 236 HGG, 87 metastases |

| | | | | |
|---|---|---|---|---|
| Gupta 2007 [32] | 20 | 6 | 42.7±15.8 [18-72] | 16 LGG, 8 HGG, 1 metastasis, 1 cavernoma |
| Hansen 2013 [33] | 30 | 17 | NK | 9 LGG, 37 HGG, 3 metastases, 1 cavernoma |
| Hervey-Jumper 2015 [34] | NK | NK | median 42 [13–84] | 259 LGG, 335 HGG, 3 metastases, 14 other |
| Ilmberger 2008 [35] | 70 | 79 | 39 [15-67] | 74 LGG, 62 HGG, 2 metastases, 4 cavernoma, 7 other |
| Jadavji-Mithani 2015 [36] | 15 | 14 | 52 [22-78] | NK (only stated brain tumour or epilepsy) |
| Kim 2009 [37] | 191 | 108 | median 45 [13-75] | 58 LGG, 226 HGG, 20 metastases, 5 other |
| Li 2015 [38] | 52 | 39 | 38.7 | 66 LGG, 25 HGG |
| Lobo 2007 [39] | 5 | 3 | 39.5 ± 3.6 | NK |
| Low 2007 [40] | 7 | 13 | 39.8 [10-73] | 7 LGG, 7 HGG, 4 metastases, 2 other |
| McNicholas 2014 [41] | 21 | 21 | 55±13 [20-83] | NK |
| Nossek 2013 [42] | 238 | 186 | 51.5 ± 16.1 | 80 LGG, 233 HGG, 87 metastases, 24 other |
| Nossek 2013 [43] | 272 | 205 | 51.1 ± 16.3 | 86 LGG, remaining patients not stated |
| Olsen 2008 [44] | NK | NK | 46.2 [29-69] | 16 LGG, 8 HGG, 1 metastasis |
| Ouyang 2013 [45] | 226 | 160 | Midline shift 48±13.2, no midline shift 48.3 ±15 | NK (282 malignant tumours reported) |
| Ouyang 2013 [46] | 247 | 168 | Malignant group 51.1±14.2, benign group mean 40,5±12,9 | 103 LGG, 264 HGG, 28 metastases, 20 other |

| | | | | |
|---|---|---|---|---|
| Pereira 2008 [47] | NK | NK | Group A before 8/2004 39.9 ± 11.1, Group B after 8/2004 38.9 ± 13.7 | Group A (before 8/2004): 17 LGG, 16 HGG; group B (after 8/2004): 24 LGG, 22 HGG |
| Peruzzi 2011 [48] | 15 | 7 | male: 50.7 [36-69], female: 50.5 [ 27-65] | 2 LGG, 20HGG |
| Pinsker 2007 [49] | 37 | 15 | 50.3 | 11 LGG, 41 HGG, 1 cavernoma, 2 metastases |
| Rajan 2013 [50] | 64 | 37 | median 52 [40-65] | NK (99 malignant tumours and 2 benign tumours reported) |
| Rughani 2011 [51] | 13 | 12 | 53.3 [25-77] | 6 LGG, 12 HGG, 1 cavernoma, 2 metastases, 3 meningeoma, 1 other |
| Sacko 2010 [52] | 114 | 100 | median 46.5 | 70 LGG, 73 HGG, 42 metastases, 2 meningeoma, 22 cavernoma, 5 other |
| Sanus 2015 [53] | 17 | 8 | 34.2 | 13 glioma/astrocytoma, 6 cavernoma, 5 others, 1 metastasis |
| See 2007 [54] | 8 | 9 | median 40 [19-74] | 7 LGG, 4 HGG, 3 metastases, 3 others |
| Serletis 2007 [55] | NK | NK | NK | NK |
| Shen 2013 [56] | 11 | 19 | Dexmedetomidine 50.5 ±14.0, propofol 49.5±15.9 | NK (only brain glioma reported) |
| Shinoura 2013 [57] | 55 | 47 | median 61 [range 34-80 years] | |
| Sinha 2007 [58] | 28 | 14 | 38.8±23.6 | 28 LGG, 1 cavernoma, 1 angioma, 12 other |
| Sokhal 2015 [59] | 43 | 11 | 36.1±14.3 [17-72] | NK |

| | | | | |
|---|---|---|---|---|
| Souter 2007 [60] | 2 | 4 | 25.8 ± 14.1 [17-57] | NK |
| Wrede 2011 [61] | 37 | 9 | 45.37 [18-71] | NK |
| Zhang 2008 [62] | 21 | 9 | 38.9 [18–64] | 22 LGG, 8 HGG |

HGG, high grade glioma; LGG, low grade glioma; NK, not known; SD, standard deviation
