# Supplementary material for: Anaesthesia Management for Awake Craniotomy: Systematic Review and Meta-Analysis
Source: PLoS One. 2016 May 26;11(5):e0156448. doi: 10.1371/journal.pone.0156448 (PMC4882028; doi:10.1371/journal.pone.0156448)
Supplement: S2 Table — (PDF) [file pone.0156448.s007.pdf]

Study/ Explanation for judgment	Random sequence generation (selection bias)	Allocation concealment (selection bias)	Blinding of patients + personnel (performance bias)	Blinding of outcome assessment (detection bias)	Incomplete outcome data (attrition bias)	Selective reporting (Reporting bias)	Other bias
**Gupta 2007 [32]**	+	+	+	+	-	?	-
Explanation	Not reported	Not reported	Anaesthesiologists were not blinded	No description of a blinded outcome assessor.	Maximum loss to follow up were 20% for the outcome variable: extent of resection	Study protocol not assessed	Baseline characteristics and surgery performance similar in all patients.
**Jadavji-Mithani 2015 [36]**	**+**	**+**	**+**	**+**	**-**	**?**	**?**
Explanation	Rotary method (alternately assignment)	No concealment for the investigators, as the patients were alternately assigned to the	Only patients were blinded.	No description of a blinded outcome assessor.	All patients included in analysis, no loss to follow up.	Unclear definition of pre-specified outcomes, protocol not assessed	Insufficient information to assess other potential risks of bias. Statistical analysis of baseline characteristics

		music group.					between the two groups is not shown.
**Shen 2013 [56]**	+	+	+	-	-	?	-
Explanation	Not reported	Randomisation list mentioned, but not the concealment process	Anaesthesiologist s were not blinded	Outcome assessor blinded	All data reported, no loss to follow up	Study protocol not assessed	Baseline characteristics and anaesthetic protocols similar in all patients.

S2 Table. Risk of bias assessed with the Cochrane Collaboration's risk of bias tool. +, high risk; **-**, low risk; **?**, unknown risk
