# Supplementary material for: Anaesthesia Management for Awake Craniotomy: Systematic Review and Meta-Analysis
Source: PLoS One. 2016 May 26;11(5):e0156448. doi: 10.1371/journal.pone.0156448 (PMC4882028; doi:10.1371/journal.pone.0156448)
Supplement: S3 Table — AC, awake craniotomy; BIS, bispectral index; CT, computed tomography; MMSE, mini-mental state examination; MRI, magnetic resonance imaging; PONV, postoperative nausea and vomiting; VAS, visual analogue scale. (PDF) [file pone.0156448.s008.pdf]

S3 Table. Risk of bias according to Agency of Healthcare Research and Quality tool [12]. AC, awake craniotomy; BIS, bispectral index; CT, computed tomography; MMSE, mini-mental state examination; MRI, magnetic resonance imaging; PONV, postoperative nausea and vomiting; VAS, visual analogue scale;

***Selection Bias* Q1: Do the inclusion/ exclusion criteria vary across the comparison groups/ respectively the individuals (if only one group) of the study?**					
**Study**	**Yes, varies**	**Partially: some, but not all criteria, applied to all groups or not clearly stated if some criteria are applied to all groups**	**No, does not vary**	**Cannot determine: article does not specify**	**Explanation**
Abdou 2010 [17]			X		Same for all individuals.
Ali 2009 [18]			X		Same for all individuals.
Amorim 2008 [19]				X	Predefined inclusion and exclusion criteria are not stated clearly.
Andersen 2010 [20]				X	Predefined inclusion and exclusion criteria are not stated clearly.
Beez 2013 [21]		X			Some general inclusion and exclusion criteria are stated, but others depended on the "protocol of the individual neurosurgical centre".

Bilotta 2014 [10]	X		Same for all individuals.
Boetto 2015 [22]		X	Predefined inclusion and exclusion criteria are not stated clearly for the decision to perform AC the patients.
Cai 2013 [23]		X	Predefined inclusion and exclusion criteria are not stated.
Chacko 2013 [24]	X		Same for all individuals
Chaki 2014 [25]		X	Predefined inclusion and exclusion criteria are not stated.
Conte 2013 [26]	X		Same for all individuals, as exclusion criteria are not stated and all awake craniotomy patients were consecutively recruited.
Deras 2012 [27]	X		Same for all individuals
Garavaglia 2014 [28]	X		Same for all individuals. Predefined inclusion criteria stated (inclusion of high risk patients).
Gonen 2014 [29]	X		The inclusion and exclusion criteria are not stated clearly, but all primary AC surgeries conducted during the study period were included.
Grossman 2007 [30]		X	Inclusion and exclusion criteria are stated very vague.

Grossman 2013 [31]	X			All patients with supratentorial lesion located within or in proximity to eloquent brain areas between 2003-2010 were included. It has to be expected that the patient selection and pre-operative treatment during this long recruiting period has changed due to a learning curve.
Hansen 2013 [33]		X		Same for all individuals. An interdisciplinary brain tumour board made decision for AC, according to pre-defined criteria.
Hervey-Jumper 2015 [34]		X		Same for all individuals
Ilmberger 2008 [35]		X		Same for all individuals
Kim 2009 [37]		X		Same for all individuals
Li 2015 [38]		X		Same for all individuals
Lobo 2007 [39]		X		Same for all individuals.
Low 2007 [40]			X	Some global inclusion criteria for AC "cooperative and physically able to tolerate awake surgery" are stated, but it remains unclear how the identification of a patient with a tumour in the eloquent area was performed. Furthermore, it is not clear, if there were also AC procedures with other anaesthesia techniques, or if all patients with AC in the predefined time-span received the MAC technique. If there were also other kinds of anaesthesia, what was the indication for MAC?

				Authors only report, that they have included AC procedures, which were performed with MAC technique.
McNicholas 2014 [41]			X	Predefined inclusion and exclusion criteria are not stated.
Nossek 2013 [42]	X			One physician selected all patients. This decision was subjective, but underlied specific inclusion and exclusion criteria, which have changed during the entire study period due to the learning curve of the AC team. A surgeon performed pre-operative speech evaluation until 2009 and thereafter a neurocognitive team took over this role.
Nossek 2013 [43]	X			Preoperative functional magnet resonance imaging was performed in only some patients before AC decision.
Olsen 2008 [44]			X	Predefined inclusion and exclusion criteria are not stated clearly.
Ouyang 2013 [45]		X		Inclusion based on preoperative MRI or CT imaging and the diagnosis of midline shift or no midline shift.
Ouyang 2013 [46]			X	Predefined inclusion and exclusion criteria are not stated.
Pereira 2008 [47]		X		Same for all individuals.
Peruzzi 2011 [48]		X		Same for all individuals
Pinsker 2007 [49]			X	All individuals with AC during the predefined study period were included, but the inclusion and exclusion criteria for AC are not stated

				clearly.
Rajan 2013 [50]		X		Specific inclusion/ exclusion criteria are not stated, but they were the same for all patients in the AC group.
Rughani 2011 [51]		X		Same for all individuals
Sacko 2010 [52]		X		Same for all individuals
Sanus 2015 [53]		X		Same for all individuals.
See 2007 [54]			X	Predefined inclusion and exclusion criteria are not stated at all.
Serletis 2007 [55]	X			All patients with supratentorial lesion independent of the proximity to eloquent brain areas were prospectively included. It has to be expected that the patient selection and pre-operative treatment during 15 years long recruiting period has changed due to a learning curve.
Shinoura 2013 [57]			X	Inclusion and exclusion criteria are stated very vague. It is unclear what were the inclusion/ exclusion criteria for AC.
Sinha 2007 [58]		X		Same for all individuals, they were chosen to undergo AC according to clinical routine criteria. BIS groups were built retrospectively.
Sokhal 2015		X		Same for all individuals, they were chosen to undergo AC according to

[59]			clinical routine criteria.
Souter 2007 [60]		**X**	Predefined inclusion and exclusion criteria are not stated.
Wrede 2011 [61]	**X**		Same for all individuals.
Zhang 2008 [62]		**X**	Predefined inclusion and exclusion criteria are not stated.

***Selection bias, confounding* Q2: Does the strategy for recruiting participants into the study differ across groups/ respectively the individuals (if only one group) of the study?**

**Study**	**Yes, differs**	**No, does not differ**	**Cannot determine**	**Explanation**
Abdou 2010 [17]		**X**		Same for all individuals.
Ali 2009 [18]		**X**		Same for all individuals.
Amorim 2008 [19]			**X**	The selection of the 12 cases is not clearly described.
Andersen 2010 [20]		**X**		They analysed retrospectively the first 44 cases of their institution.
Beez 2013 [21]		**X**		Same for all patients meeting inclusion/exclusion criteria.

Bilotta 2014 [10]		X		Same for all individuals.
Boetto 2015 [22]		X		The recruitment into the two groups was made retrospectively according to the occurrence of intraoperative seizures.
Cai 2013 [23]			X	Predefined recruiting strategy is not stated.
Chacko 2013 [24]		X		All patients meeting inclusion/exclusion criteria were considered eligible. Patients were retrospectively analysed according to their histological diagnosis of glioma.
Chaki 2014 [25]			X	Predefined recruiting strategy is not stated.
Conte 2013 [26]		X		Consecutive inclusion of all patients undergoing AC in the pre-specified time span.
Deras 2012 [27]		X		Same for all individuals.
Garavaglia 2014 [28]			X	Predefined recruiting strategy of the included 10 patients is not clearly stated.
Gonen 2014 [29]		X		All primary AC surgeries conducted during the study period were included retrospectively.
Grossman 2007 [30]		X		Consecutive inclusion of the patients undergoing AC is reported.
Grossman 2013 [31]	X			Consecutively performed AC in patients with supratentorial lesion located within or in proximity to eloquent brain areas, but long time span that may include a learning curve of the AC team in regard to the patient selection and recruitment.
Hansen 2013 [33]		X		Same for all patients.

Hervey-Jumper 2015 [34]		**X**		Same for all patients.
Ilmberger 2008 [35]		**X**		Same for all patients.
Kim 2009 [37]		**X**		Same for all patients
Li 2015 [38]		**X**		Same for all patients meeting inclusion/exclusion criteria.
Lobo 2007 [39]			**X**	The selection of the 8 cases is not clearly described.
Low 2007 [40]		**X**		All patients with AC and MAC anaesthesia technique in the predefined time-span were included.
McNicholas 2014 [41]			**X**	Predefined recruiting strategy is not stated.
Nossek 2013 [42]	**X**			The patients were recruited consecutively to undergo AC in the predefined time span, but there were changes according to the learning curve of the AC team in selection of the patients.
Nossek 2013 [43]	**X**			The patients were recruited consecutively to undergo AC in the predefined time span, but there were changes according to the learning curve of the AC team in selection of the patients to undergo AC.
Olsen 2008 [44]		**X**		They analysed retrospectively the first 25 "asleep-awake" cases of their institution.
Ouyang 2013 [45]		**X**		All primary AC surgeries meeting the inclusion criterion of midline shift conducted during the study period were included retrospectively.
Ouyang 2013 [46]		**X**		All primary AC surgeries conducted during the study period were included retrospectively.

Pereira 2008 [47]		**X**		Same for all individuals. Patients were included into the two groups based on the recruitment period.
Peruzzi 2011 [48]		**X**		Same for all individuals.
Pinsker 2007 [49]		**X**		Same for all individuals.
Rajan 2013 [50]		**X**		Retrospective inclusion of all patients with supratentorial brain tumour resection between 2007-2010.
Rughani 2011 [51]		**X**		Same for all patients according to the performed kind of surgery.
Sacko 2010 [52]		**X**		Same for all patients according to the performed kind of surgery.
Sanus 2015 [53]		**X**		Same for all patients meeting inclusion/exclusion criteria.
See 2007 [54]		**X**		Same for all individuals, as they were consecutively included into the study.
Serletis 2007 [55]	**X**			The patients were recruited consecutively to undergo AC in the predefined time span, but there were changes according to the learning curve of the AC team in selection of the patients.
Shinoura 2013 [57]			**X**	It is unclear, f they had included all AC cases for tumors near motor region in the predefined time-span, or if they have selected the 102 patients from a bigger AC group during the time-span.
Sinha 2007 [58]		**X**		All primary AC surgeries conducted during the study period were included retrospectively. Furthermore, patients were retrospectively analysed according to the use of BIS monitoring in the

				clinical routine.
Sokhal 2015 [59]		X		Inclusion into the study was retrospectively done of all AC patients in the pre-defined time span.
Souter 2007 [60]		X		Same for all individuals, who were retrospectively included due to the use of dexmedetomidine for AC.
Wrede 2011 [61]		X		Same for all individuals.
Zhang 2008 [62]			X	Predefined recruiting strategy is not stated.

***Selection bias, confounding* Q3: Is the selection of the comparison group inappropriate, after taking into account feasibility and ethical considerations?**

**Study**	**Yes**	**No**	**Cannot determine or no description of the derivation of the comparison group**	**N/A: no comparison group (case series, one study group)**	**Explanation**
Abdou 2010 [17]				X	One study group, case series.
Ali 2009 [18]				X	One study group in regard to AC.
Amorim 2008 [19]				X	One study group, case series.

Andersen 2010 [20]		**X**	One study group, case series.
Beez 2013 [21]		**X**	One study group from different centres, but no comparison groups.
Bilotta 2014 [10]		**X**	One study group, case series.
Boetto 2015 [22]	**X**		Comparison groups were retrospectively built depending on the occurrence of intraoperative seizures.
Cai 2013 [23]		**X**	One study group, case series.
Chacko 2013 [24]		**X**	One study group.
Chaki 2014 [25]		**X**	One study group.
Conte 2013 [26]		**X**	One study group.
Deras 2012 [27]		**X**	One study group.
Garavaglia 2014 [28]		**X**	One study group, case series.
Gonen 2014 [29]	**X**		Comparison groups were retrospectively built depending on the tumour localisation.
Grossman 2007 [30]		**X**	One study group.

Grossman 2013 [31]	**X**		Comparison groups retrospectively chosen according to the age.
Hansen 2013 [33]		**X**	One study group.
Hervey-Jumper 2015 [34]		**X**	One study group.
Ilmberger 2008 [35]		**X**	One study group.
Kim 2009 [37]		**X**	One study group.
Li 2015 [38]		**X**	One study group.
Lobo 2007 [39]		**X**	One study group, case series.
Low 2007 [40]		**X**	One study group.
McNicholas 2014 [41]		**X**	One study group, case series.
Nossek 2013 [42]	**X**		Comparison group was retrospectively determined according to AC failure.
Nossek 2013 [43]	**X**		Comparison group was retrospectively determined according to intraoperative seizures.
Olsen 2008 [44]		**X**	One study group.
Ouyang 2013 [45]	**X**		Comparison group was retrospectively determined according to the amount

			of midline shift.
Ouyang 2013 [46]	**X**		Comparison group was retrospectively determined according to tumour dignity.
Pereira 2008 [47]		**X**	One study group.
Peruzzi 2011 [48]		**X**	Case series with two study groups in consecutive order.
Pinsker 2007 [49]		**X**	One study group.
Rajan 2013 [50]	**X**		Comparison group was retrospectively determined according to anaesthesia technique (awake vs. general anaesthesia)
Rughani 2011 [51]		**X**	One study group, case series.
Sacko 2010 [52]		**X**	One study group.
Sanus 2015 [53]		**X**	One study group, case series.
See 2007 [54]		**X**	One study group.
Serletis 2007 [55]	X		Comparison group was built depending on the tumour location.
Shinoura 2013 [57]		**X**	One study group.

Sinha 2007 [58]		X				Comparison groups were retrospectively built depending on the use of BIS monitoring in the clinical routine.
Sokhal 2015 [59]		**X**				Comparison groups were retrospectively built depending on the mean anaesthetic drug.
Souter 2007 [60]					**X**	One study group, case series.
Wrede 2011 [61]					**X**	One study group in regard to AC.
Zhang 2008 [62]		**X**				Comparison groups retrospectively chosen according to the success of brain mapping.

***Performance bias* Q4: Does the study fail to account for important variations in the execution of the study from the proposed protocol**?

**Study**	**Yes**	**No**	**Partially**	**Cannot determine**	**N.A.: not an intervention study or no variations**	**Explanation**
Abdou 2010 [17]					**X**	No variations.
Ali 2009 [18]				**X**		The dosage of the applied anaesthetics is not reported in detail and may have varied across the individuals.

Amorim 2008 [19]		**X**			Study accounts for some outcomes according to the used anaesthesia regime.
Andersen 2010 [20]	**X**				Different application of drugs (e.g. midazolam, mannitol) across the study participants.
Beez 2013 [21]		**X**			Awake phase was pre-defined independent of the different centre protocols
Bilotta 2014 [10]			**X**		The dosage of the applied anaesthetics is not reported in detail and may have varied across the individuals.
Boetto 2015 [22]			**X**		The dosage of the applied anaesthetics is not reported in detail and may have varied across the individuals and the groups.
Cai 2013 [23]				**X**	No variations.
Chacko 2013 [24]				**X**	No variations.
Chaki 2014 [25]				**X**	No variations.
Conte 2013 [26]				**X**	No variations.
Deras 2012 [27]				**X**	No variations.
Garavaglia 2014 [28]				**X**	No variations.
Gonen 2014 [29]			**X**		The dosage of the applied anaesthetics is not reported in detail and may have varied across the study groups.

Grossman 2007 [30]	**X**				There were different anaesthetic dosages applied across the patients, but this was not taken into account for the data analysis.
Grossman 2013 [31]	**X**				Preoperative treatment in regard of anti-epileptic drugs changed during the entire study period. Furthermore, the dosage of the applied anaesthetics is not reported in detail and may have varied across the study groups.
Hansen 2013 [33]				**X**	No variations, except of the learning curve for regional scalp nerve block performance.
Hervey-Jumper 2015 [34]		**X**			Statistical analysis was executed to account for the 3 different anaesthesia regimen, which were used.
Ilmberger 2008 [35]			**X**		The dosage of the applied anaesthetics is not reported in detail and may have varied across the individuals.
Kim 2009 [37]				**X**	No variations.
Li 2015 [38]			**X**		The dosage of the applied anaesthetics is not reported at all and may have varied across the patients.
Lobo 2007 [39]				**X**	No variations.
Low 2007 [40]			**X**		The dosage of the applied anaesthetics is not reported in detail and may have varied across the individuals.
McNicholas 2014 [41]		**X**			Different execution of the regional scalp nerve block in the two study centres is

				discussed in the article.
Nossek 2013 [42]	**X**			Dosage and timing of preoperative anti-epileptic drugs differed between the patients. The dosage of the applied anaesthetics is not reported in detail and may have varied across the study groups.
Nossek 2013 [43]	**X**			Dosage and timing of preoperative anti-epileptic drugs differed between the patients. The dosage of the applied anaesthetics is not reported in detail and may have varied across the study groups.
Olsen 2008 [44]			**X**	No significant variations.
Ouyang 2013 [45]		**X**		Statistical analysis was performed to account for the surgery duration and the different amounts of used opioids and other drugs, which could lead to the result of the study.
Ouyang 2013 [46]		**X**		Statistical analysis was performed to account for surgery duration and the different amounts of used opioids and other drugs, which could lead to the result of the study.
Pereira 2008 [47]		**X**		Statistical analysis was performed to account for the different treatment modalities.
Peruzzi 2011 [48]			**X**	No variations. Retrospective analysis of the routine data. Only data of patients operated by one surgeon were used.
Pinsker 2007 [49]	**X**			Dosage anti-epileptic drugs and the applied anaesthetics is not reported in

						detail and may have varied across the study participants.
Rajan 2013 [50]		**X**				Statistical analysis was performed to account for the different amounts of used opioids and other drugs, which could lead to the result of the study.
Rughani 2011 [51]				**X**		The dosage of the applied anaesthetics is not reported in detail and may have varied across the individuals, as the anaesthetic protocol allowed different dosages.
Sacko 2010 [52]					**X**	No variations.
Sanus 2015 [53]					**X**	No variations.
See 2007 [54]			**X**			There is a statement about the used anaesthetic drugs, but it cannot be differentiated for each patient, which combination was used.
Serletis 2007 [55]	**X**					The dosage of the applied anaesthetics is not reported in detail and may have varied across the study groups. Furthermore, the study duration of 15 years, bears the risk of changes in the surgery/ anaesthesia technique accross the participants.
Shinoura 2013 [57]	**X**					The dosage of the applied anaesthetics is not reported in detail and may have varied across the study groups.
Sinha 2007 [58]					**X**	Variations e.g. in the anaesthetic dosage were analysed for both groups.
Sokhal 2015		**X**				Statistical analysis was performed to account for the two different anaesthetic

[59]				regimes.
Souter 2007 [60]	X			Anaesthesia regime is reported for each patient and taken into account for the results.
Wrede 2011 [61]		X		The dosage of the applied anaesthetics is not reported in detail and may have varied across the individuals, but it is stated that the same standardised protocol was used.
Zhang 2008 [62]		X		The dosage of the applied anaesthetics is not reported in detail and may have varied across the individuals.

***Detection bias* Q5: Was the assessor not blinded to the outcome, exposure, or intervention status of the participants?**

**Study**	**Yes, not blinded**	**No, blinded**	**Not applicable: assessor cannot be blinded**	**Explanation**
All studies	X			The postoperative outcome assessor could have been blinded in all studies to the kind of anaesthesia technique used. The intervention (AC) could not be blinded to the intraoperative assessors or the patients, who provided self-reports.

***Detection bias, confounding* Q6: Were valid and reliable measures, implemented consistently across all study participants used to assess inclusion/exclusion criteria, intervention/exposure outcomes, participant health benefits and harms, and confounding?**

	**Yes, valid and reliable measure**	**No, valid and reliable measure not**	**Cannot determine or measurement approach not**	**Explanation**

	used	used	reported	
Abdou 2010 [17]	**X**			The inclusion/ exclusion criteria were applied according to the clinical examination, which has to be assumed as valid and reliable form, intraoperative measures of vital data were measured with valid monitoring, sedation depth was measured with OAA/S which is a nearly valid and reliable measurement.
Ali 2009 [18]	**X**			Same for all individuals. Valid and reliable: pre- and postoperative CT and MRI, intraoperative vital parameter measurement, intraoperative neurological testing, intra-/postoperative assessment of pain, nausea and discomfort, postoperative neurological testing according to the clinical routine, and intraoperative occurrence of seizures.
Amorim 2008 [19]			**X**	Same for all individuals. Valid and reliable: pre- and postoperative CT and MRI and neurophysiological preoperative evaluation, but high risk for confounding in regard to the inclusion/exclusion criteria for AC.
Andersen 2010 [20]			**X**	High risk for confounding in regard to the inclusion/exclusion criteria for AC. Measures of outcomes are not described in detail.
Beez 2013 [21]	**X**			VAS pain and anxiety is a valid measurement. Patients' perception was a subjective measurement, but there is no other way to measure this outcome.
Bilotta 2014 [10]	**X**			Valid and reliable: Pre- and postoperative MRI, Preoperative clinical status and disability were evaluated with modified Rankin Score (mRS), perioperative language testing.
Boetto 2015 [22]	**X**			Inclusion and exclusion criteria for AC are not properly described, but the outcome assessment methods were implemented consistently across all study participants. Valid and reliable were: clinical measure of intraoperative seizures, death, haematoma, AC

		failure, postoperative MRI and postoperative clinical neurologic measure.
Cai 2013 [23]	**X**	Measures of ventilation and blood gas parameters and VAS pain scores are valid and reliable. Subjective: Observers Assessment of Alertness/Sedation Scale (OAA/S) was performed.
Chacko 2013 [24]	**X**	Inclusion criteria: MRI before surgery and age >13, histological diagnosis are valid and reliable measures. Furthermore language assessment, electrophysiological monitoring and assessment of intraoperative complications like seizures and hypoxia, which are described, are valid and reliable. The assessment of patient outcomes after surgery is not described in detail, but neurological testing in the clinical postoperative routine should be seen as a valid and reliable measurement.
Chaki 2014 [25]	**X**	Measures of blood pressure, heart rate, blood concentrations are valid and reliable.
Conte 2013 [26]	**X**	BIS values and time to remove laryngeal mask are valid and reliable measurements.
Deras 2012 [27]	**X**	All measures were implemented consistently across all patients. Inclusion measures, emergence times, and adverse events detection were objective and reliable (e.g. weight, mouth opening). The other outcomes were subjective, but same across all patients.
Garavaglia 2014 [28]	**X**	Blood gas analyses were performed, to ensure safe anaesthesia. These are valid and reliable measurements. Other outcomes were assessed by subjective observations, but consistently across all study participants.
Gonen 2014 [29]	**X**	Valid and reliable: Immunohistochemistry measurement of IDH1 mutation, preoperative and postoperative MRI scans. Clinical identification of intraoperative seizures can be

			assumed to be a valid measurement as it is a common procedure in clinical routine.
Grossman 2007 [30]		**X**	Inclusion and exclusion criteria are not properly described. Pain detection is always depending on subjective measures, therefore this can be attributed to a valid measurement, but reliability remains questionable.
Grossman 2013 [31]	**X**		Valid and reliable: pre- and postoperative MRI scans. The assessment of patient outcomes after surgery is not described in detail, but neurological testing and assessment of postoperative complications in the clinical postoperative routine should be seen as valid and reliable measurements. Clinical identification of intraoperative seizures can be assumed to be a valid measurement as it is a common procedure in clinical routine.
Hansen 2013 [33]	**X**		Same for all individuals. Valid and reliable: pre- and postoperative MRI, intraoperative vital parameter measurement, intraoperative neuropsychological testing, intra-/postoperative assessment of VAS pain and discomfort.
Hervey-Jumper 2015 [34]	**X**		Same for all individuals. Valid and reliable: preoperative MRI and neurophysiological testing, electrocorticography and intraoperative testing.
Ilmberger 2008 [35]	**X**		Same for all individuals. Valid and reliable: preoperative MRI and neurological examination, electrocorticography and intraoperative testing and postoperative MRI / CT scan.
Kim 2009 [37]	**X**		Same for all individuals. Valid and reliable: preoperative MRI and neurophysiological testing, electrocorticography and intraoperative testing and postoperative MRI scan.

Li 2015 [38]	X			Valid and reliable: Pre- and postoperative MRI, pre-operative MMSE, postoperative neurological testing. Of note, there was an additional functional MRI testing in patients after 2006, but this did not serve for the inclusion criterion.
Lobo 2007 [39]	X			Same for all individuals. Valid and reliable: preoperative MRI, intraoperative neuropsychological testing, and clinical assessment of intraoperative seizures.
Low 2007 [40]	X			Inclusion and Exclusion criteria for AC are not properly described. Other outcome measures have to be seen valid and reliable. Especially the postoperative MRI and the evaluation of the extent of resection is a valid technique.
McNicholas 2014 [41]	X			Clinical detection of postoperative facial nerve palsy was predefined, and has to be seen as a valid measure.
Nossek 2013 [42]	X			Recruiting was subjective, but comparison groups were retrospectively built, therefore the risk for selection bias for the comparison groups can be seen negligibly. Valid and reliable: pre- and postoperative MRI scans. Clinical identification of intraoperative seizures can be assumed to be a valid measurement as it is a common procedure. The assessment of patient outcomes after surgery is not described in detail, but neurological testing and assessment of postoperative complications in the clinical postoperative routine should be seen as valid and reliable measurements.
Nossek 2013 [43]		X		Recruiting was subjective, but comparison groups were retrospectively built, therefore the risk for selection bias for the comparison groups can be seen negligibly. Valid and reliable: Preoperative functional MRI, use of electrocorticography. But this was only inconsistently performed in some patients. Therefore the decision: No

Olsen 2008 [44]	**X**	Recruiting into the AC surgery was subjective, but the clinical assessment of intraoperative seizures and complications are usual procedures and all consecutive first 25 AC patients were included in the study.
Ouyang 2013 [45]	**X**	Valid and reliable VAS pain, 4 point scale for nausea, drug doses, demographic and perioperative data.
Ouyang 2013 [46]	**X**	Inclusion/ exclusion criteria measures not stated. Valid and reliable VAS pain, 4 point scale for nausea, drug doses, demographic and perioperative data.
Pereira 2008 [47]	**X**	Valid and reliable: preoperative and postoperative MRI and neurological testing according to the clinical routine. Clinical identification of intraoperative seizures, respiratory complications and neurological dysfunction can be assumed to be valid measurements as they are common procedures.
Peruzzi 2011 [48]	**X**	Inclusion/exclusion criteria were clearly stated and supported by valid and reliable radiological and pathological evidence. Outcomes were also valid and reliable assessed.
Pinsker 2007 [49]	**X**	Recruiting into the AC surgery was subjective, but the clinical assessment of intraoperative seizures and complications are usual procedures. The extent of tumour resection was determined with postoperative MRI, which is a valid and reliable technique.
Rajan 2013 [50]	**X**	Valid and reliable: demographic data, haemodynamic and respiratory measurements, VAS pain, tumour histology and location, drug doses. Clinical identification of intraoperative seizures can be assumed to be a valid measurement as it is a common procedure.

Rughani 2011 [51]	**X**			Valid and reliable: preoperative MRI and neurological testing according to the clinical routine. Clinical identification of intraoperative seizures, respiratory complications and neurological dysfunction can be assumed to be valid measurements as they are common procedures.
Sacko 2010 [52]	**X**			Valid and reliable: preoperative and postoperative imaging to localise the brain tumours/ resection rate. Intraoperative measurement of haemodynamic and respiratory data. Clinical identification of intraoperative seizures and postoperative neurological outcomes can be assumed to be valid measurements as they are common procedures.
Sanus 2015 [53]	**X**			Valid and reliable: preoperative and postoperative imaging to localise the brain tumours/ resection rate. Intraoperative measurement of haemodynamic and respiratory data. Clinical identification of intraoperative seizures and postoperative neurological outcomes can be assumed to be valid measurements as they are common procedures.
See 2007 [54]			**X**	Inclusion/ exclusion criteria for patient selection are not stated. Intraoperative outcome variables were assessed valid and reliable in the clinical routine, by the routine anaesthesia monitoring. Neurological outcome assessment is not described in detail.
Serletis 2007 [55]		**X**		Recruiting was subjective, but comparison groups were built according to the tumour location, therefore the risk for selection bias for the comparison groups can be seen negligibly. Valid and reliable: Preoperative functional MRI, but this was only inconsistently performed in some patients. Therefore the decision: No

Shinoura 2013 [57]		**X**	High risk for confounding in regard to the inclusion/exclusion criteria for AC. Preoperative fMRI and DTI with continuous monitoring of motor function are valid and reliable. Clinical neurological examination has to be assumed to be valid and reliable.
Sinha 2007 [58]	**X**		Valid and reliable: preoperative imaging to localise the brain foci, clinical identification of intraoperative seizures and postoperative neurological outcomes can be assumed to be valid measurements as they are common procedures.
Sokhal 2015 [59]	**X**		Valid and reliable: intraoperative measurement of haemodynamic and respiratory data. The assessment of patient outcomes after surgery is not described in detail, but neurological testing and assessment of postoperative complications in the clinical postoperative routine should be seen as valid and reliable measurements. Clinical identification of intraoperative seizures can be assumed to be a valid measurement as it is a common procedure in clinical routine.
Souter 2007 [60]		**X**	High risk for confounding in regard to the inclusion/exclusion criteria for AC, clinical measure of sedation depth (Observer Assessment of Alertness/Sedation (OAA/S) score) is a subjective measure. Detection of seizures with the use of ECoG is a valid and reliable measure.
Wrede 2011 [61]	**X**		An interdisciplinary board, with the aid of a functional preoperative MRI, decided which patient should undergo AC. This should be seen as a valid and reliable inclusion approach. Postoperative CCT and MRI and clinical neurological testing are valid

				measures in the clinical routine.
Zhang 2008 [62]			X	Valid and reliable: pre- and postoperative MRI and neurological testing, but high risk for confounding in regard to the inclusion/exclusion criteria for AC.

Attrition bias **Q7: Was the length of follow-up different across study groups?**

**Study**	**Yes, different or cannot determine**	**No, not different or remedied through analysis**	**N.A.: cross-sectional or only one group followed over time**	**Explanation**
Abdou 2010 [17]		X		Length of follow up was the same for all patients.
Ali 2009 [18]		X		Length of follow up was the same for all patients.
Amorim 2008 [19]		X		Length of follow up was the same for all patients.
Andersen 2010 [20]		X		Length of follow up was the same for all patients.
Beez 2013 [21]		X		Length of follow up was the same for all patients.
Bilotta 2014 [10]		X		Length of follow up was the same for all patients.
Boetto 2015 [22]		X		Length of follow up was the same for all patients.
Cai 2013 [23]		X		Length of follow up was the same for all patients.
Chacko 2013 [24]	X			Length of follow up is not described in detail. Only the mean

			follow up time is mentioned with 40.8 months.
Chaki 2014 [25]		**X**	Length of follow up was the same for all patients.
Conte 2013 [26]		**X**	All patients were only followed up until the end of the surgery.
Deras 2012 [27]		**X**	Length of follow up was the same for all patients
Garavaglia 2014 [28]		**X**	Length of follow up was the same for all patients
Gonen 2014 [29]	**X**		12 patients were lost to follow up for outcome assessment and 25 for isocitrate dehydrogenase 1 analysis and no statistical adjustment was performed.
Grossman 2007 [30]		**X**	Length of follow up was the same for all patients.
Grossman 2013 [31]		**X**	Length of follow up was the same for all patients
Hansen 2013 [33]		**X**	Length of follow up was the same for all patients
Hervey-Jumper 2015 [34]		**X**	Length of follow up was the same for all patients
Ilmberger 2008 [35]		**X**	Length of follow up was the same for all patients
Kim 2009 [37]		**X**	Length of follow up was the same for all patients.

Li 2015 [38]		**X**	Length of follow up was the same for all patients
Lobo 2007 [39]		**X**	Length of follow up was the same for all patients.
Low 2007 [40]		**X**	Length of follow up was the same for all patients.
McNicholas 2014 [41]		**X**	Length of follow up was the same for all patients
Nossek 2013 [42]		**X**	Length of follow up was the same for all patients
Nossek 2013 [43]		**X**	Length of follow up was the same for all patients
Olsen 2008 [44]		**X**	Length of follow up was the same for all patients
Ouyang 2013 [45]		**X**	Length of follow up was the same for all retrospectively included patients.
Ouyang 2013 [46]		**X**	Length of follow up was the same for all retrospectively included patients.
Pereira 2008 [47]	**X**		All patients were followed up until 2008, independently of the AC time-point, which has differed between the two groups and within the patients of one group.
Peruzzi 2011 [48]		**X**	Length of follow up was the same for all retrospectively included patients.

Pinsker 2007 [49]		X	Length of follow up was the same for all retrospectively included patients.
Rajan 2013 [50]		X	Length of follow up was the same for all retrospectively included patients.
Rughani 2011 [51]		X	Length of follow up was the same for all retrospectively included patients.
Sacko 2010 [52]	X		Follow up period varied across the study participants from 21-111 months postoperatively.
Sanus 2015 [53]	X		Follow up period varied across the study participants from 15 days - 55 months postoperatively.
See 2007 [54]		X	Length of follow up was the same for all patients.
Serletis 2007 [55]		X	Length of follow up was the same for all patients.
Shinoura 2013 [57]		X	Length of follow up was the same for all patients.
Sinha 2007 [58]		X	Length of follow up was the same for all patients.
Sokhal 2015 [59]	X		Length of follow up is not described in detail, may have varied.
Souter 2007		X	Length of follow up was the same for all patients.

[60]		
Wrede 2011 [61]	**X**	Length of follow up was the same for all patients.
Zhang 2008 [62]	**X**	Length of follow up was the same for all patients

Attrition bias, detection bias **Q8: In case of high loss to follow up (or differential loss to follow up, was the impact assessed (e.g., through sensitivity analysis or other adjustment method)?**

**Study**	**Yes, impact assessed**	**No, impact not assessed**	**Cannot determine**	**N.A.: no loss to follow-up or loss to follow-up was not considered to be high, cross- sectional study, or case-control study selected on outcome**	**Explanation**
Abdou 2010 [17]				**X**	No loss.
Ali 2009 [18]				**X**	Only 5% loss to follow up.
Amorim 2008 [19]				**X**	No loss.
Andersen 2010 [20]				**X**	No loss.
Beez 2013 [21]				**X**	Less than 20% attrition.
Bilotta 2014 [10]				**X**	No loss.

Boetto 2015 [22]		X	No loss.
Cai 2013 [23]		X	No loss.
Chacko 2013 [24]	X		Not mentioned.
Chaki 2014 [25]		X	No loss.
Conte 2013 [26]		X	Loss to follow up only for 10% of 30 patients
Deras 2012 [27]		X	No loss.
Garavaglia 2014 [28]		X	No loss.
Gonen 2014 [29]		X	Less than 20% attrition.
Grossman 2007 [30]		X	No loss.
Grossman 2013 [31]		X	No loss.
Hansen 2013 [33]		X	No loss.
Hervey-Jumper 2015 [34]		X	No loss.
Ilmberger 2008 [35]		X	Less than 20% attrition.

Kim 2009 [37]	**X**	No loss.
Li 2015 [38]	**X**	No loss.
Lobo 2007 [39]	**X**	No loss.
Low 2007 [40]	**X**	No loss.
McNicholas 2014 [41]	**X**	No loss.
Nossek 2013 [42]	**X**	No loss.
Nossek 2013 [43]	**X**	Loss to follow up only 1% (5 patients).
Olsen 2008 [44]	**X**	No loss.
Ouyang 2013 [45]	**X**	No loss.
Ouyang 2013 [46]	**X**	No loss.
Pereira 2008 [47]	**X**	No loss reported, but the outcomes at the end of follow up in 2008 are only insufficiently reported.
Peruzzi 2011 [48]	**X**	No loss.
Pinsker 2007 [49]	**X**	No loss.

Rajan 2013 [50]			X	No loss.
Rughani 2011 [51]			X	No loss.
Sacko 2010 [52]	X			High difference in follows up and impact not assessed.
Sanus 2015 [53]	X			High difference in follows up and impact not assessed.
See 2007 [54]			X	No loss.
Serletis 2007 [55]			X	No loss.
Shinoura 2013 [57]			X	No loss.
Sinha 2007 [58]			X	No loss.
Sokhal 2015 [59]		X		Follow up time and losses are not reported.
Souter 2007 [60]			X	No loss.
Wrede 2011 [61]			X	No loss.
Zhang 2008 [62]			X	No loss.

Selective outcome reporting **Q9: Are any important primary outcomes missing from the results?**				
**Study**	**Yes, important outcome(s) missing**	**No important outcome(s) missing**	**Cannot determine**	**Explanation**
Abdou 2010 [17]		X		All reported.
Ali 2009 [18]		X		All reported.
Amorim 2008 [19]		X		All reported.
Andersen 2010 [20]			X	Outcomes are not predefined in the publication.
Beez 2013 [21]		X		All reported.
Bilotta 2014 [10]		X		All reported.
Boetto 2015 [22]		X		All reported.
Cai 2013 [23]		X		All reported.
Chacko 2013 [24]			X	It remains possible that the length of follow up differed between the patients, because not thoroughly reported.
Chaki 2014 [25]		X		All reported.

Conte 2013 [26]	**X**	All reported.
Deras 2012 [27]	**X**	All reported.
Garavaglia 2014 [28]	**X**	All reported.
Gonen 2014 [29]	**X**	All reported.
Grossman 2007 [30]	**X**	All reported.
Grossman 2013 [31]	**X**	All reported.
Hansen 2013 [33]	**X**	All reported.
Hervey-Jumper 2015 [34]	**X**	All reported.
Ilmberger 2008 [35]	**X**	All reported.
Kim 2009 [37]	**X**	All reported.
Li 2015 [38]	**X**	All reported.
Lobo 2007 [39]	**X**	All reported. It would have been favourable that the study had reported some neurological outcomes, but that was not the aim of the study.
Low 2007 [40]	**X**	All reported.

McNicholas 2014 [41]	**X**	All reported.
Nossek 2013 [42]	**X**	All reported.
Nossek 2013 [43]	**X**	All reported.
Olsen 2008 [44]	**X**	All reported.
Ouyang 2013 [45]	**X**	It would have been favourable that the study had reported some neurological outcomes. But the aim of this study was solely to answer the question for PONV and postoperative pain after AC in specific patients.
Ouyang 2013 [46]	**X**	It would have been favourable that the study had reported some neurological outcomes. But the aim of this study was solely to answer the question for PONV and postoperative pain after AC in specific patients.
Pereira 2008 [47]	**X**	All reported.
Peruzzi 2011 [48]	**X**	All reported.
Pinsker 2007 [49]	**X**	All reported.
Rajan 2013 [50]	**X**	It would have been favourable that the study had reported some neurological outcomes. But the aim of this study was solely to answer the question if AC with propofol and dexmedetomidine is a safe procedure compared to general anaesthesia.

Rughani 2011 [51]		**X**		All reported.
Sacko 2010 [52]		**X**		All reported.
Sanus 2015 [53]		**X**		All reported.
See 2007 [54]		**X**		All reported.
Serletis 2007 [55]		**X**		All reported.
Shinoura 2013 [57]		**X**		All reported.
Sinha 2007 [58]		**X**		All reported.
Sokhal 2015 [59]		**X**		All reported.
Souter 2007 [60]		**X**		All reported.
Wrede 2011 [61]		**X**		All reported.
Zhang 2008 [62]				
**Study**	**Yes, important outcome(s)**	**No important outcome(s) missing**	**Assessment of harms N.A. to**	**Explanation**

missing	this study	
Abdou 2010 [17]	**X**	All reported.
Ali 2009 [18]	**X**	All reported.
Amorim 2008 [19]	**X**	All reported.
Andersen 2010 [20]	**X**	The most common intraoperative complications were assessed.
Beez 2013 [21]	**X**	The aim of the study regarding harms was only to describe intraoperative seizures and this is presented in the article.
Bilotta 2014 [10]	**X**	The aim of the study regarding harms was to describe intraoperative language disability and impairment at early and long-term follow-up and this is presented in the article.
Boetto 2015 [22]	**X**	All reported.
Cai 2013 [23]	**X**	All reported.
Chacko 2013 [24]	**X**	All reported.
Chaki 2014 [25]	**X**	All applicable for this study were reported.
Conte 2013 [26]	**X**	All reported.

Deras 2012 [27]	**X**	All reported.
Garavaglia 2014 [28]	**X**	The aim of the study regarding harms was to describe intraoperative seizures and this is presented in the article.
Gonen 2014 [29]	**X**	All harms related to the primary endpoint of this study are reported.
Grossman 2007 [30]	**X**	All reported.
Grossman 2013 [31]	**X**	All reported.
Hansen 2013 [33]	**X**	All reported.
Hervey-Jumper 2015 [34]	**X**	All reported.
Ilmberger 2008 [35]	**X**	All reported.
Kim 2009 [37]	**X**	All reported.
Li 2015 [38]	**X**	All reported.
Lobo 2007 [39]	**X**	All reported.
Low 2007 [40]	**X**	The aim of the study regarding harms was to describe postoperative facial nerve palsy and this is presented in the article.

McNicholas 2014 [41]	**X**	All reported.
Nossek 2013 [42]	**X**	All reported.
Nossek 2013 [43]	**X**	All reported.
Olsen 2008 [44]	**X**	All reported.
Ouyang 2013 [45]	**X**	All reported.
Ouyang 2013 [46]	**X**	All reported.
Pereira 2008 [47]	**X**	All reported.
Peruzzi 2011 [48]	**X**	All reported.
Pinsker 2007 [49]	**X**	All reported.
Rajan 2013 [50]	**X**	All reported.
Rughani 2011 [51]	**X**	All reported.
Sacko 2010 [52]	**X**	All reported.
Sanus 2015 [53]	**X**	All reported.

See 2007 [54]	X	All reported, but unfortunately not consequently divided into the two groups, for all outcomes.
Serletis 2007 [55]	X	All reported.
Shinoura 2013 [57]	X	All reported.
Sinha 2007 [58]	X	All reported.
Sokhal 2015 [59]	X	All in regard to the study endpoints are reported, but unfortunately neurological outcomes were not reported.
Souter 2007 [60]	X	All in regard to the study endpoints are reported, but unfortunately intraoperative complications were not reported.
Wrede 2011 [61]	X	All reported.
Zhang 2008 [62]		

**Study**	**Yes, believable**	**No, not believable**	**Explanation**
Abdou 2010 [17]		X	Study limitations are not mentioned.
Ali 2009 [18]		X	Study limitations are not mentioned.

Amorim 2008 [19]		**X**	Study limitations are not mentioned.
Andersen 2010 [20]		**X**	Study limitations are not mentioned.
Beez 2013 [21]	**X**		They consider, that every neurosurgical centre used their own AC protocol. They take into account the missing follow up data for some patients.
Bilotta 2014 [10]		**X**	Study limitations are not mentioned. To small sample size, to generalise their results.
Boetto 2015 [22]	**X**		They consider the limitation of generalisability due to the performance of AC by the same surgeon. Furthermore, that all patients with seizure history continued their anticonvulsant medication on the surgery day.
Cai 2013 [23]		**X**	Study limitations are not mentioned.
Chacko 2013 [24]	**X**		Study limitations are discussed; for example the lack of a control group
Chaki 2014 [25]		**X**	Study limitations are not mentioned at all.
Conte 2013 [26]	**X**		Study limitations are discussed; for example the lack of evidence in this study for the usefulness of BIS monitoring in awake craniotomy with dexmedetomidine. Furthermore, the decision to perform brain mapping was not depending on the BIS values, but routinely made by the neuropsychologist.
Deras 2012 [27]		**X**	Study limitations are not mentioned.
Garavaglia 2014 [28]	**X**		They report that this was only a case study and that they will implement the anaesthetic approach in their clinical routine. This approach has to be compared to remifentanil-propofol in high-risk patients in future, to further define its safety.

Gonen 2014 [29]	**X**		The general limitation of a retrospective study is discussed (inherent limitations and potential bias such as selection bias, loss to follow-up, and observation bias). The most patients (82%) had left hemisphere tumours, although seizures are more common in right hemisphere tumours. Loss to follow up is explained.
Grossman 2007 [30]	**X**		The limitation of the lack of a control group and of a short (12 hours) follow up are reported.
Grossman 2013 [31]	**X**		The general limitation of a retrospective study is mentioned. Decision to select patients for awake craniotomy was the same for all patients, but they were all in a relatively good state of health and able to cooperate.
Hansen 2013 [33]		**X**	No study limitations mentioned.
Hervey-Jumper 2015 [34]	**X**		The general limitation of a retrospective study is mentioned. Furthermore the methodology in this study has evolved over time.
Ilmberger 2008 [35]		**X**	Study limitations are not mentioned.
Kim 2009 [37]		**X**	Study limitations are not mentioned.
Li 2015 [38]	**X**		They mention that a larger prospective study may be needed to evaluate the differences between drugs and the preferred location of the injection site.
Lobo 2007 [39]		**X**	Study limitations are not mentioned.

Low 2007 [40]		X	Study limitations are not mentioned.
McNicholas 2014 [41]	X		It is mentioned that their results are restricted to the Chinese population.
Nossek 2013 [42]	X		The general limitation of a retrospective study is mentioned. The learning curve effect of all involved persons during the study period (including recruitment decisions, anaesthesia procedure and surgery) id mentioned.
Nossek 2013 [43]	X		The general limitation of a retrospective study and the experience gained over the years are mentioned.
Olsen 2008 [44]		X	No, only the small number of the included patients is mentioned.
Ouyang 2013 [45]	X		The retrospective analysis of nausea and pain scores has to be seen within its limits. Furthermore the scores were assessed by different individuals and at possibly different time-points.
Ouyang 2013 [46]	X		The data have been retrospectively collected. Nausea and pain scores were not recorded by the same individual or at fully standardized time intervals.
Pereira 2008 [47]	X		They mention the limitation of their uncontrolled study and that the groups were not matched. Therefore the results for each group have to be interpreted with caution.
Peruzzi 2011 [48]	X		The general limitation of a retrospective study and small number of patients is discussed satisfactorily.
Pinsker 2007 [49]		X	Study limitations are not mentioned.
Rajan 2013 [50]	X		The data have been retrospectively collected. Only patients with complete records were included in this study.
Rughani 2011	X		They discuss their conservative resection due to their first experience with awake craniotomy and the lack of

[51]			neuropsychologists or speech therapists in their procedure.
Sacko 2010 [52]		X	Study limitations are not mentioned.
Sanus 2015 [53]	X		The small study size is considered as a limitation factor for the results.
See 2007 [54]		X	Study limitations are not mentioned.
Serletis 2007 [55]		X	The main limitation of the very long study duration, with a significant learning curve, was not considered.
Shinoura 2013 [57]		X	Study limitations are not mentioned.
Sinha 2007 [58]	X		Study limitations are discussed; for example the learning curve of the surgeons and the used agents like haloperidol, with its possible adverse effects.
Sokhal 2015 [59]	X		BIS values may not correlate correctly with patient consciousness, in patients on anti-epileptic medication. Steep learning curve of the surgeons.
Souter 2007 [60]		X	Study limitations are not mentioned.
Wrede 2011 [61]	X		They discuss the generalisability of the used questionnaires to all neurosurgical patients, as they could not be validated in this study. Furthermore, it was a German questionnaire. The study group was heterogeneous and small. The assignment of the patients to the AC or GA technique was probably biased, but objectiveness was achieved by a interdisciplinary decision board.

Zhang 2008 [62]	**X**	Study limitations are not mentioned.

***Confounding* Q12: Any attempt to balance the allocation between the groups or match groups (e.g., through stratification, matching, propensity scores).**

**Study**	**Yes or study accounts for imbalance between groups through a post hoc approach such as multivariate analysis**	**No or cannot determine**	**Not applicable: study does not include a comparison group (case series or one study group)**	**Explanation**
Abdou 2010 [17]			**X**	One study group.
Ali 2009 [18]			**X**	One study group of AC.
Amorim 2008 [19]			**X**	One study group.
Andersen 2010 [20]			**X**	One study group.
Beez 2013 [21]			**X**	One study group.
Bilotta 2014 [10]			**X**	One study group.
Boetto 2015 [22]	**X**			Between group differences were assessed by statistical methods.
Cai 2013 [23]			**X**	One study group.

Chacko 2013 [24]			**X**	One study group.
Chaki 2014 [25]			**X**	One study group.
Conte 2013 [26]			**X**	One study group.
Deras 2012 [27]			**X**	One study group.
Garavaglia 2014 [28]			**X**	One study group.
Gonen 2014 [29]		**X (No)**		The sizes of the study groups differed. No attempt was made to match the groups.
Grossman 2007 [30]			**X**	One study group.
Grossman 2013 [31]	**X (Yes)**			Multivariate Cox regression analysis performed.
Hansen 2013 [33]			**X**	One study group.
Hervey-Jumper 2015 [34]			**X**	One study group.
Ilmberger 2008 [35]			**X**	One study group.
Kim 2009 [37]			**X**	One study group.
Li 2015 [38]			**X**	One study group.

Lobo 2007 [39]		**X**	One study group.
Low 2007 [40]		**X**	One study group.
McNicholas 2014 [41]		**X**	One study group.
Nossek 2013 [42]	**X (No)**		The sizes of the study groups differed. No attempt was made to match the groups.
Nossek 2013 [43]	**X (No)**		The sizes of the study groups differed. No attempt was made to match the groups.
Olsen 2008 [44]		**X**	One study group.
Ouyang 2013 [45]	**X (No)**		The sizes of the study groups differed. No attempt was made to match the groups.
Ouyang 2013 [46]	**X (No)**		The sizes of the study groups differed. No attempt was made to match the groups.
Pereira 2008 [47]	**X (No)**		No attempt was made to match the groups.
Peruzzi 2011 [48]		**X**	One awake craniotomy study group.
Pinsker 2007 [49]		**X**	One awake craniotomy study group.
Rajan 2013 [50]		**X**	Only one AC group.

Rughani 2011 [51]		**X**	One study group.
Sacko 2010 [52]		**X**	Only one AC group.
Sanus 2015 [53]		**X**	One study group.
See 2007 [54]		**X**	One study group.
Serletis 2007 [55]	**X (Cannot determine)**		The study groups were built, depending on the tumour location. The aim of the study was to analyse all patients undergoing AC, irrespective of the tumour location.
Shinoura 2013 [57]		**X**	One study group.
Sinha 2007 [58]	**X (No)**		The sizes of the study groups differed. No attempt was made to match the groups.
Sokhal 2015 [59]		**X**	The sizes of the study groups differed. No attempt was made to match the groups. But it was not the aim of the study, to explore differences between the groups. It was only a descriptive study.
Souter 2007 [60]		**X**	One study group.
Wrede 2011 [61]		**X**	One AC group.

Zhang 2008 [62]	**X (No)**	The sizes of the study groups differed. No attempt was made to match the groups.

Overall assessment **Q13: Were important confounding variables not taken into account in the design and/ or analysis (e.g., through matching, stratification, interaction terms, multivariate analysis, or other statistical adjustment such as instrumental variables)?**

**Study**	**Yes, not accounted for or not identified**	**Partially: some variables taken into account or adjustment achieved to some extent**	**No: taken into account**	**Cannot determine**	**Explanation**
Abdou 2010 [17]	**X**				Huge possibility of confounders for example: Confounding by indication for AC, confounding by different baseline characteristics and therapies of the patients before AC surgery.
Ali 2009 [18]	**X**				Huge possibility of confounders for example: Confounding by indication for AC, confounding by different baseline characteristics and therapies of the patients before AC surgery.
Amorim 2008 [19]	**X**				Huge possibility of confounders for example: Confounding by indication for AC, confounding by different baseline characteristics and therapies of the patients before AC surgery.
Andersen 2010 [20]	**X**				Huge possibility of confounders for example: Confounding by indication for AC, confounding by different baseline characteristics and therapies of the patients before AC surgery.

Beez 2013 [21]	**X**		Huge possibility of confounders for example: Confounding by indication for AC, confounding by different baseline characteristics and therapies of the patients before AC surgery.
Bilotta 2014 [10]	**X**		Huge possibility of confounders for example: Confounding by indication for AC, confounding by different baseline characteristics and therapies of the patients before AC surgery.
Boetto 2015 [22]	**X**		Huge possibility of confounders for example: Confounding by indication for AC, confounding by different baseline characteristics and therapies of the patients before AC surgery.
Cai 2013 [23]	**X**		Huge possibility of confounders for example: Confounding by indication for AC, confounding by different baseline characteristics and therapies of the patients before AC surgery.
Chacko 2013 [24]	**X**		Huge possibility of confounders for example: Confounding by indication for AC, confounding by different baseline characteristics and therapies of the patients before AC surgery and confounding by a long recruiting period.
Chaki 2014 [25]	**X**		Huge possibility of confounders for example: Confounding by indication for AC, confounding by different baseline characteristics and therapies of the patients before AC surgery.
Conte 2013 [26]	**X**		Huge possibility of confounders for example: Confounding by indication for AC, confounding by different baseline characteristics and therapies

			of the patients before AC surgery.
Deras 2012 [27]	**X**		Huge possibility of confounders for example: Confounding by indication for AC, confounding by different baseline characteristics and therapies of the patients before AC surgery.
Garavaglia 2014 [28]	**X**		Huge possibility of confounders for example: Confounding by indication for AC, confounding by different baseline characteristics and therapies of the patients before AC surgery.
Gonen 2014 [29]	**X**		Huge possibility of confounders for example: Confounding by indication for AC, confounding by different baseline characteristics and therapies of the patients before AC surgery.
Grossman 2007 [30]	**X**		Huge possibility of confounders for example: Confounding by indication for AC, confounding by different baseline characteristics and therapies of the patients before AC surgery.
Grossman 2013 [31]	**X**		Huge possibility of confounders for example: Confounding by indication for AC, confounding by different baseline characteristics and therapies of the patients before AC surgery and confounding by a long recruiting period.
Hansen 2013 [33]	**X**		Huge possibility of confounders for example: Confounding by indication for AC, confounding by different baseline characteristics and therapies of the patients before AC surgery and confounding by a long recruiting period.

Hervey-Jumper 2015 [34]	**X**		Huge possibility of confounders for example: Confounding by indication for AC, confounding by different baseline characteristics and therapies of the patients before AC surgery and confounding by a long recruiting period.
Ilmberger 2008 [35]		**X**	Several confounders were taken into account, for the primary outcome.
Kim 2009 [37]	**X**		Huge possibility of confounders for example: Confounding by indication for AC, confounding by different baseline characteristics and therapies of the patients before AC surgery and confounding by a long recruiting period and the consecutive learning curve of the AC team.
Li 2015 [38]	**X**		Huge possibility of confounders for example: Confounding by indication for AC, confounding by different baseline characteristics and therapies of the patients before AC surgery and confounding by a long recruiting period and the consecutive learning curve of the AC team.
Lobo 2007 [39]	**X**		Huge possibility of confounders for example: Confounding by indication for AC, confounding by different baseline characteristics and therapies of the patients before AC surgery.
Low 2007 [40]	**X**		Huge possibility of confounders for example: Confounding by indication for AC, confounding by different baseline characteristics and therapies of the patients before AC surgery.
McNicholas 2014 [41]	**X**		Huge possibility of confounders for example: Confounding by indication for AC, confounding by different baseline characteristics and therapies

			of the patients before AC surgery.
Nossek 2013 [42]	**X**		Huge possibility of confounders for example: Confounding by indication for AC, confounding by different baseline characteristics and therapies of the patients before AC surgery and confounding by a long recruiting period.
Nossek 2013 [43]		**X**	The association of patients on multiple antiepileptic drugs and intraoperative seizures was taken into account of analysis but for example confounding by other baseline differences, the indication for AC and the long recruiting period not.
Olsen 2008 [44]	**X**		Huge possibility of confounders for example: Confounding by indication for AC, confounding by different baseline characteristics and therapies of the patients before AC surgery.
Ouyang 2013 [45]		**X**	Multivariable logistic regression analysis for some possible baseline confounding variables was performed. But there remains the possibility of confounding by indication for AC and the long recruitment period.
Ouyang 2013 [46]		**X**	Multivariable logistic regression analysis for some possible baseline confounding variables was performed. But there remains the possibility of confounding by indication for AC and the long recruitment period.
Pereira 2008 [47]	**X**		Huge possibility of confounders for example: Confounding by indication for AC, confounding by different baseline characteristics and therapies of the patients before AC surgery.

Peruzzi 2011 [48]		**X**		The variables checked for confounding effects included sex, KPS score, primary re-section, tumour volume, surgery time, diagnosis, location, intraoperative MR imaging, and age.
Pinsker 2007 [49]	**X**			Huge possibility of confounders for example: Confounding by indication for AC, confounding by different baseline characteristics and therapies of the patients before AC surgery.
Rajan 2013 [50]	**X**			Huge possibility of confounders for example: Confounding by indication for AC, confounding by different baseline characteristics and therapies of the patients before AC surgery.
Rughani 2011 [51]	**X**			Huge possibility of confounders for example: Confounding by indication for AC, confounding by different baseline characteristics and therapies of the patients before AC surgery.
Sacko 2010 [52]	**X**			Huge possibility of confounders for example: Confounding by indication for AC, confounding by different baseline characteristics and therapies of the patients before AC surgery.
Sanus 2015 [53]	**X**			Huge possibility of confounders for example: Confounding by indication for AC, confounding by different baseline characteristics and therapies of the patients before AC surgery.
See 2007 [54]	**X**			Huge possibility of confounders for example: Confounding by indication for AC, confounding by different baseline characteristics and therapies of the patients before AC surgery.

Serletis 2007 [55]	**X**	Huge possibility of confounders for example: Confounding by indication for AC, confounding by different baseline characteristics and therapies of the patients before AC surgery and confounding by a long recruiting period.
Shinoura 2013 [57]	**X**	Huge possibility of confounders for example: Confounding by indication for AC, confounding by different baseline characteristics and therapies of the patients before AC surgery.
Sinha 2007 [58]	**X**	Huge possibility of confounders for example: Confounding by indication for AC, confounding by different baseline characteristics and therapies of the patients before AC surgery.
Sokhal 2015 [59]	**X**	Huge possibility of confounders for example: Confounding by indication for AC, confounding by different baseline characteristics and therapies of the patients before AC surgery and confounding by a long recruiting period.
Souter 2007 [60]	**X**	Huge possibility of confounders for example: Confounding by indication for AC, confounding by different baseline characteristics and therapies of the patients before AC surgery.
Wrede 2011 [61]	**X**	Huge possibility of confounders for example: Confounding by indication for AC, confounding by different baseline characteristics and therapies of the patients before AC surgery.
Zhang 2008	**X**	Huge possibility of confounders for example: Confounding by indication

[62]	for AC, confounding by different baseline characteristics and therapies of the patients before AC surgery.
